# Peripheral Biomarkers of Anorexia Nervosa: A Meta-Analysis

**DOI:** 10.3390/nu16132095

**Published:** 2024-06-30

**Authors:** Ya-Ke Wu, Hunna J. Watson, Aaron C. Del Re, Jody E. Finch, Sabrina L. Hardin, Alexis S. Dumain, Kimberly A. Brownley, Jessica H. Baker

**Affiliations:** 1 School of Nursing, University of North Carolina at Chapel Hill, Chapel Hill, NC 27599, USA; 2 Department of Psychiatry, University of North Carolina at Chapel Hill, Chapel Hill, NC 27599, USA; hunna_watson@med.unc.edu (H.J.W.); kim_brownley@med.unc.edu (K.A.B.); 3 School of Psychology, Curtin University, Bentley, WA 6102, Australia; 4 School of Paediatrics, Division of Medicine, The University of Western Australia, Crawley, WA 6009, Australia; 5 Del Re Data & Statistical Consulting, San Diego, CA 91910, USA; stats@acdelre.com; 6 Department of Psychology, University of Kassel, 34127 Kassel, Germany; 7 Department of Psychology, Georgia State University, Atlanta, GA 30302, USA; 8 National Center for PTSD, VA Boston Healthcare System, Boston, MA 02130, USA; sabrinah97@gmail.com; 9 Department of Psychology and Neuroscience, University of North Carolina at Chapel Hill, Chapel Hill, NC 27599, USA; alexissd@live.unc.edu; 10 Equip Health, Inc., P.O. Box 131747, Carlsbad, CA 92013, USA; jbaker@equip.health

**Keywords:** anorexia nervosa, peripheral biomarkers, eating disorder, starvation

## Abstract

The pathogenesis of anorexia nervosa (AN) has been hypothesized to involve several biological systems. However, reliable biomarkers for AN have yet to be established. This study was aimed to identify statistically significant and clinically meaningful peripheral biomarkers associated with AN. A systematic literature search was conducted to identify studies published in English from inception until 30 June 2022. We conducted two-level random-effects meta-analyses to examine the difference between AN and comparison groups across 52 distinct biomarkers and found that acylated ghrelin, adrenocorticotropic hormone (ACTH), carboxy-terminal collagen crosslinks (CTX), cholesterol, cortisol, des-acyl ghrelin, ghrelin, growth hormone (GH), obestatin, and soluble leptin receptor levels were significantly higher in cases of AN compared with those in non-AN controls. Conversely, C-reactive protein (CRP), CD3 positive, CD8, creatinine, estradiol, follicle-stimulating hormone (FSH), free thyroxine, free triiodothyronine, glucose, insulin, insulin-like growth factor 1 (IGF-1), leptin, luteinizing hormone, lymphocyte, and prolactin levels were significantly lower in AN compared with those in non-AN controls. Our findings indicate that peripheral biomarkers may be linked to the pathophysiology of AN, such as processes of adaptation to starvation. Scientific investigation into peripheral biomarkers may ultimately yield breakthroughs in personalized clinical care for AN.

## 1. Introduction

Anorexia nervosa (AN) is an eating disorder characterized by the restriction of energy intake, fear of gaining weight despite low weight, and disturbances in the perception of body weight or shape and the influence of these factors on self-worth [1]. It affects 1.4% of women and 0.2% of men worldwide [2] and has the highest mortality rate of all psychiatric disorders [3,4]. While the pathogenesis of AN involves various biological systems [5], reliable biomarkers for AN have yet to be established. Biomarkers are crucial for investigating illnesses as they provide objective, measurable indicators of biological processes and disease states [6]. They can reveal underlying mechanisms, disease progression, and responses to treatment, offering insights into pathophysiology [6]. In clinical practice, biomarkers can improve diagnosis, predict disease risk, personalize treatment plans, and monitor therapeutic efficacy, ultimately enhancing patient outcomes and enabling precision medicine [7]. Thus, scientific investigation into biomarkers may ultimately yield breakthroughs in biomarker discovery and personalized clinical care for AN. 

Proposed biomarkers (i.e., objective indications of biological processes, medical state, pathogenic processes, or the effects of treatments) for AN span a wide array of biological functions including immune and endocrine (e.g., leptin, ghrelin, and oxytocin), metabolomic (e.g., gut microbiota and fecal metabolites), psychophysiological (e.g., eye-tracking), and nervous system (e.g., olfaction and brain function) functions [5,8]. For example, interleukin-6 shows potential as a marker of AN severity, with symptom improvement linked to normalized levels [9,10]. Combining low leptin levels with body mass index (BMI) may improve AN diagnosis accuracy compared to BMI alone [11]. Low oxytocin levels are of interest as a potential AN biomarker due to their association with eating pathology, social cognition, and mood [12,13]. Research on metabolomic biomarkers indicates that serum and fecal metabolites, along with gut microbiota, can distinguish individuals with AN from healthy controls [11,14,15]. Previous review papers and studies have focused on a single biomarker [13], a group of related biomarkers [16], or a narrative review without meta-analysis [5]. As such, reports that comprehensively investigate biomarkers for AN across various categories and assay methods are lacking.

The aim of this meta-analysis was to compile evidence on peripheral biomarkers studied in AN in order to summarize the findings quantitatively. We compared peripheral biomarkers between individuals diagnosed with and without AN. Here, we define the term peripheral biomarkers as biological markers from blood, serum, plasma, urine, feces, saliva, sweat, body fluids, hair, nails, and cerebrospinal fluid from the brain and spinal cord. We focus on peripheral biomarkers for our review and exclude other types of biomarkers (e.g., image-based, genetic, electrocardiogram, or eye-movement biomarkers) because, comparatively, peripheral biomarkers have interpretable and validated results, can generally be easier and more cost-effective to obtain, and may have greater translatability potential. Such biomarkers are also widely used in AN research across various countries and populations [17]. We also discuss future research directions for identifying biomarkers for clinical use in AN. By quantitatively analyzing data on peripheral biomarkers, this study enhances our understanding of the biological underpinnings of AN, potentially revealing a path to more effective diagnostic tools and treatments. The findings could lead to significant advancements in identifying and managing AN by highlighting specific biomarkers that differentiate affected individuals from those without the condition.

## 2. Materials and Methods

### 2.1. Study Selection and Search Strategy

The current study represents the first sub-analysis derived from a broader systematic review of AN, bulimia nervosa (BN), and binge-eating disorder (BED) in human studies. The search strategy involved searches for AN, BN, and BED, but only the search results from AN were used for this meta-analysis. This sub-analysis specifically focuses on studies containing acute AN cases and non-AN controls. Given the depth of the data obtained for AN and the diagnostic similarities between BN and BED, it was later decided that the meta-analysis results for BN and BED will be presented separately. This systematic review and meta-analysis follows the Preferred Reporting Items for Systematic Reviews and Meta-Analyses (PRISMA) statement [18] (Supplemental Documents S1). PubMed and PsycINFO were searched to identify studies published in English from inception until 30 June 2022.

Inclusion criteria for the overarching systematic review consisted of quantitative studies that (1) compared one or more peripheral biomarkers between adults (18 years and older) diagnosed with current AN, BN, or BED and controls without these diagnoses and (2) provided statistics means, standard deviations [SD], and the sample size per group, all of which are necessary to calculate effect sizes for use in meta-analytic models. Children were excluded from the analysis due to the potential biological and developmental differences between adults and children. Indeed, children’s hormonal biological systems may not be fully developed (e.g., pre- vs. post-puberty), which could introduce variability and confound findings that we would be unable to account for in the analysis. We focus on adults in order to minimize the influence of such developmental factors on the results. We encourage future, similar work to be completed in younger age groups.

The definition of AN was based on criteria provided in individual studies through formal psychiatric structured diagnostic interview based on versions of the Diagnostic and Statistical Manual of Mental Disorders or Feigner’s diagnostic criteria published in 1972 [19]. We focused on current AN cases and excluded recovered or partially recovered cases from our analysis to capture levels of biomarkers during acute illness. Finally, we defined the control group as healthy adults (18 years and older) without a diagnosis of AN. We excluded studies that met the following criteria: qualitative research, review studies, case reports, expert opinions, editorials, animal studies, neuroimaging studies, reports on eating disorder symptoms rather than diagnoses, reports on AN without peripheral biomarker outcomes or a healthy control group, reported research protocol without results, and studies where English full text does not exist after contacting corresponding authors. We excluded genetic studies since this was outside of the scope of our review.

The complete search strategies in PubMed and PsycINFO are presented in Supplementary Table S1. Medical Subject Headings (MeSH) terms were used in PubMed to increase search accuracy. Reference lists of the studies collected were searched for additional eligible studies. The literature search was performed by one individual researcher (YKW). Two authors independently reviewed articles (YKW and JHB). Covidence systematic review software was used to manage the retrieved studies and provide organization (Veritas Health Innovation, Melbourne, Australia. Available at www.covidence.org (accessed on 6 June 2024)) by finding duplicates, screening studies, coding reasons for exclusion, and assessing risk of bias. All the studies identified following the database search were uploaded to Covidence. Before the full-text review, two researchers (YKW and JHB) independently screened titles and abstracts to identify studies of relevance and excluded studies with improper topics and abstracts. Studies approved by both researchers proceeded to the full-text review process. The two researchers then independently assessed the full-text articles for suitability, and studies approved by both researchers were included in data extraction. The two reviewers met to resolve any discrepancies found during the process.

### 2.2. Data Extraction

The following information was extracted by three authors (YKW, JF, and SLH) and two volunteer research assistants to organize the data and prepare it for analysis: authors and year published, study location, study design, participant source and sampling method, number of participants, percentage of female participants, race, mean age and BMI, diagnostic criteria and procedure for AN diagnosis, mean duration of illness (years), psychotropic medications used (yes/no), name and source of biomarkers, method of specimen collection, type of assay, and mean and SD of biomarkers. Descriptive statistics were used to obtain the sum, mean, SD, range, and percentage of study and sample characteristics. All data extraction and management were conducted in Microsoft Excel [20].

### 2.3. Quality Appraisal

The quality of retrieved studies was assessed with a 9-item Joanna Briggs Institute Meta-Analysis of Statistics Assessment and Review Instrument (JBI-MAStARI) critical appraisal tool [21]. This tool is designed to evaluate the general quality of research, including items such as the method of sample selection, method for dealing with confounding factors, reliability of outcome measures, and statistical analysis. Each item was classified as present “Yes”, absent “No”, or “Unclear” for each included study, and then each response was recorded as +1, 0, or −1, respectively. Items were classified as “non-applicable” if they were not relevant to the study design and were scored as 0. The total score ranged from 0 to 9, with a higher score indicating higher quality. 

### 2.4. Calculating Effect Sizes

Means, standard deviations, and Ns of all included biomarkers within studies were used to calculate standardized mean differences (SMDs) between those with and without AN [22]. The basic formula for Cohen’s *d*, which estimates the SMD between two groups, requires data on the mean and standard deviation of the outcome scores for each condition:

(1) $$d=\frac{M_{T}-M_{C}}{S}$$

where *M_T_* and *M_C_* are the means for the treatment (AN group) and control groups, respectively, and *S* is an estimate of the population standard deviation for the outcome variable. Typically, meta-analysts use the post-test means and standard deviations, but in our case, we used the baseline mean and standard deviations in all effect size calculations. In meta-analysis, effect sizes are weighted by the inverse of the sampling variance (*V*), which reflects how precisely *d* estimates the population SMD. The variance of *d* is computed as

(2) $$V_{d}=\frac{1}{n_{T}}+\frac{1}{n_{C}}+\frac{0.5d^{2}}{n_{T}+n_{C}},$$

where *n_T_* and *n_C_* are the sample sizes for treatment and comparison groups, respectively, and *d* is computed by Equation (1). Because d is a slightly biased estimator of the population SMD when sample sizes are small, Hedges (1981; Hedges and Olkin, 1985) recommended calculating a bias-corrected SMD estimator called Hedges’ *g*. The conversion from *d* to *g* uses a bias correction factor:

(3) $$J=1-\frac{3}{4\cdot df-1},$$

which is used to derive unbiased estimates of the SMD and its sampling variance:

(4a) g=J·d

(4b) $$V_{g}=J^{2}\cdot V_{d}.$$

### 2.5. Meta-Analysis

Multilevel random-effects meta-analyses were conducted using the meta-analysis R packages metafor [23] and MAd [24]. A summary (omnibus) effect size (ES) was calculated for each biomarker, which is a weighted average of the individual study ESs, where each study is weighted by the inverse of the variance, which is mostly a function of sample size in the study—larger studies are weighted heavier in the omnibus test. There are several modeling approaches for calculating the summary effect, and the choice of procedure depends on the assumptions made about the distribution of effects.

We chose to utilize a multilevel random-effects approach, which assumes that between-study variance is not 0 and ES differences are due to both sampling error and true ES differences in the population. That is, there is distribution of “true” population ESs. The random-effect model considers the studies included in the analysis as a sample from a larger universe of studies that could be conducted. The results from random-effects analyses are generalizable beyond the included set of studies and can be used to infer what would likely happen if a new study were conducted. 

In addition, the random-effects model is generally preferred because most meta-analyses include studies that are not identical in their methods and/or sample characteristics. Differences in methods and sample characteristics between studies will likely introduce variability among the true ESs and should be modeled accordingly with a random-effects procedure, given by the following equation:

(5) $$\theta_{i}=\mu +v_{i}^{*}$$

where *θ_i_* is the true effect for study *i*, which is assumed to be unbiased and normally distributed, μ is the average true ES, and $v_{i}^{*}=v_{i}+\tau^{2}$, where the variance of the within-study errors *ν_j_* is known, and the between-study error *τ*^2^ is unknown and estimated from the studies included in the analysis.

Biomarkers with fewer than three studies contributing at least one ES were dropped from the analyses (19 biomarkers had fewer than three studies available). Extreme outlier ESs (g ≤ −4 or g ≥ 4) were also dropped from analyses (j = 14) but did not change the number of biomarkers available for analysis. Fifty-two distinct multilevel meta-analytic models were conducted, one for each included biomarker. All the multilevel models accounted for potential dependencies in the data (multiple within-study effect sizes within a given biomarker category) by specifying the correct multilevel meta-analytic model. Homogeneity was assessed with the Q-statistic and indexed as a percentage of variance with the *I*^2^ statistic [25,26]. *I*^2^ is an index of heterogeneity computed as a percentage that reflects the proportion of between-study variability. Potential publication bias was examined by visually inspecting the asymmetry in a funnel plot [27], along with precision-effect estimates for SE tests [28]. Three-parameter selection models were also employed to assess the confluence with the other publication bias tests. These models were also used to adjust the overall omnibus (overall mean) effect sizes to account for possible publication bias across the biomarkers [29]. 

Although the studies may have had varied designs (e.g., different follow-up times and types of interventions), only baseline biomarker measures were used and analyzed. Only 30 studies reported an AN subtype (i.e., restricting and binge-eating/purging subtypes). Therefore, we combined the restricting and binge-eating/purging subtypes into one group for a given biomarker from the same study using Cochrane’s formula to combine two groups of Ns, means, and SDs with the following calculations: combined N = N1 + N2; combined mean = (N1 × M1 + N2 × M2)/(N1 + N2); and combined standard deviation = sqrt(((N1 − 1) × SD1 × SD1 + (N2 − 1) × SD2 × SD2 + N1 × N2/(N1 + N2) × (M1 × M1 + M2 × M2 − 2 × M1 × M2))/(N1 + N2 − 1)), where N = number of participants, M = mean, and SD = standard deviation [30]. Also, in some studies, the control participants were divided into different categories based on their body weights (i.e., normal BMI, constitutionally thin, or obese). However, not all biomarkers were analyzed by body weight in control groups. Therefore, we also combined control participants of different body weights into one group for a given biomarker in the same study using the same formula as above. To ensure that no overlapping results were analyzed, we cross-examined the included studies published by the same authors or organizations. We found 34 studies that likely used the same source of participants (i.e., possibly the same source of participants but published in more than one paper). If study participants were possibly overlapping across more than one paper, we chose the paper with the most participants for a given biomarker.

## 3. Results

### 3.1. Search Outcome

**Figure 1:**
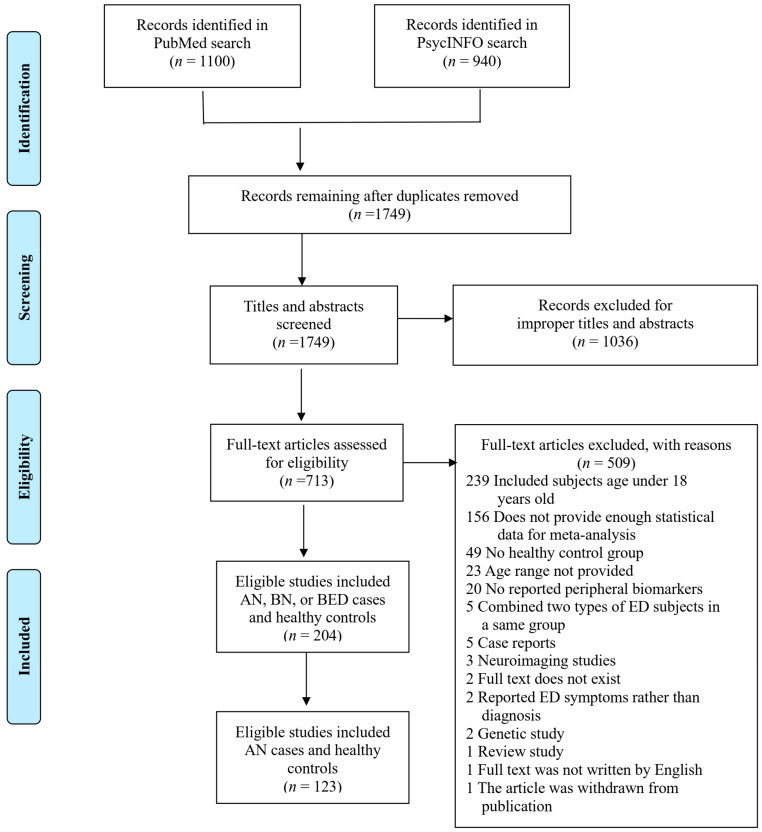
Flowchart of the inclusion procedure in a PRISMA diagram. AN = anorexia nervosa, BN = bulimia nervosa, BED = binge-eating disorder, ED = eating disorder.

As the original search included individuals with AN, BN, and BED, the PRISMA flowchart in Figure 1 summarizes the study selection process of all three conditions and the final result for AN only. The search strategy resulted in 2040 studies for initial consideration in the meta-analysis. After removing 291 duplicate studies, 1749 studies remained. After screening titles and abstracts, 1036 studies were excluded, and 713 studies underwent full-text assessment for eligibility. By examining the full texts of the remaining 713 studies, 509 studies were excluded due to the reasons listed in Figure 1, leaving 204 studies that included adults with AN, BN, or BED and healthy controls. Out of the 204 studies, we included 123 studies reporting peripheral biomarkers from AN cases and control groups in the current study (Supplemental Document S2).

### 3.2. Characteristics of Included Studies

A summary of the included studies’ characteristics is provided in Supplemental Table S2. A total of 5401 female participants (AN = 2700, controls = 2701, male participants = 0) from 13 countries (Supplemental Figure S1) were included, with a mean ± SD age of 25.02 ± 3.35 years across the studies. Most of the studies were conducted in the United States, Japan, and Italy, with a cross-sectional design (65.04%, 80 studies), longitudinal design with an intervention (26.83%, 33 studies), or longitudinal design without an intervention (8.13%, 10 studies). Most participants were recruited through clinical treatment programs (88.62%, 109 studies), with 11 studies recruiting from communities and 3 studies not reporting the recruitment source. 

Thirteen studies in the review reported participant’s races, with 100% Caucasian participants in nine studies (*n* = 576), 100% Asians in three studies (*n* = 142), and 33.33% Caucasian plus unknown race in one study (*n* = 18). The mean BMI of AN cases across the studies was 15.53 ± 1.62 kg/m^2^, and the range was 12.00 to 19.87 kg/m^2^. The mean BMI of healthy controls across the studies was 22.20 ± 3.48 kg/m^2^, and the range was 15.90 to 43.63 kg/m^2^. The most used diagnostic criteria were the Diagnostic and Statistical Manual of Mental Disorders-IV criteria for AN (*n* = 83 studies). Forty-five studies reported the duration of illness of AN among cases (mean ± SD = 5.95 ± 2.11 years), and eleven studies reported psychotropic medications used among participants. 

The total number of biomarkers identified was 52 biomarkers in our meta-analysis. Approximately 95% of biomarker sources were blood (serum, plasma, or non-specific), 4% were urine, and 1% were saliva. The most common assay method was radioimmunoassay (*n* = 63 studies, Supplemental Figure S2).

### 3.3. Quality of Studies

Overall, 93% of the studies (*n* = 115) received a quality rating of 5 or 6 (Supplemental Table S3). The mean score of the quality assessment for all 123 studies was 5.49 (±0.69), and the range was 3 to 7. None of the included studies reported using a random sample. The mean number of total participants per study was 51.96 (±42.70), with a range of 11 to 239. Most (*n* = 111) studies in this review reported sample sizes less than 100. Only two studies performed power analyses to justify sample size [31,32], making it difficult to determine if the sample size was sufficient for most studies. Only five studies clearly discussed confounding factors (i.e., age, baseline biomarkers levels, BMI, body fat, and disease status) and controlled for them in their analyses. Overall, the limitations of the included studies primarily consisted of small convenience samples without sufficient sample size justification, as well as a failure to account for potential confounding variables in analyses.

### 3.4. Meta-Analysis Results

**Figure 2:**
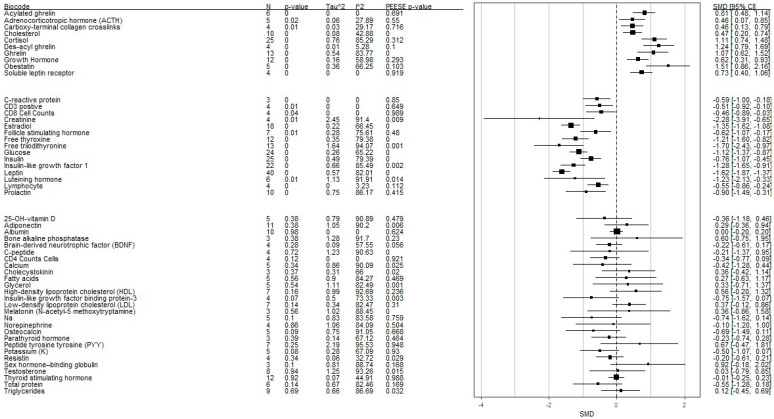
Forest plot of all biomarkers’ summary effect sizes. The forest plot was organized by the direction of effect for biomarkers higher in the AN group, those higher in the comparison group, and those that were not statistically significant. N = number of studies; Tau^2^ = Tau-squared for examining between-study heterogeneity; *I*^2^ = heterogeneity index for examining between-study heterogeneity; PEESE *p*-value = *p*-value for the precision-effect estimate for the SE publication bias test (<0.05 indicates possible publication bias); SMD = standardized mean difference; 95% CI = 95% confidence interval.

Table 1, Figure 2, and Supplemental Document S3 present meta-analysis results for the 52 peripheral biomarkers. The Q homogeneity test was significant for 25-OH-vitamin D, adiponectin, bone alkaline phosphatase, C-peptide, calcium, cholecystokinin, cholesterol, cortisol, creatinine, estradiol, fatty acids, follicle-stimulating hormone (FSH), free thyroxine, free triiodothyronine, ghrelin, glucose, glycerol, GH, high-density lipoprotein cholesterol, insulin, IGF-1, insulin-like growth factor-binding protein-3 (IGFBP-3), leptin, low-density lipoprotein cholesterol, luteinizing hormone, melatonin, Na, norepinephrine, obestatin, osteocalcin, parathyroid hormone, peptide tyrosine tyrosine, potassium, prolactin, sex hormone-binding globulin, testosterone, thyroid-stimulating hormone, total protein, and triglycerides, indicating between-study heterogeneity for studies investigating these biomarkers. 

A significant difference between AN and controls was found in 26 of the 52 biomarkers in the omnibus tests. Acylated ghrelin (g = 0.81 [lower CI = 0.48; upper CI =1.14]), adrenocorticotropic hormone (ACTH, g = 0.46 [lower CI = 0.06; upper CI = 0.86]), carboxy-terminal collagen crosslinks (CTX, g = 0.46 [lower CI = 0.13; upper CI = 0.78]), cholesterol (g = 0.47 [lower CI = 0.20; upper CI = 0.74]), cortisol (g = 1.11 [lower CI = 0.73; upper CI = 1.49]), des-acyl ghrelin (g = 1.24 [lower CI = 0.78; upper CI = 1.70]), ghrelin (g = 1.07 [lower CI = 0.62; upper CI = 1.52]), GH (g = 0.62 [lower CI = 0.31; upper CI = 0.93]), obestatin (g = 1.51 [lower CI = 0.86; upper CI = 2.16]), and soluble leptin receptor (g = 0.73 [lower CI = 0.40; upper CI = 1.06]) were significantly higher in the AN group compared with the control group. 

On the other hand, CRP (g = −0.59 [lower CI = −0.99; upper CI = −0.18]), CD3 positive (g = −0.51 [lower CI = −0.91; upper CI = −0.10]), CD8 (g = −0.46 [lower CI = −0.89; upper CI = −0.03]), creatinine (g = −2.28 [lower CI = −3.90; upper CI = −0.65]), estradiol (g = −1.35 [lower CI = −1.62; upper CI = −1.08]), FSH (g = −0.62 [lower CI = −1.08; upper CI = −0.17]), free thyroxine (g = −1.21 [lower CI = −1.60; upper CI = −0.82]), free triiodothyronine (g = −1.70 [lower CI = −2.43; upper CI = −0.97]), glucose (g = −1.12 [lower CI = −1.38; upper CI = −0.86]), insulin (g = −0.76 [lower CI = −1.08; upper CI = −0.45]), IGF-1 (g = −1.28 [lower CI = −1.66; upper CI = −0.91]), leptin (g = −1.62 [lower CI = −1.89; upper CI = −1.36]), luteinizing hormone (g = −1.23 [lower CI = −2.12; upper CI = −0.33]), lymphocyte (g = −0.55 [lower CI = −0.87; upper CI = −0.23]), and prolactin (g = −0.90 [lower CI = −1.49; upper CI = −0.30]) were significantly lower in the AN group compared with the control group. After adjusting for potential publication bias, ACTH, cortisol, ghrelin, GH, luteinizing hormone, and prolactin became non-significant between the AN and control groups (Table 1, column “g+ adj”).

### 3.5. Publication Bias

The funnel plots (Supplemental Document S4) for all biomarker categories were asymmetric for 25-Oh-vitamin D, adiponectin, ACTH, bone alkaline phosphatase, brain-derived neurotrophic factor, calcium, cholecystokinin, cholesterol, C-peptide, CRP, des-acyl ghrelin, glucose, glycerol, GH, norepinephrine, parathyroid hormone, prolactin, resistin, sex hormone-binding globulin, total protein, and triglycerides, indicating the possibility of publication bias. The PEESE and three-parameter selection model tests for publication bias (Table 1, columns X^2^ and X^2^ *p*) showed significant results for adiponectin, c-peptide, calcium, cholecystokinin, cholesterol, creatinine, estradiol, free thyroxine, free triiodothyronine, ghrelin, glucose, glycerol, insulin, IGF-1, IGFBP-3, leptin, low-density lipoprotein cholesterol, melatonin, peptide tyrosine tyrosine, resistin, testosterone, and triglycerides, suggesting the possibility of publication bias for these biomarkers.

## 4. Discussion

### 4.1. Key Findings

To our knowledge, this is the first meta-analysis that examines multiple peripheral biomarkers in individuals with acute AN compared with those in non-AN individuals. Not surprisingly, metabolic-related biomarkers received high attention from researchers due to their possible roles in AN pathophysiology. Our results showed that all forms of ghrelin were higher in AN and leptin was lower in AN compared with non-AN controls. These results are consistent with a previous meta-analysis and a literature review [17,33]. Ghrelin is an orexigenic peptide that mostly produced in the stomach, increases hunger signals in the brain, regulates insulin release, stimulates the release of growth hormones from the pituitary gland, and is increased during fasting and suppressed by food intake [34,35]. However, individuals with AN appear to be able to resist ghrelin’s appetite-stimulating effects despite the high ghrelin in their body, known as the hypothesis of ghrelin resistance [36,37]. The mechanism of ghrelin resistance in AN is not fully understood, but the theory of hypothalamic ghrelin signaling reveals that ghrelin influences food intake by binding with growth hormone secretagogue receptor in the hypothalamic cells with the help from functional cannabinoid receptor 1 to regulate food intake [38]. Researchers could investigate whether the low binding of ghrelin to growth hormone secretagogue receptors influences AN individuals’ response to ghrelin’s appetite-stimulating effects in future studies. Furthermore, low ghrelin-reactive autoantibodies in individuals with AN may contribute to the high acylated ghrelin (i.e., essential for appetite regulation) and des-acyl ghrelin (i.e., may antagonize the acylated ghrelin) due to insufficient plasma binding between the autoantibodies and the two forms of ghrelin [36]. Researchers should investigate whether ghrelin-reactive autoantibodies contribute to high ghrelin levels in AN in the future.

Our results concerning higher GH and lower IGF-1 in the AN group compared with non-AN group are consistent with a narrative review that suggested that individuals with AN exhibit higher GH and lower IGF-1 [39]. Low body fat is associated with increased GH levels, and GH and IGF-1 influence systemic glycemic control [40]. According to the theory of the GH–IGF-1 axis, high GH but low IGF-1 with low body fat (such as in AN) indicates GH resistance, which means the downregulation of the GH receptor with low GH-binding protein, despite the high GH in individuals with low body fat [39]. 

Menstrual irregularities and estrogen deficiency are common consequences of AN due to malnutrition and low body fat [41]. Our results showed low estradiol in AN, which is consistent with these observations. As fat converts androstenedione to estrone and testosterone to estradiol, the lack of adipose tissue may contribute to the hypoestrogenic state in individuals with AN [42]. Furthermore, low FSH was also associated with AN in the present study and may also contribute to low estradiol since FSH converts androgens into estradiol by stimulating granulosa cells in ovarian follicles [43].

We found alterations in biomarkers that may be associated with starvation adaptation processes. Findings showed lower free thyroxine, free triiodothyronine, prolactin, and higher ACTH, cortisol, and CTX in AN compared to controls. These findings suggest possible adaptation processes to cope with starvation [42]. A low thyroid and prolactin function could lower metabolic rate, reduce fertility, and conserve energy in starvation [42]. Additionally, thyroid hormones and prolactin may negatively influence mood regulation and cognitive function in individuals with psychotic disorders, while low thyroid function was associated with cognitive decline in individuals with mood disorders and schizophrenia [44], as well as more severe depressive symptoms in AN than in controls [45]. High plasma prolactin levels are associated with poorer cognitive functioning in early psychosis [46]; however, the effects of prolactin on cognitive function are still unclear [44]. Increased ACTH releases cortisol, which stimulates gluconeogenesis and breaks down protein and fat to provide metabolites that can be converted into glucose in the liver as an energy source [47]. Furthermore, CTX is one of the bone turnover markers; a high CTX level indicates high osteoclastic activity and is associated with increased fracture risk [48]. Our finding of high CTX in AN is consistent with the previous research [49]. It is likely that the high CTX in individuals with AN is due to the low calcium and vitamin D3 intake that is often observed in individuals with AN, which increases the risk of osteoporosis and fractures [49]. However, our results show no statistically significant difference in calcium and vitamin D levels between the AN and non-AN groups.

We observed alterations in biomarkers of immune function that are consistent with prior studies. The significantly low CRP in AN from our results is consistent with a previous meta-analysis [50]. CRP increased when BMI and body fat mass increased and vice versa [51]. As individuals with AN typically manifest low BMI and body fat mass [1], this may explain the low CRP observed in this group. Furthermore, low lymphocytes, CD3 positive, and CD8 cells are likely a result of body mass loss [52,53] and protein-calorie malnutrition (i.e., a lack of protein or calorie consumption and loss of muscle or fat) [54] in AN individuals. Previous studies showed that lymphocyte subsets (e.g., CD3, CD4, CD8) were negatively associated with BMI [52,53]. Also, protein-calorie malnutrition is one of the common causes of immune deficiencies, as it reduces lymphocyte transformation in response to mitogens and leads to immunological abnormalities. [54]. However, it is unclear whether low levels of lymphocytes and lymphocyte subsets in individuals with AN reflect a resistance to infections that were controversially observed in AN individuals despite their poor health conditions [55,56] or an adaptive mechanism to preserve energy by decreasing the immune response in spite of possible infections [52].

### 4.2. Limitations

Although the findings from this meta-analysis are informative, relevant, and based on strict and recommended meta-analytic procedures, there are limitations. First, due to a limited number of studies reporting AN subtype, we combined participants with restricting and binge-eating/purging subtypes, which may limit our understanding of the potential differences in biological mechanisms between the two subtypes. Second, several biomarkers had statistically significant Q homogeneity test results. These results indicate heterogeneity among the included studies, which may be due to differences in methodology or other unexamined potential moderating variables (e.g., average age in the sample) [57]. Regardless of heterogeneity across some biomarkers, several differences remained statistically significant, indicating that although there is variation across studies, there were significant differences between AN and comparison groups, even when addressing the variation, sample sizes, and potential publication bias across studies. 

Considering the broad scope of the current project, we chose to focus on the overall omnibus effect sizes across the 52 biomarker categories and encourage researchers to investigate potential sources of heterogeneity, perhaps across a subset of these biomarkers, in future studies. Subgroup and moderator analyses might help to form a clearer picture of which biomarkers are associated with AN and how. However, these types of analyses require the number of available studies to be sufficiently large, which was not the case across several biomarker categories in our analyses. Third, since AN most often occurs during adolescence [58], excluding this age group from our review may limit our understanding of the disorder’s early onset and developmental aspects. Fourth, publications in other languages may have been missed since only English articles were searched. Finally, it is worth noting that differences in biomarkers in our findings may be symptomatic of AN, rather than causal for AN. Nevertheless, our findings are still useful for understanding and defining AN’s recovery process, such as identifying key biomarkers to develop a metabolic definition of recovery and ultimately contribute to developing targeted interventions to support long-term recovery.

## 5. Conclusions

In conclusion, this study provides valuable insights into peripheral biomarkers for individuals with AN. We identified significant alterations in biomarkers related to metabolic, hormonal, and immune function in individuals with AN compared to healthy controls. While many of our findings align with previous research, caution is warranted given the limited number of studies on certain biomarkers, the heterogeneity among included studies, and the lack of diversity in participant characteristics. Future meta-analysis should consider examining biomarkers in individuals with AN who also exhibit other mental illnesses, such as mood disorders, to better understand the overlapping and distinct biological mechanisms that contribute to comorbidity and to improve targeted treatment strategies. Nonetheless, this research lays a foundation for future studies to elucidate the role of peripheral biomarkers in AN pathophysiology and clinical management. Ultimately, translating the knowledge of peripheral biomarkers in AN from basic science to clinical practice holds promise for improving the diagnosis and treatment of individuals with AN.

## Figures and Tables

**Table 1:** Summary of meta-analysis findings of 52 peripheral biomarkers.

Peripheral Biomarkers	k	g^+^	SE (g^+^)	CI L	CI U	*p*	Tau^2^	*I* ^2^	z (PEESE)	z *p* (PEESE)	X^2^ (TPSM)	X^2^ *p* (TPSM)	g+ adj	*p* adj	CI L adj	CI U adj
25-Oh-Vitamin D	5	−0.36	0.42	−1.18	0.45	0.38	0.79	90.89	−0.708	0.479	0.163	0.687	−0.513	0.262	−1.409	0.384
Acylated Ghrelin	6	0.81	0.17	0.48	1.14	0	0	0	0.398	0.691	0.135	0.713	0.735	0.006	0.212	1.258
Adiponectin	11	0.29	0.33	−0.36	0.93	0.38	1.05	90.2	−2.774	0.006	8.288	0.004	−0.770	0.123	−1.749	0.209
Adrenocorticotropic Hormone	5	0.46	0.2	0.06	0.86	0.02	0.06	27.89	−0.598	0.550	0.184	0.668	0.385	0.056	−0.009	0.779
Albumin	10	0	0.1	−0.18	0.19	0.98	0	0	0.490	0.624	0.528	0.467	−0.036	0.732	−0.245	0.172
Bone Alkaline Phosphatase	3	0.6	0.69	−0.74	1.95	0.38	1.28	91.7	1.201	0.230	0.893	0.345	−0.113	0.906	−1.974	1.749
Brain-Derived Neurotrophic Factor	4	−0.22	0.2	−0.61	0.18	0.28	0.09	57.55	−1.914	0.056	NA	NA	NA	NA	NA	NA
Calcium	5	−0.42	0.44	−1.28	0.44	0.34	0.86	90.09	−4.158	0.000	5.045	0.025	−1.016	0.082	−2.161	0.130
Carboxy-Terminal Collagen Crosslinks	4	0.46	0.17	0.13	0.78	0.01	0.03	29.17	0.189	0.850	0.003	0.955	−0.586	0.004	−0.990	−0.183
CD3 Positive	4	−0.51	0.21	−0.91	−0.1	0.01	0	0	0.456	0.649	0.021	0.884	−0.504	0.016	−0.913	−0.095
CD4 Cell Counts	4	−0.34	0.22	−0.77	0.08	0.12	0	0	−0.100	0.921	0.065	0.798	−0.333	0.136	−0.771	0.105
CD8 Cell Counts	4	−0.46	0.22	−0.89	−0.03	0.04	0	0	0.013	0.989	0.032	0.858	−0.454	0.041	−0.891	−0.018
Cholecystokinin	3	0.36	0.4	−0.42	1.13	0.37	0.31	66	−2.246	0.025	7.106	0.008	−0.983	0.002	−1.620	−0.347
Cholesterol	10	0.47	0.14	0.2	0.74	0	0.08	42.88	−0.363	0.716	NA	NA	NA	NA	NA	NA
Cortisol	25	1.11	0.19	0.73	1.49	0	0.76	85.29	2.335	0.020	2.835	0.092	−0.129	0.301	−0.372	0.115
C-Peptide	4	−0.21	0.59	−1.37	0.95	0.72	1.23	90.63	3.524	0.000	0.000	0.983	0.453	0.331	−0.461	1.367
C-Reactive Protein	3	−0.59	0.21	−0.99	−0.18	0	0	0	1.011	0.312	0.780	0.377	−0.586	0.004	−0.990	−0.183
Creatinine	4	−2.28	0.83	−3.9	−0.65	0.01	2.45	91.4	−2.614	0.009	0.156	0.693	−2.174	0.008	−3.781	−0.566
Des-Acyl Ghrelin	4	1.24	0.23	0.78	1.7	0	0.01	5.28	−1.644	0.100	0.603	0.437	1.395	0.000	0.825	1.966
Estradiol	18	−1.35	0.14	−1.62	−1.08	0	0.22	66.45	−3.656	0.000	0.013	0.908	−1.346	0.000	−1.614	−1.077
Fatty Acids	5	0.27	0.46	−0.63	1.18	0.56	0.9	84.27	0.724	0.469	0.341	0.559	−0.026	0.964	−1.183	1.130
Follicle-Stimulating Hormone	7	−0.62	0.23	−1.08	−0.17	0.01	0.28	75.61	−0.707	0.480	0.430	0.512	−0.564	0.040	−1.101	−0.027
Free Thyroxine	12	−1.21	0.2	−1.6	−0.82	0	0.35	79.38	−3.574	0.000	0.092	0.762	−1.194	0.000	−1.581	−0.808
Free Triiodithyronine	13	−1.7	0.37	−2.43	−0.97	0	1.64	94.07	−3.259	0.001	1.366	0.242	−1.470	0.007	−2.536	−0.405
Ghrelin	13	1.07	0.23	0.62	1.52	0	0.54	83.77	4.958	0.000	3.290	0.070	0.515	0.312	−0.483	1.514
Glucose	24	−1.12	0.13	−1.38	−0.86	0	0.26	65.22	−4.171	0.000	0.138	0.711	−1.112	0.000	−1.379	−0.846
Glycerol	5	0.33	0.53	−0.71	1.36	0.54	1.11	82.49	3.303	0.001	0.919	0.338	1.356	0.388	−1.721	4.433
Growth Hormone	12	0.62	0.16	0.31	0.93	0	0.16	58.98	1.052	0.293	0.675	0.411	0.464	0.056	−0.012	0.941
Hdl Cholesterol	7	0.56	0.39	−0.22	1.33	0.16	0.99	92.69	−1.184	0.236	0.741	0.389	0.965	0.099	−0.182	2.113
Insulin	25	−0.76	0.16	−1.08	−0.45	0	0.49	79.39	−3.603	0.000	0.002	0.965	−0.764	0.000	−1.110	−0.418
Insulin-Like Growth Factor 1	22	−1.28	0.19	−1.66	−0.91	0	0.66	85.49	−3.131	0.002	1.425	0.233	−1.358	0.000	−1.703	−1.014
Insulin-Like Growth Factor-Binding Protein−3	4	−0.75	0.42	−1.57	0.06	0.07	0.5	73.33	−2.959	0.003	0.196	0.658	−0.680	0.099	−1.488	0.128
Ldl Cholesterol	7	0.37	0.25	−0.12	0.85	0.14	0.34	82.47	−3.628	0.000	3.130	0.077	−1.670	0.000	−1.906	−1.433
Leptin	40	−1.62	0.13	−1.89	−1.36	0	0.57	82.01	−1.015	0.310	7.467	0.006	−1.504	0.032	−0.129	−2.880
Luteinizing Hormone	6	−1.23	0.46	−2.12	−0.33	0.01	1.13	91.91	−2.454	0.014	0.685	0.408	−1.012	0.139	−2.353	0.329
Lymphocyte (%)	4	−0.55	0.16	−0.87	−0.23	0	0	3.23	−1.588	0.112	0.008	0.930	−0.547	0.001	−0.863	−0.232
Melatonin (N-Acetyl−5 Methoxytryptamine)	3	0.36	0.62	−0.86	1.57	0.56	1.02	88.45	−4.409	0.000	0.145	0.703	0.630	0.475	−1.098	2.359
Na	5	−0.74	0.45	−1.63	0.15	0.1	0.83	83.58	−0.306	0.759	0.903	0.342	−0.397	0.673	−2.242	1.448
Norepinephrine	4	−0.1	0.56	−1.2	1	0.86	1.06	84.09	0.669	0.504	3.691	0.055	2.132	0.468	−3.628	7.892
Obestatin	5	1.51	0.33	0.86	2.16	0	0.36	66.25	1.631	0.103	NA	NA	NA	NA	NA	NA
Osteocalcin	5	−0.69	0.41	−1.5	0.11	0.09	0.75	91.05	−0.429	0.668	1.417	0.234	−0.123	0.928	−2.775	2.529
Parathyroid Hormone	3	−0.23	0.26	−0.75	0.29	0.39	0.14	67.12	−0.733	0.464	NA	NA	NA	NA	NA	NA
Peptide Tyrosine Tyrosine	7	0.67	0.58	−0.46	1.8	0.25	2.19	95.53	0.065	0.948	4.655	0.031	2.226	0.022	0.319	4.132
Potassium	5	−0.5	0.29	−1.07	0.07	0.08	0.28	67.09	−0.088	0.930	0.351	0.554	−0.430	0.228	−1.130	0.270
Prolactin	10	−0.9	0.3	−1.49	−0.3	0	0.75	86.17	−0.815	0.415	1.367	0.242	−0.664	0.201	−1.683	0.354
Resistin	4	−0.2	0.21	−0.61	0.21	0.34	0.06	32.72	2.186	0.029	0.092	0.762	−0.208	0.431	−0.724	0.309
Sex Hormone-Binding Globulin	3	0.92	0.56	−0.16	2.01	0.1	0.81	88.74	1.377	0.168	0.003	0.954	0.926	0.161	−0.368	2.220
Soluble Leptin Receptor	4	0.73	0.17	0.4	1.06	0	0	0	0.102	0.919	NA	NA	NA	NA	NA	NA
Testosterone	8	0.03	0.42	−0.78	0.85	0.94	1.25	93.26	2.421	0.015	4.646	0.031	1.918	0.224	−1.171	5.006
Thyroid-Stimulating Hormone	12	−0.01	0.12	−0.24	0.22	0.92	0.07	44.91	0.015	0.988	0.140	0.708	−0.035	0.700	−0.214	0.144
Total Protein	6	−0.55	0.37	−1.28	0.17	0.14	0.67	82.46	−1.375	0.169	1.471	0.225	−0.803	0.006	−1.375	−0.231
Triglycerides	9	0.12	0.29	−0.46	0.69	0.69	0.66	86.69	2.143	0.032	0.162	0.687	−0.033	0.938	−0.863	0.797

Note. k = number of studies; g^+^ = Hedges g; SE (g^+^) = standard error for Hedges g; CI L = lower 95% confidence interval for Hedges g; CI U = upper 95% confidence interval for Hedges g; *p* = *p*-value for Hedges g; Tau^2^ = Tau heterogeneity test; *I*^2^ = *I*^2^ heterogeneity index; z (PEESE) = z-value for precision-effect estimate for SE publication bias test; z *p*-val (PEESE) = *p*-value for the z-value for the precision-effect estimate for the SE publication bias test (<0.05 indicates possible publication bias); X^2^ (TPSM) = chi-squared test for the three-parameter selection model for publication bias; X^2^ *p* (TPSM) = *p*-value for the chi-squared test for the three-parameter selection model; g+ adj = adjusted omnibus (overall mean) effect size from the three-parameter selection model that adjusts for possible publication bias; *p* adj = *p*-value for the adjusted omnibus (overall mean) effect size from the three-parameter selection model; CI L adj = lower 95% confidence interval for the adjusted omnibus effect size; CI U adj = upper 95% confidence interval for the adjusted omnibus effect size.

## Data Availability

The research materials supporting this publication can be accessed by contacting Ya-Ke Wu.

## Supplementary Materials

The following supporting information can be downloaded at: https://www.mdpi.com/article/10.3390/nu16132095/s1, Supplemental Document S1. PRISMA 2020 checklist; Supplemental Table S1. Search strategies; Supplemental Document S2. Reference list for included studies; Supplemental Table S2. Brief summary of included studies; Supplemental Figure S1. List of countries of included studies; Supplemental Figure S2. List of assay methods of included studies; Supplemental Table S3. Quality assessment of the included studies; Supplemental Document S3. Individual forest plots for all 52 peripheral biomarkers; Supplemental Document S4. Individual funnel plots for all 52 peripheral biomarkers.
