# Supplementary material for: Peripheral Biomarkers of Anorexia Nervosa: A Meta-Analysis
Source: Nutrients. 2024 Jun 30;16(13):2095. doi: 10.3390/nu16132095 (PMC11243150; doi:10.3390/nu16132095)
Supplement: Supplementary file 1 [file nutrients-16-02095-s001.zip › nutrients-3071468-supplementary.pdf]

Supplemental document 1. PRISMA 2020 Checklist

Section and Topic	Item #	Checklist item	Location where item is reported
**TITLE**			
Title	1	Identify the report as a systematic review.	Title page
**ABSTRACT**			
Abstract	2	See the PRISMA 2020 for Abstracts checklist.	--
**INTRODUCTION**			
Rationale	3	Describe the rationale for the review in the context of existing knowledge.	Section 1.
Objectives	4	Provide an explicit statement of the objective(s) or question(s) the review addresses.	Section 1.
**METHODS**			
Eligibility criteria	5	Specify the inclusion and exclusion criteria for the review and how studies were grouped for the syntheses.	Section 2.1.
Information sources	6	Specify all databases, registers, websites, organisations, reference lists and other sources searched or consulted to identify studies. Specify the date when each source was last searched or consulted.	Section 2.1. Figure1.
Search strategy	7	Present the full search strategies for all databases, registers and websites, including any filters and limits used.	Supplemental Table1
Selection process	8	Specify the methods used to decide whether a study met the inclusion criteria of the review, including how many reviewers screened each record and each report retrieved, whether they worked independently, and if applicable, details of automation tools used in the process.	Section 2.1.
Data collection process	9	Specify the methods used to collect data from reports, including how many reviewers collected data from each report, whether they worked independently, any processes for obtaining or confirming data from study investigators, and if applicable, details of automation tools used in the process.	Section 2.1. Section 2.2.
Data items	10a	List and define all outcomes for which data were sought. Specify whether all results that were compatible with each outcome domain in each study were sought (e.g. for all measures, time points, analyses), and if not, the methods used to decide which results to collect.	Section 2.2. Supplemental Table 2.
	10b	List and define all other variables for which data were sought (e.g. participant and intervention characteristics, funding sources). Describe any assumptions made about any missing or unclear information.	Section 2.2. Section 2.4. Section 2.5.
Study risk of bias assessment	11	Specify the methods used to assess risk of bias in the included studies, including details of the tool(s) used, how many reviewers assessed each study and whether they worked independently, and if applicable, details of automation tools used in the process.	Section 2.3 Section 2.5
Effect measures	12	Specify for each outcome the effect measure(s) (e.g. risk ratio, mean difference) used in the synthesis or presentation of results.	Section 2.4
Synthesis methods	13a	Describe the processes used to decide which studies were eligible for each synthesis (e.g. tabulating the study intervention characteristics and comparing against the planned groups for each synthesis (item #5)).	Section 2.1.
	13b	Describe any methods required to prepare the data for presentation or synthesis, such as handling of missing summary statistics, or data conversions.	Section 2.5.
	13c	Describe any methods used to tabulate or visually display results of individual studies and syntheses.	Section 2.4. Section 2.5.
	13d	Describe any methods used to synthesize results and provide a rationale for the choice(s). If meta-analysis was performed, describe the model(s), method(s) to identify the presence and extent of statistical heterogeneity, and software package(s) used.	Section 2.4. Section 2.5.
	13e	Describe any methods used to explore possible causes of heterogeneity among study results (e.g. subgroup analysis, meta-regression).	Section 2.5.
	13f	Describe any sensitivity analyses conducted to assess robustness of the synthesized results.	Section 2.5.
Reporting bias assessment	14	Describe any methods used to assess risk of bias due to missing results in a synthesis (arising from reporting biases).	Section 2.5.

Supplemental document 1. PRISMA 2020 Checklist

Certainty assessment	15	Describe any methods used to assess certainty (or confidence) in the body of evidence for an outcome.	Section 2.5.

Section and Topic	Item #	Checklist item	Location where item is reported
**RESULTS**			
Study selection	16a	Describe the results of the search and selection process, from the number of records identified in the search to the number of studies included in the review, ideally using a flow diagram.	Section 3.1. Figure 1.
	16b	Cite studies that might appear to meet the inclusion criteria, but which were excluded, and explain why they were excluded.	Section 3.1. Figure 1.
Study characteristics	17	Cite each included study and present its characteristics.	Supplemental Table 2
Risk of bias in studies	18	Present assessments of risk of bias for each included study.	Supplemental Table 3
Results of individual studies	19	For all outcomes, present, for each study: (a) summary statistics for each group (where appropriate) and (b) an effect estimate and its precision (e.g. confidence/credible interval), ideally using structured tables or plots.	Table 1. Figure 2. Supplemental Table 2 Supplemental Document 1.
Results of syntheses	20a	For each synthesis, briefly summarise the characteristics and risk of bias among contributing studies.	Section 3.2. Section 3.3
	20b	Present results of all statistical syntheses conducted. If meta-analysis was done, present for each the summary estimate and its precision (e.g. confidence/credible interval) and measures of statistical heterogeneity. If comparing groups, describe the direction of the effect.	Section 3.4. Table 1. Figure 2 Supplemental Table 2 Supplemental Document 1. Supplemental Document 2.
	20c	Present results of all investigations of possible causes of heterogeneity among study results.	Section 3.5.
	20d	Present results of all sensitivity analyses conducted to assess the robustness of the synthesized results.	Table 1.
Reporting biases	21	Present assessments of risk of bias due to missing results (arising from reporting biases) for each synthesis assessed.	Not applicable
Certainty of evidence	22	Present assessments of certainty (or confidence) in the body of evidence for each outcome assessed.	Table 1. Figure 2.
**DISCUSSION**			
Discussion	23a	Provide a general interpretation of the results in the context of other evidence.	Section 4.1.
	23b	Discuss any limitations of the evidence included in the review.	Section 4.2.
	23c	Discuss any limitations of the review processes used.	Section 4.2.
	23d	Discuss implications of the results for practice, policy, and future research.	Section 5.
**OTHER INFORMATION**			
Registration and	24a	Provide registration information for the review, including register name and registration number, or state that the review was not registered.	Section 2.1.

protocol	24b	Indicate where the review protocol can be accessed, or state that a protocol was not prepared.	Section 2.1.
	24c	Describe and explain any amendments to information provided at registration or in the protocol.	Not applicable
Support	25	Describe sources of financial or non-financial support for the review, and the role of the funders or sponsors in the review.	Funding Disclosures
Competing interests	26	Declare any competing interests of review authors.	Disclosures
Availability of data, code and other materials	27	Report which of the following are publicly available and where they can be found: template data collection forms; data extracted from included studies; data used for all analyses; analytic code; any other materials used in the review.	Data Availability Statement

From: Page MJ, McKenzie JE, Bossuyt PM, Boutron I, Hoffmann TC, Mulrow CD, et al. The PRISMA 2020 statement: an updated guideline for reporting systematic reviews. BMJ 2021;372:n71. doi: 10.1136/bmj.n71

For more information, visit: http://www.prisma-statement.org/

Supplemental Table 1. Search strategies

Database		Search Strategies	Result
PubMed	#1	Search (("anorexia nervosa"[MeSH Terms]) OR "bulimia nervosa"[MeSH Terms]) OR "binge eating disorder"[MeSH Terms] Sort by: Best Match	14,406
	#2	Search (((((((("stress hormones") OR glucocorticoids[MeSH Terms]) OR cortisol[MeSH Terms]) OR catecholamines[MeSH Terms]) OR vasopressin[MeSH Terms]) OR epinephrine[MeSH Terms]) OR adrenaline[MeSH Terms]) OR norepinephrine[MeSH Terms]) OR noradrenaline[MeSH Terms]) OR dopamine[MeSH Terms] Sort by: Best Match	442,444
	#3	Search (((((((("growth hormone"[MeSH Terms]) OR "reproductive hormones") OR "gonadal hormones"[MeSH Terms]) OR "ovarian hormones") OR estrogens[MeSH Terms]) OR progestogens[MeSH Terms]) OR testosterone[MeSH Terms]) OR prolactin[MeSH Terms]) OR oxytocin[MeSH Terms] Sort by: Best Match	390,108
	#4	Search (((((("appetite hormones") OR "peptide hormones"[MeSH Terms]) OR "gut hormones") OR "gastrointestinal hormones"[MeSH Terms]) OR ghrelin[MeSH Terms]) OR leptin[MeSH Terms]) OR adiponectin[MeSH Terms] Sort by: Best Match	779,256
	#5	Search ((biomarkers[MeSH Terms]) OR hormones[MeSH Terms]) OR peptides[MeSH Terms] Sort by: Best Match	3,916,217
	#6	Search (((((((peripheral[MeSH Terms]) OR blood[MeSH Terms]) OR serum[MeSH Terms]) OR plasma[MeSH Terms]) OR urine[MeSH Terms]) OR saliva[MeSH Terms]) OR "body fluids"[MeSH Terms]) OR hair[MeSH Terms] Sort by: Best Match	1,452,499
	#7	Search #2 OR #3 OR #4 OR #5 OR #6 Sort by: Best Match	5,074,279
	#8	Search #1 AND #7 Sort by: Best Match	2,230
	#9	Search #1 AND #7 Sort by: Best Match<br>Filters: Journal Article; Full text; Humans; English; Adolescent: 13-18 years; Adult: 19+ years	1,100

Supplemental Table 1 (continues)

Database		Search Strategies	Result
PsycINFO	S1	"anorexia nervosa" OR "bulimia nervosa" OR "binge eating disorder" Search modes - Boolean/Phrase	20,715
	S2	"stress hormones" OR glucocorticoids OR cortisol OR catecholamines OR vasopressin OR epinephrine OR adrenaline OR norepinephrine OR noradrenaline OR dopamine Search modes - Boolean/Phrase	92,234
	S3	"growth hormone" OR "reproductive hormones" OR "gonadal hormones" OR "ovarian hormones" OR estrogens OR progestogens OR testosterone OR prolactin OR oxytocin Search modes - Boolean/Phrase	31,563
	S4	"appetite hormones" OR "peptide hormones" OR "gut hormones" OR "gastrointestinal hormones" OR ghrelin OR leptin OR adiponectin Search modes - Boolean/Phrase	4,487
	S5	biomarkers OR hormones OR peptides Search modes - Boolean/Phrase	97,834
	S6	peripheral OR blood OR serum OR plasma OR urine OR saliva OR "body fluids" OR hair Search modes - Boolean/Phrase	201,369
	S7	S2 OR S3 OR S4 OR S5 OR S6 Search modes - Boolean/Phrase	337,969
	S8	S1 AND S7 Search modes - Boolean/Phrase	1,878
	S9	S1 AND S7 Search modes - Boolean/Phrase<br>Limiters - Publication Type: Peer Reviewed Journal; Language: English; Age Groups: Adulthood (18 yrs & older); Population Group: Human; Document Type: Journal Article	940

Supplemental Document 2. Reference list for included studies.

Ainley, C., Cason, J., Slavin, B.M., Wolstencroft, R.A., Thompson, R.P., 1991. The influence of zinc status and malnutrition on immunological function in Crohn's disease. Gastroenterology. 100 (6), 1616-1625.

Amitani, H., Asakawa, A., Ogiso, K., Nakahara, T., Ushikai, M., Haruta, I., Koyama, K., Amitani, M., Cheng, K.C., Inui, A., 2013. The role of adiponectin multimers in anorexia nervosa. Nutrition 29 (1), 203-206.

Arimura, C., Nozaki, T., Takakura, S., Kawai, K., Takii, M., Sudo, N., Kubo, C., 2010. Predictors of menstrual resumption by patients with anorexia nervosa. Eat Weight Disord 15 (4), e226-e233.

Baranowska-Bik, A., Baranowska, B., Martynska, L., Litwiniuk, A., Kalisz, M., Kochanowski, J., Bik, W., 2017. Adipokine profile in patients with anorexia nervosa. Endokrynol Pol 68 (4), 422-429.

Barja-Fernandez, S., Folgueira, C., Seoane, L.M., Casanueva, F.F., Dieguez, C., Castelao, C., Aguera, Z., Banos, R., Botella, C., de la Torre, R., Fernandez-Garcia, J.C., Fernandez-Real, J.M., Fruhbeck, G., Gomez-Ambrosi, J., Jimenez-Murcia, S., Tinahones, F.J., Estivill, X., Fernandez-Aranda, F., Nogueiras, R., 2015. Circulating Betatrophin Levels Are Increased in Anorexia and Decreased in Morbidly Obese Women. J Clin Endocrinol Metab 100 (9), E1188-1196.

Bartak, V., Vybiral, S., Papezova, H., Dostalova, I., Pacak, K., Nedvidkova, J., 2004. Basal and exercise-induced sympathetic nervous activity and lipolysis in adipose tissue of patients with anorexia nervosa. Eur J Clin Invest 34 (5), 371-377.

Baskaran, C., Eddy, K.T., Miller, K.K., Meenaghan, E., Misra, M., Lawson, E.A., 2016. Leptin secretory dynamics and associated disordered eating psychopathology across the weight spectrum. Eur J Endocrinol 174 (4), 503-512.

Bossu, C., Galusca, B., Normand, S., Germain, N., Collet, P., Frere, D., Lang, F., Laville, M., Estour, B., 2007. Energy expenditure adjusted for body composition differentiates constitutional thinness from both normal subjects and anorexia nervosa. Am J Physiol Endocrinol Metab 292 (1), E132-137.

Brambilla, F., Monteleone, P., Bortolotti, F., Dalle Grave, R., Todisco, P., Favaro, A., Santonastaso, P., Ramacciotti, C., Paoli, R., Maj, M., 2003. Persistent amenorrhoea in weight-recovered anorexics: Psychological and biological aspects. Psychiatry Res 118 (3), 249-257.

Brick, D.J., Gerweck, A.V., Meenaghan, E., Lawson, E.A., Misra, M., Fazeli, P., Johnson, W., Klibanski, A., Miller, K.K., 2010. Determinants of IGF1 and GH across the weight spectrum: from anorexia nervosa to obesity. Eur J Endocrinol 163 (2), 185-191.

Broglio, F., Gianotti, L., Destefanis, S., Fassino, S., Abbate Daga, G., Mondelli, V., Lanfranco, F., Gottero, C., Gauna, C., Hofland, L., Van der Lely, A.J., Ghigo, E., 2004. The endocrine response to acute ghrelin administration is blunted in patients with anorexia nervosa, a ghrelin hypersecretory state. Clin Endocrinol (Oxf) 60 (5), 592-599.

Burdo, J., Booij, L., Kahan, E., Thaler, L., Israël, M., Agellon, L.B., Nitschmann, E., Wykes, L., Steiger, H., 2020. Plasma levels of one-carbon metabolism nutrients in women with anorexia nervosa. Int J Eat Disord 53 (9), 1534-1538.

Carlsson, M., Brudin, L., Wanby, P., 2018. Directly measured free 25-hydroxy vitamin D levels show no evidence of vitamin D deficiency in young Swedish women with anorexia nervosa. Eat Weight Disord 23 (2), 247-254.

Casanueva, F.F., Dieguez, C., Popovic, V., Peino, R., Considine, R.V., Caro, J.F., 1997. Serum immunoreactive leptin concentrations in patients with anorexia nervosa before and after partial weight recovery. Biochem Mol Med 60 (2), 116-120.

Case, T., Lemieux, S., Kennedy, S.H., Lewis, G.F., 1999. Elevated plasma lipids in patients with binge eating disorders are found only in those who are anorexic. Int J Eat Disord 25 (2), 187-193.

Cason, J., Ainley, C.C., Wolstencroft, R.A., Norton, K.R., Thompson, R.P., 1986. Cell-mediated immunity in anorexia nervosa. Clin Exp Immunol 64 (2), 370-375.

Casper, R.C., Pandey, G., Jaspan, J.B., Rubenstein, A.H., 1988. Eating attitudes and glucose tolerance in anorexia nervosa patients at 8-year followup compared to control subjects. Psychiatry Res 25 (3), 283-299.

Castillo, M., Scheen, A., Lefebvre, P.J., Luyckx, A.S., 1985. Insulin-stimulated glucose disposal is not increased in anorexia nervosa. J Clin Endocrinol Metab 60 (2), 311-314.

Cinkajzlova, A., Lacinova, Z., Klouckova, J., Kavalkova, P., Trachta, P., Kosak, M., Haluzikova, D., Papezova, H., Mraz, M., Haluzik, M., 2017. Angiopoietin-like protein 6 in patients with obesity, type 2 diabetes mellitus, and anorexia nervosa: The influence of very low-calorie diet, bariatric surgery, and partial realimentation. Endocr Res 42 (1), 22-30.

Connan, F., Lightman, S.L., Landau, S., Wheeler, M., Treasure, J., Campbell, I.C., 2007. An investigation of hypothalamic-pituitary-adrenal axis hyperactivity in anorexia nervosa: The role of CRH and AVP. J Psychiatr Res 41 (1-2), 131-143.

Corcos, M., Guilbaud, O., Chaouat, G., Cayol, V., Speranza, M., Chambry, J., Paterniti, S., Moussa, M., Flament, M., Jeammet, P., 2001. Cytokines and anorexia nervosa. Psychosom Med 63 (3), 502-504.

Counts, D.R., Gwirtsman, H., Carlsson, L.M., Lesem, M., Cutler, G.B., Jr., 1992. The effect of anorexia nervosa and refeeding on growth hormone-binding protein, the insulin-like growth factors (IGFs), and the IGF-binding proteins. J Clin Endocrinol Metab 75 (3), 762-767.

Delporte, M.L., Brichard, S.M., Hermans, M.P., Beguin, C., Lambert, M., 2003. Hyperadiponectinaemia in anorexia nervosa. Clin Endocrinol (Oxf) 58 (1), 22-29.

Dempsey, D.T., Crosby, L.O., Lusk, E., Oberlander, J.L., Pertschuk, M.J., Mullen, J.L., 1984. Total body water and total body potassium in anorexia nervosa. Am J Clin Nutr 40 (2), 260-269.

Djurovic, M., Pekic, S., Petakov, M., Damjanovic, S., Doknic, M., Dieguez, C., Casanueva, F.F., Popovic, V., 2004. Gonadotropin response to clomiphene and plasma leptin levels in weight recovered but amenorrhoeic patients with anorexia nervosa. J Endocrinol Invest 27 (6), 523-527.

Dominguez, J., Goodman, L., Sen Gupta, S., Mayer, L., Etu, S.F., Walsh, B.T., Wang, J., Pierson, R., Warren, M.P., 2007. Treatment of anorexia nervosa is associated with increases in bone mineral density, and recovery is a biphasic process involving both nutrition and return of menses. Am J Clin Nutr 86 (1), 92-99.

Dostalova, I., Bartak, V., Papezova, H., Nedvidkova, J., 2007. The effect of short-term exercise on plasma leptin levels in patients with anorexia nervosa. Metabolism 56 (4), 497-503.

Dostalova, I., Kavalkova, P., Haluzikova, D., Lacinova, Z., Mraz, M., Papezova, H., Haluzik, M., 2008. Plasma concentrations of fibroblast growth factors 19 and 21 in patients with anorexia nervosa. J Clin Endocrinol Metab 93 (9), 3627-3632.

Dostalova, I., Sedlackova, D., Papezova, H., Nedvidkova, J., Haluzik, M., 2009. Serum visfatin levels in patients with anorexia nervosa and bulimia nervosa. Physiol Res 58 (6), 903-907.

Duclos, M., Corcuff, J.B., Roger, P., Tabarin, A., 1999. The dexamethasone-suppressed corticotrophin-releasing hormone stimulation test in anorexia nervosa. Clin Endocrinol (Oxf) 51 (6), 725-731.

Eddy, K.T., Lawson, E.A., Meade, C., Meenaghan, E., Horton, S.E., Misra, M., Klibanski, A., Miller, K.K., 2015. Appetite regulatory hormones in women with anorexia nervosa: Binge-eating/purging versus restricting type. J Clin Psychiatry 76 (1), 19-24.

Estour, B., Marouani, N., Sigaud, T., Lang, F., Fakra, E., Ling, Y., Diamonde, A., Minnion, J.S., Galusca, B., Germain, N., 2017. Differentiating constitutional thinness from anorexia nervosa in DSM 5 era. Psychoneuroendocrinology 84, 94-100.

Fazeli, P.K., Bredella, M.A., Misra, M., Meenaghan, E., Rosen, C.J., Clemmons, D.R., Breggia, A., Miller, K.K., Klibanski, A., 2010. Preadipocyte factor-1 is associated with marrow adiposity and bone mineral density in women with anorexia nervosa. J Clin Endocrinol Metab 95 (1), 407-413.

Fazeli, P.K., Faje, A.T., Cross, E.J., Lee, H., Rosen, C.J., Bouxsein, M.L., Klibanski, A., 2015. Serum FGF-21 levels are associated with worsened radial trabecular bone microarchitecture and decreased radial bone strength in women with anorexia nervosa. Bone 77, 6-11.

Fernandez-Aranda, F., Aguera, Z., Fernandez-Garcia, J.C., Garrido-Sanchez, L., Alcaide-Torres, J., Tinahones, F.J., Giner-Bartolome, C., Banos, R.M., Botella, C., Cebolla, A., de la Torre, R., Fernandez-Real, J.M., Ortega, F.J., Fruhbeck, G., Gomez-Ambrosi, J., Granero, R., Islam, M.A., Jimenez-Murcia, S., Tarrega, S., Menchon, J.M., Fagundo, A.B., Sancho, C., Estivill, X., Treasure, J., Casanueva, F.F., 2016. Smell-taste dysfunctions in extreme weight/eating conditions: analysis of hormonal and psychological interactions. Endocrine 51 (2), 256-267.

Ferrari, E., Fraschini, F., Brambilla, F., 1990. Hormonal circadian rhythms in eating disorders. Biol Psychiatry 27 (9), 1007-1020.

Ferron, F., Considine, R.V., Peino, R., Lado, I.G., Dieguez, C., Casanueva, F.F., 1997. Serum leptin concentrations in patients with anorexia nervosa, bulimia nervosa and non-specific eating disorders correlate with the body mass index but are independent of the respective disease. Clin Endocrinol (Oxf) 46 (3), 289-293.

Flierl, M.A., Gaudiani, J.L., Sabel, A.L., Long, C.S., Stahel, P.F., Mehler, P.S., 2011. Complement C3 serum level in anorexia nervosa: A potential biomarker for the severity of disease? Ann Gen Psychiatry 10 (16), 1-6.

Foppiani, L., Sessarego, P., Valenti, S., Falivene, M.R., Cuttica, C.M., Giusti Disem, M., 1996. Lack of effect of desmopressin on ACTH and cortisol responses to ovine corticotropin-releasing hormone in anorexia nervosa. Eur J Clin Invest 26 (10), 879-883.

Frey, J., Neuhauser-Berthold, M., Elis, S.A., Duncker, S., Rose, F., Blum, W.F., Remschmidt, H., Geller, F., Hebebrand, J., 2003. Lower serum leptin levels in female students of the nutritional sciences with eating disorders. Eur J Nutr 42 (3), 142-148.

Fujimoto, S., Inui, A., Kiyota, N., Seki, W., Koide, K., Takamiya, S., Uemoto, M., Nakajima, Y., Baba, S., Kasuga, M., 1997. Increased cholecystokinin and pancreatic polypeptide responses to a fat-rich meal in patients with restrictive but not bulimic anorexia nervosa. Biol Psychiatry 41 (10), 1068-1070.

Fukuda, I., Hotta, M., Hizuka, N., Takano, K., Ishikawa, Y., Asakawa-Yasumoto, K., Tagami, E., Demura, H., 1999. Decreased serum levels of acid-labile subunit in patients with anorexia nervosa. J Clin Endocrinol Metab 84 (6), 2034-2036.

Fukushima, M., Nakai, Y., Taniguchi, A., Imura, H., Nagata, I., Tokuyama, K., 1993. Insulin sensitivity, insulin secretion, and glucose effectiveness in anorexia nervosa: a minimal model analysis. Metabolism 42 (9), 1164-1168.

Galusca, B., Prevost, G., Germain, N., Dubuc, I., Ling, Y., Anouar, Y., Estour, B., Chartrel, N., 2015. Neuropeptide Y and alpha-MSH circadian levels in two populations with low body weight: anorexia nervosa and constitutional thinness. PLoS ONE 10 (3), e0122040.

Geracioti, T.D., Liddle, R.A., Altemus, M., Demitrack, M.A., Gold, P.W., 1992. Regulation of appetite and cholecystokinin secretion in anorexia nervosa. Am J Psychiatry 149 (7), 958-961.

Germain, N., Galusca, B., Grouselle, D., Frere, D., Tolle, V., Zizzari, P., Lang, F., Epelbaum, J., Estour, B., 2009. Ghrelin/obestatin ratio in two populations with low bodyweight: Constitutional thinness and anorexia nervosa. Psychoneuroendocrinology 34 (3), 413-419.

Germain, N., Galusca, B., Le Roux, C.W., Bossu, C., Ghatei, M.A., Lang, F., Bloom, S.R., Estour, B., 2007. Constitutional thinness and lean anorexia nervosa display opposite concentrations of peptide YY, glucagon-like peptide 1, ghrelin, and leptin. Am J Clin Nutr 85 (4), 967-971.

Germain, N., Viltart, O., Loyens, A., Bruchet, C., Nadin, K., Wolowczuk, I., Estour, B., Galusca, B., 2016. Interleukin-7 plasma levels in human differentiate anorexia nervosa, constitutional thinness and healthy obesity. PLoS ONE 11 (9), e0161890.

Gianotti, L., Fassino, S., Daga, G.A., Lanfranco, F., De Bacco, C., Ramunni, J., Arvat, E., MacCario, M., Ghigo, E., 2000. Effects of free fatty acids and acipimox, a lipolysis inhibitor, on the somatotroph responsiveness to GHRH in anorexia nervosa. Clin Endocrinol (Oxf) 52 (6), 713-720.

Giel, K.E., Kullmann, S., Preißl, H., Bischoff, S.C., Thiel, A., Schmidt, U., Zipfel, S., Teufel, M., 2013. Understanding the reward system functioning in anorexia nervosa: Crucial role of physical activity. Biol Psychol 94 (3), 575-581.

Gill, C.M., Torriani, M., Murphy, R., Harris, T.B., Miller, K.K., Klibanski, A., Bredella, M.A., 2016. Fat attenuation at CT in anorexia nervosa. Radiology 279 (1), 151-157.

Grinspoon, S., Gulick, T., Askari, H., Landt, M., Lee, K., Anderson, E., Ma, Z., Vignati, L., Bowsher, R., Herzog, D., Klibanski, A., 1996. Serum leptin levels in women with anorexia nervosa. J Clin Endocrinol Metab 81 (11), 3861-3863.

Grinspoon, S., Miller, K.K., Herzog, D.B., Grieco, K.A., Klibanski, A., 2004. Effects of estrogen and recombinant human insulin-like growth factor-I on ghrelin secretion in severe undernutrition. J Clin Endocrinol Metab 89 (8), 3988-3993.

Gwirtsman, H.E., Kaye, W.H., George, D.T., Carosella, N.W., Greene, R.C., Jimerson, D.C., 1989. Hyperamylasemia and its relationship to binge-purge episodes: Development of a clinically relevant laboratory test. J Clin Psychiatry 50 (6), 196-204.

Hadigan, C.M., Walsh, B.T., Buttinger, C., Hollander, E., 1995. Behavioral and neuroendocrine responses to metaCPP in anorexia nervosa. Biol Psychiatry 37 (8), 504-511.

Haluzik, M., Kabrt, J., Nedvidkova, J., Svobodova, J., Kotrlikova, E., Papezova, H., 1999. Relationship of serum leptin levels and selected nutritional parameters in patients with protein-caloric malnutrition. Nutrition 15 (11-12), 829-833.

Haluzikova, D., Dostalova, I., Kavalkova, P., Roubicek, T., Mraz, M., Papezova, H., Haluzik, M., 2009. Serum concentrations of adipocyte fatty acid binding protein in patients with anorexia nervosa. Physiol Res 58 (4), 577-581.

Harada, T., Nakahara, T., Yasuhara, D., Kojima, S., Sagiyama, K., Amitani, H., Laviano, A., Naruo, T., Inui, A., 2008. Obestatin, acyl ghrelin, and des-acyl ghrelin responses to an oral glucose tolerance test in the restricting type of anorexia nervosa. Biol Psychiatry 63 (2), 245-247.

Hellzén, M., Larsson, J.O., Reichelt, K.L., Rydelius, P.A., 2003. Urinary peptide levels in women with eating disorders A pilot study. Eat Weight Disord 8 (1), 55-61.

Hildebrandt, M., Rose, M., Mayr, C., Arck, P., Schuler, C., Reutter, W., Salama, A., Klapp, B.F., 2000. Dipeptidyl peptidase IV (DPP IV, CD26) in patients with mental eating disorders. Adv Exp Med Biol 477, 197-204.

Holsen, L.M., Lawson, E.A., Christensen, K., Klibanski, A., Goldstein, J.M., 2014. Abnormal relationships between the neural response to high- and low-calorie foods and endogenous acylated ghrelin in women with active and weight-recovered anorexia nervosa. Psychiatry Res Neuroimaging 223 (2), 94-103.

Innis, S.M., Birmingham, C.L., Harbottle, E.J., 2009. Are plasma homocysteine and methionine elevated when binging and purging behavior complicates anorexia nervosa? Evidence against the transdiagnostic theory of eating disorders. Eat Weight Disord 14 (4), e184-e189.

Jacoangeli, F., Zoli, A., Taranto, A., Mezzasalma, F.S., Ficoneri, C., Pierangeli, S., Menzinger, G., Bollea, M.R., 2002. Osteoporosis and anorexia nervosa: Relative role of endocrine alterations and malnutrition. Eat Weight Disord 7 (3), 190-195.

Johnston, J.L., Leiter, L.A., Burrow, G.N., Garfinkel, P.E., Anderson, G.H., 1984. Excretion of urinary catecholamine metabolites in anorexia nervosa: effect of body composition and energy intake. Am J Clin Nutr 40 (5), 1001-1006.

Kavalkova, P., Dostalova, I., Haluzikova, D., Trachta, P., Hanusova, V., Lacinova, Z., Papezova, H., Domluvilova, D., Zikan, V., Haluzik, M., 2012. Preadipocyte factor-1 concentrations in patients with anorexia nervosa: the influence of partial realimentation. Physiol Res 61 (2), 153-159.

Kaye, W.H., Frank, G.K., McConaha, C., 1999. Altered dopamine activity after recovery from restricting-type anorexia nervosa. Neuropsychopharmacology 21 (4), 503-506.

Kennedy, S.H., Brown, G.M., Ford, C.G., Ralevski, E., 1993. The acute effects of starvation on 6-sulphatoxy-melatonin output in subgroups of patients with anorexia nervosa. Psychoneuroendocrinology 18 (2), 131-139.

Kinzig, K.P., Coughlin, J.W., Redgrave, G.W., Moran, T.H., Guarda, A.S., 2007. Insulin, glucose, and pancreatic polypeptide responses to a test meal in restricting type anorexia nervosa before and after weight restoration. Am J Physiol Endocrinol Metab 292 (5), E1441-1446.

Kršek, M., Rosická, M., Papežová, H., Křížová, J., Kotrlíková, E., Haluz'k, M., Justová, V., Lacinová, Z., Jarkovská, Z., 2003. Plasma ghrelin levels and malnutrition: A comparison of two etiologies. Eat Weight Disord 8 (3), 207-211.
Lanfranco, F., Gianotti, L., Picu, A., Fassino, S., Abbate Daga, G., Mondelli, V., Giordano, R., Grottoli, S., Ghigo, E., Arvat, E., 2004. The adrenal sensitivity to ACTH stimulation is preserved in anorexia nervosa. J Endocrinol Invest 27 (5), 436-441.
Lawson, E.A., Donoho, D., Miller, K.K., Misra, M., Meenaghan, E., Lydecker, J., Wexler, T., Herzog, D.B., Klibanski, A., 2009. Hypercortisolemia is associated with severity of bone loss and depression in hypothalamic amenorrhea and anorexia nervosa. J Clin Endocrinol Metab 94 (12), 4710-4716.
Lawson, E.A., Donoho, D.A., Blum, J.I., Meenaghan, E.M., Misra, M., Herzog, D.B., Sluss, P.M., Miller, K.K., Klibanski, A., 2011. Decreased nocturnal oxytocin levels in anorexia nervosa are associated with low bone mineral density and fat mass. J Clin Psychiatry 72 (11), 1546-1551.
Lawson, E.A., Miller, K.K., Mathur, V.A., Misra, M., Meenaghan, E., Herzog, D.B., Klibanski, A., 2007. Hormonal and nutritional effects on cardiovascular risk markers in young women. J Clin Endocrinol Metab 92 (8), 3089-3094.
Lear, S.A., Pauly, R.P., Birmingham, C.L., 1999. Body fat, caloric intake, and plasma leptin levels in women with anorexia nervosa. Int J Eat Disord 26 (3), 283-288.
Lesem, M.D., George, D.T., Kaye, W.H., Goldstein, D.S., Jimerson, D.C., 1989. State-related changes in norepinephrine regulation in anorexia nervosa. Biol Psychiatry 25 (4), 509-512.
Matsumoto, J., Hirano, Y., Hashimoto, K., Ishima, T., Kanahara, N., Niitsu, T., Shiina, A., Hashimoto, T., Sato, Y., Yokote, K., Murano, S., Kimura, H., Hosoda, Y., Shimizu, E., Iyo, M., Nakazato, M., 2017. Altered serum level of matrix metalloproteinase-9 and its association with decision-making in eating disorders. Psychiatry Clin Neurosci 71 (2), 124-134.
Mayer, L., Walsh, B.T., Pierson, R.N., Jr., Heymsfield, S.B., Gallagher, D., Wang, J., Parides, M.K., Leibel, R.L., Warren, M.P., Killory, E., Glasofer, D., 2005. Body fat redistribution after weight gain in women with anorexia nervosa. Am J Clin Nutr 81 (6), 1286-1291.
Miljic, D., Pekic, S., Djurovic, M., Doknic, M., Milic, N., Casanueva, F.F., Ghatei, M., Popovic, V., 2006. Ghrelin has partial or no effect on appetite, growth hormone, prolactin, and cortisol release in patients with anorexia nervosa. J Clin Endocrinol Metab 91 (4), 1491-1495.
Miller, K.K., Lawson, E.A., Mathur, V., Wexler, T.L., Meenaghan, E., Misra, M., Herzog, D.B., Klibanski, A., 2007. Androgens in women with anorexia nervosa and normal-weight women with hypothalamic amenorrhea. J Clin Endocrinol Metab 92 (4), 1334-1339.
Mondelli, V., Gianotti, L., Picu, A., Abbate Daga, G., Giordano, R., Berardelli, R., Pariante, C.M., Fassino, S., Ghigo, E., Arvat, E., 2006. Neuroendocrine effects of citalopram infusion in anorexia nervosa. Psychoneuroendocrinology 31 (10), 1139-1148.
Monteleone, P., Brambilla, F., Bortolotti, F., Maj, M., 2000a. Serotonergic dysfunction across the eating disorders: relationship to eating behaviour, purging behaviour, nutritional status and general psychopathology. Psychol Med 30 (5), 1099-1110.
Monteleone, P., Di Lieto, A., Tortorella, A., Longobardi, N., Maj, M., 2000b. Circulating leptin in patients with anorexia nervosa, bulimia nervosa or binge-eating disorder: Relationship to body weight, eating patterns, psychopathology and endocrine changes. Psychiatry Res 94 (2), 121-129.
Monteleone, P., Fabrazzo, M., Martiadis, V., Serritella, C., Pannuto, M., Maj, M., 2005. Circulating brain-derived neurotrophic factor is decreased in women with anorexia and bulimia nervosa but not in women with binge-eating disorder: Relationships to co-morbid depression, psychopathology and hormonal variables. Psychol Med 35 (6), 897-905.
Monteleone, P., Maes, M., Fabrazzo, M., Tortorella, A., Lin, A., Bosmans, E., Kenis, G., Maj, M., 1999. Immunoendocrine findings in patients with eating disorders. Neuropsychobiology 40 (3), 115-120.
Monteleone, P., Martiadis, V., Colurcio, B., Maj, M., 2002. Leptin secretion is related to chronicity and severity of the illness in bulimia nervosa. Psychosom Med 64 (6), 874-879.

Monteleone, P., Serritella, C., Martiadis, V., Scognamiglio, P., Maj, M., 2008. Plasma obestatin, ghrelin, and ghrelin/obestatin ratio are increased in underweight patients with anorexia nervosa but not in symptomatic patients with bulimia nervosa. J Clin Endocrinol Metab 93 (11), 4418-4421.
Mori, M., Murakami, M., Satoh, T., Miyashita, K., Iriuchijima, T., Yamada, M., Inukai, T., Kobayashi, I., 1990. A possible direct precursor of thyrotropin-releasing hormone, pGlu-His-Pro-Gly, stimulates prolactin secretion in anorexia nervosa. J Clin Endocrinol Metab 71 (1), 252-255.
Mortola, J.F., Laughlin, G.A., Yen, S.S., 1993. Melatonin rhythms in women with anorexia nervosa and bulimia nervosa. J Clin Endocrinol Metab 77 (6), 1540-1544.
Murialdo, G., Casu, M., Falchero, M., Brugnolo, A., Patrone, V., Cerro, P.F., Ameri, P., Andraghetti, G., Briatore, L., Copello, F., Cordera, R., Rodriguez, G., Ferro, A.M., 2007. Alterations in the autonomic control of heart rate variability in patients with anorexia or bulimia nervosa: correlations between sympathovagal activity, clinical features, and leptin levels. J Endocrinol Invest 30 (5), 356-362.
Nakahara, T., Harada, T., Yasuhara, D., Shimada, N., Amitani, H., Sakoguchi, T., Kamiji, M.M., Asakawa, A., Inui, A., 2008. Plasma obestatin concentrations are negatively correlated with body mass index, insulin resistance index, and plasma leptin concentrations in obesity and anorexia nervosa. Biol Psychiatry 64 (3), 252-255.
Nakahara, T., Kojima, S., Tanaka, M., Yasuhara, D., Harada, T., Sagiyama, K., Muranaga, T., Nagai, N., Nakazato, M., Nozoe, S., Naruo, T., Inui, A., 2007. Incomplete restoration of the secretion of ghrelin and PYY compared to insulin after food ingestion following weight gain in anorexia nervosa. J Psychiatr Res 41 (10), 814-820.
Nakai, Y., Hamagaki, S., Takagi, R., Taniguchi, A., Kurimoto, F., 1999. Plasma concentrations of tumor necrosis factor-alpha (TNF-alpha) and soluble TNF receptors in patients with anorexia nervosa. J Clin Endocrinol Metab 84 (4), 1226-1228.
Nakai, Y., Koh, T., 2001. Perception of hunger to insulin-induced hypoglycemia in anorexia nervosa. Int J Eat Disord 29 (3), 354-357.
Nakazato, M., Tchanturia, K., Schmidt, U., Campbell, I.C., Treasure, J., Collier, D.A., Hashimoto, K., Iyo, M., 2009. Brain-derived neurotrophic factor (BDNF) and set-shifting in currently ill and recovered anorexia nerosa (AN) patients. Psychol Med 39 (6), 1029-1035.
Nedvidkova, J., Dostalova, I., Bartak, V., Papezov, H., Pacak, K., 2004. Increased subcutaneous abdominal tissue norepinephrine levels in patients with anorexia nervosa: an in vivo microdialysis study. Physiol Res 53 (4), 409-413.
Nishita, J.K., Ellinwood, E.H., Jr., Rockwell, W.J., Kuhn, C.M., Hoffman, G.W., Jr., McCall, W.V., Manepalli, J.N., 1989. Abnormalities in the response of plasma arginine vasopressin during hypertonic saline infusion in patients with eating disorders. Biol Psychiatry 26 (1), 73-86.
Nogueira, J.P., Valero, R., Maraninchi, M., Lorec, A.M., Samuelian-Massat, C., Begu-Le Corroller, A., Nicolay, A., Gaudart, J., Portugal, H., Vialettes, B., 2013. Growth hormone level at admission and its evolution during refeeding are predictive of short-term outcome in restrictive anorexia nervosa. Br J Nutr 109 (12), 2175-2181.
Ohwada, R., Hotta, M., Sato, K., Shibasaki, T., Takano, K., 2007. The relationship between serum levels of estradiol and osteoprotegerin in patients with anorexia nervosa. Endocr J 54 (6), 953-959.
Onur, S., Haas, V., Bosy-Westphal, A., Hauer, M., Paul, T., Nutzinger, D., Klein, H., Muller, M.J., 2005. L-tri-iodothyronine is a major determinant of resting energy expenditure in underweight patients with anorexia nervosa and during weight gain. Eur J Endocrinol 152 (2), 179-184.
Otto, B., Cuntz, U., Otto, C., Heldwein, W., Riepl, R.L., Tschop, M.H., 2007. Peptide YY release in anorectic patients after liquid meal. Appetite 48 (3), 301-304.
Pirke, K.-M., Nerl, C., Krieg, J.-C., Fichter, M.M., 1992. Immunological findings in anorexia and bulimia nervosa. Int J Eat Disord 11 (2), 185-189.
Popovic, V., Djurovic, M., Cetkovic, A., Vojvodic, D., Pekic, S., Spremovic, S., Petakov, M., Damjanovic, S., Milic, N., Dieguez, C., Casanueva, F.F., 2004. Inhibin B: a potential marker of gonadal

activity in patients with anorexia nervosa during weight recovery. J Clin Endocrinol Metab 89 (4), 1838-1843.
Rigaud, D., Hassid, J., Meulemans, A., Poupard, A.T., Boulier, A., 2000. A paradoxical increase in resting energy expenditure in malnourished patients near death: the king penguin syndrome. Am J Clin Nutr 72 (2), 355-360.
Rigaud, D., Verges, B., Colas-Linhart, N., Petiet, A., Moukkaddem, M., Van Wymelbeke, V., Brondel, L., 2007. Hormonal and psychological factors linked to the increased thermic effect of food in malnourished fasting anorexia nervosa. J Clin Endocrinol Metab 92 (5), 1623-1629.
Saito, H., Nomura, K., Hotta, M., Takano, K., 2007. Malnutrition induces dissociated changes in lymphocyte count and subset proportion in patients with anorexia nervosa. Int J Eat Disord 40 (6), 575-579.
Schorr, M., Lawson, E.A., Dichtel, L.E., Klibanski, A., Miller, K.K., 2015. Cortisol measures across the weight spectrum. J Clin Endocrinol Metab 100 (9), 3313-3321.
Schorr, M., Marengi, D.A., Pulumo, R.L., Yu, E., Eddy, K.T., Klibanski, A., Miller, K.K., Lawson, E.A., 2017. Oxytocin and its relationship to body composition, bone mineral density, and hip geometry across the weight spectrum. J Clin Endocrinol Metab 102 (8), 2814-2824.
Stengel, A., Hofmann, T., Goebel-Stengel, M., Elbelt, U., Kobelt, P., Klapp, B.F., 2013. Circulating levels of irisin in patients with anorexia nervosa and different stages of obesity--correlation with body mass index. Peptides 39, 125-130.
Stoving, R.K., Andersen, M., Flyvbjerg, A., Frystyk, J., Hangaard, J., Vinten, J., Koldkjaer, O.G., Hagen, C., 2002. Indirect evidence for decreased hypothalamic somatostatinergic tone in anorexia nervosa. Clin Endocrinol (Oxf) 56 (3), 391-396.
Stoving, R.K., Chen, J.W., Glintborg, D., Brixen, K., Flyvbjerg, A., Horder, K., Frystyk, J., 2007. Bioactive insulin-like growth factor (IGF) I and IGF-binding protein-1 in anorexia nervosa. J Clin Endocrinol Metab 92 (6), 2323-2329.
Tagami, T., Satoh, N., Usui, T., Yamada, K., Shimatsu, A., Kuzuya, H., 2004. Adiponectin in anorexia nervosa and bulimia nervosa. J Clin Endocrinol Metab 89 (4), 1833-1837.
Takeno, K., Tamai, H., Matsubayashi, S., Kobayashi, N., Nakagawa, T., Yanaihara, N., 1990. Human pancreatic polypeptide responsiveness to insulin-induced hypoglycemia in anorexia nervosa. Horm Res 33 (5), 190-193.
Tamai, H., Kiyohara, K., Mukuta, T., Kobayashi, N., Komaki, G., Nakagawa, T., Kumagai, L.F., Aoki, T.T., 1991. Responses of growth hormone and cortisol to intravenous glucose loading test in patients with anorexia nervosa. Metabolism 40 (1), 31-34.
Tanaka, M., Nakahara, T., Kojima, S., Nakano, T., Muranaga, T., Nagai, N., Ueno, H., Nakazato, M., Nozoe, S., Naruo, T., 2004. Effect of nutritional rehabilitation on circulating ghrelin and growth hormone levels in patients with anorexia nervosa. Regul Pept 122 (3), 163-168.
Terashi, M., Asakawa, A., Harada, T., Ushikai, M., Coquerel, Q., Sinno, M.H., Dechelotte, P., Inui, A., Fetissov, S.O., 2011. Ghrelin reactive autoantibodies in restrictive anorexia nervosa. Nutrition 27 (4), 407-413.
Terra, X., Auguet, T., Aguera, Z., Quesada, I.M., Orellana-Gavalda, J.M., Aguilar, C., Jimenez-Murcia, S., Berlanga, A., Guiu-Jurado, E., Menchon, J.M., Fernandez-Aranda, F., Richart, C., 2013. Adipocytokine levels in women with anorexia nervosa. Relationship with weight restoration and disease duration. Int J Eat Disord 46 (8), 855-861.
Uehara, M., Yasuhara, D., Nakahara, T., Harada, T., Koyama, K.I., Ushikai, M., Asakawa, A., Inui, A., 2011. Increase in energy intake leads to a decrease in obestatin in restricting-type of anorexia nervosa. Exp Clin Endocrinol Diabetes 119 (9), 536-539.
Urano, A., Hotta, M., Ohwada, R., Araki, M., 2015. Vitamin K deficiency evaluated by serum levels of undercarboxylated osteocalcin in patients with anorexia nervosa with bone loss. Clin Nutr 34 (3), 443-448.

Vaz-Leal, F.J., Ramos-Fuentes, M.I., Guisado-Macías, J.A., Espárrago-Llorca, G., Redondo-Rodríguez, C., Bajo-Cabello, B., Rodríguez-Santos, L., 2020. Coping strategies and stress-induced natural killer cell redistribution in women with eating disorders. Int J Eat Disord 53 (6), 964-971.
Viapiana, O., Gatti, D., Dalle Grave, R., Todesco, T., Rossini, M., Braga, V., Idolazzi, L., Fracassi, E., Adami, S., 2007. Marked increases in bone mineral density and biochemical markers of bone turnover in patients with anorexia nervosa gaining weight. Bone 40 (4), 1073-1077.
Wilhelm, J., Müller, E., de Zwaan, M., Fischer, J., Hillemacher, T., Kornhuber, J., Bleich, S., Frieling, H., 2010. Elevation of homocysteine levels is only partially reversed after therapy in females with eating disorders. J Neural Transm 117 (4), 521-527.
Zumoff, B., Walsh, B.T., Katz, J.L., Levin, J., Rosenfeld, R.S., Kream, J., Weiner, H., 1983. Subnormal plasma dehydroisoandrosterone to cortisol ratio in anorexia nervosa: a second hormonal parameter of ontogenic regression. J Clin Endocrinol Metab 56 (4), 668-672.
Zuniga-Guajardo, S., Garfinkel, P.E., Zinman, B., 1986. Changes in insulin sensitivity and clearance in anorexia nervosa. Metabolism 35 (12), 1096-1100.

Supplemental Table 2. Brief summary of included studies (n = 123)

Author/Year	Country	Study Type	N [a] (% Female)	Age (Year) Mean ± SD or Range	BMI (kg/m^2) Mean ± SD or CI	Duration of Illness (Year)	Biomarker	Biomarker Source	Assay
Zumoff *et al.* 1983	USA	Cross-sectional	AN: 14 Control: 10 (100 %)	19~29	N/A	N/A	Cortisol.	• Blood-plasma	N/A
Dempsey *et al.* 1984	USA	Cross-sectional	AN: 10 Control: 10 (100 %)	26.7 ± 5.06	AN: 14.9 (SD: N/A) Control: 21.4 (SD: N/A)	N/A	Albumin.	• Blood-serum	N/A
Johnston *et al.* 1984	Canada	Cross-sectional	AN: 6 Control: 10 (100 %)	24.33 ± 2.76	AN: 15.8 (SD: N/A) Control: 20.7 (SD: N/A)	N/A	Creatinine.	• Urine	N/A
Castillo *et al.* 1985	Belgium	Longitudinal with intervention†	AN: 6 Control: 13 (100 %)	25.63 ± 5.60	AN: 15.0 (SD: N/A) Control: 21.3 (SD: N/A)	N/A	3-Hydroxybutyrate, fatty acids, glucagon, glucose, glycerol.	• Blood-plasma	Hexokinase oxidase method, enzymatic method.
Cason *et al.* 1986	UK	Cross-sectional	AN: 6 Control: 8 (100 %)	19~55	N/A	N/A	CD3 positive, CD4, CD8.	• Blood-not specify	N/A
Zuniga-Guajardo *et al.* 1986	Canada	Longitudinal with intervention†	AN: 9 Control: 7 (100 %)	24.73 ± 1.84	AN: 16.4 (SD: N/A) Control: 21.3(SD: N/A)	N/A	3-Hydroxybutyrate, c-peptide, glucose, glycerol, immunoreactive insulin.	• Blood-plasma	Glucose oxidase method, enzymatic method.
Casper *et al.* 1988	USA	Cross-sectional	AN: 7 Control: 14 (100%)	24.03 ± 2.71	N/A	N/A	Cortisol, potassium.	• Blood-serum	RIA.
Gwirtsman *et al.* 1989	USA	Cross-sectional	AN: 45 Control: 31 (%)	22.78 ± 0.55	N/A	5.78	Na, potassium.	• Blood-serum	N/A
Lesem *et al.* 1989	USA	Longitudinal with intervention†	AN: 11 Control: 9 (100 %)	26.28 ± 4.60	N/A	N/A	Na.	• Blood-plasma	N/A
Nishita *et al.* 1989	USA	Cross-sectional	AN: 29 Control: 28 (100 %)	24.55 ± 5.51	AN: 16.1 (SD: N/A) Control:21.7 (SD: N/A)	4.48	ACTH, cortisol, Na.	• Blood-plasma	RIA.
Ferrari *et al.* 1990	Italy	Longitudinal without intervention†	AN: 23 Control: 43 (100 %)	27.96 ± 7.90	AN: 14.8 ± 0.39 Control: 38.7 ±1.5	2.51	Cortisol, GH, melatonin (N-acetyl-5 methoxytryptamine), Prolactin.	• Blood-plasma	N/A

Supplemental Table 2 (continues)

Author/Year	Country	Study Type	N [a] (% Female)	Age (Year) Mean ± SD or Range	BMI (kg/m^2) Mean ± SD or CI	Duration of Illness (Year)	Biomarker	Biomarker Source	Assay
Kaye *et al.* 1990	USA	Cross-sectional	AN: 15 Control: 10 (100 %)	24.94 ± 3.98	N/A	7.08	Peptide tyrosine tyrosine.	• Blood-plasma	RIA.
Mori *et al.* 1990	Japan	Longitudinal with intervention†	AN: 9 Control: 6 (100 %)	23.04 ± 1.58	N/A	N/A	Prolactin, thyroid stimulating hormone.	• Blood-not specify	RIA.
Takeno *et al.* 1990	Japan	Longitudinal with intervention†	AN: 10 Control: 8 (100 %)	20.79 ± 1.51	AN: 13.8 ± 0.5 Control: 20.6 ± 0.2	N/A	Glucagon, pancreatic polypeptide.	• Blood-plasma	RIA.
Ainley *et al.* 1991	UK	Cross-sectional	AN: 12 Control: 18 (100 %)	18~75	N/A	N/A	Albumin, CD3 positive, CD4, CD8, lymphocyte.	• Blood-plasma	N/A
Tamia *et al.* 1991	Japan	Longitudinal with intervention†	AN: 19 Control: 8 (100 %)	19.98 ± 1.05	N/A	N/A	Glucose, immunoreactive insulin, potassium.	• Blood-serum	Glucose dehydrogenase method, RIA.
Counts *et al.* 1992	USA	Cross-sectional	AN: 11 Control: 6 (100 %)	24.65 (18~35)	AN: 12.2 (SD: N/A) Control:22.4 (SD: N/A)	5.9	GH.	• Blood-serum	Ligand-mediated immunofunctional assay
Geracioti *et al.* 1992	USA	Longitudinal with intervention†	AN: 6 Control: 6 (100 %)	24.00 ± 4.32	N/A	6	Cholecystokinin.	• Blood-plasma	N/A
Pirke *et al.* 1992	Germany	Longitudinal with intervention†	AN: 10 Control: 9 (100 %)	24.35 ± 3.41	AN: 14.8 ± 1.2 Control:21.0 ± 2.3	5.03	CD3 positive, CD4, CD8, lymphocyte.	• Blood-not specify	Fluoroimmunoassay
Fukushima *et al.* 1993	Japan	Longitudinal with intervention†	AN: 8 Control: 16 (100 %)	22.8 ± 2.14	AN: 14.7 ± 0.2 Control:20.6 ± 0.5	4.66	Free thyroxine, glucose, insulin, thyrotropin.	• Blood-plasma	Glucose oxidase method, RIA, CLIA.
Kennedy *et al.* 1993	Canada	Cross-sectional	AN: 10 Control: 13 (100 %)	25.55 ± 5.39	AN: 15 ± 2.0 Control:23.7 ± 3.2	8.2	Melatonin (N-acetyl-5 methoxytryptamine).	• Urine	RIA.
Mortola *et al.* 1993	USA	Cross-sectional	AN: 8 Control: 21 (100 %)	29.42 ± 2.22	AN: 15.3 (SD: N/A) Control:20.3 (SD: N/A)	6.3	Melatonin (N-acetyl-5 methoxytryptamine).	• Blood-serum	N/A

Supplemental Table 2 (continues)

Author/Year	Country	Study Type	N [a] (% Female)	Age (Year) Mean ± SD or Range	BMI (kg/m^2) Mean ± SD or CI	Duration of Illness (Year)	Biomarker	Biomarker Source	Assay
Hadigan *et al.* 1995	USA	Longitudinal with intervention[†]	AN: 10<br>Control: 8<br>(100 %)	25.4 ± 5.15	AN: 14.6 ± 1.5<br>Control:21.2 ± 2.0	5.4	Cortisol, prolactin.	• Blood-plasma	RIA.
Foppiani *et al.* 1996	Italy	Longitudinal with intervention[†]	AN: 8<br>Control: 6<br>(100 %)	23.03 ± 3.37	AN: 16.3 ± 2.2<br>Control:21 ± 3.3	6	Estradiol, IGF-1.	• Blood-serum	RIA.
Grindpoon *et al.* 1996	USA	Cross-sectional	AN: 22<br>Control: 23<br>(100 %)	23 ± 3.95	AN: 16.3 ± 1.6<br>Control:23.7 ± 1.7	5	IGF-1, leptin.	• Blood-serum	RIA.
Case *et al.* 1997	Canada	Cross-sectional	AN: 9<br>Control: 10<br>(100 %)	29.94 ± 5.68	AN: 17.4 ± 0.7<br>Control: 22.9 ± 1.2	8.4	Cholesterol, triglycerides.	• Blood-plasma	N/A
Cassanueva *et al.* 1997	USA	Longitudinal with intervention[†]	AN: 10<br>Control: 18<br>(100 %)	22.78 ± 2.38	AN: 15.3 ± 0.6<br>Control: 21.1 ± 0.3	N/A	Insulin-like growth factor 1, IGFBP−3, leptin.	• Blood-serum	RIA.
Ferron *et al.* 1997	Spain	Cross-sectional	AN: 25<br>Control: 14<br>(100 %)	21.98 ± 1.39	AN: 18.8 ± 0.2<br>Control:21.4 ± 0.4	N/A	Leptin.	• Blood-serum	RIA.
Fujimoto *et al.* 1997	Japan	Cross-sectional	AN: 7<br>Control: 8<br>(100 %)	21.1 ± 1.93	AN: 15.1 ± 0.8<br>Control:21.1 ±1.3	3.4	Cholecystokinin.	• Blood-plasma	RIA.
Duclos *et al.* 1999	France	Cross-sectional	AN: 19<br>Control: 6<br>(100 %)	24.92 ± 2.54	AN: 15.3 ± 0.6<br>Control:22.9 ± 2.4	N/A	Cortisol, creatinine.	• Urine	RIA.
Fukuda *et al.* 1999	Japan	Cross-sectional	AN: 33<br>Control: 34<br>(100 %)	24.02 ± 3.05	AN: 16 ± 0.4<br>Control: N/A	N/A	IGFBP-2.	• Blood-serum	RIA.
Haluzi´k *et al.* 1999	Czech Republic	Cross-sectional	AN: 17<br>Control: 17<br>(100 %)	37.11 ± 14.83	AN: 15.22 ± 3.06<br>Control:24.91 ± 16.4	N/A	Albumin, cholesterol, Leptin, total protein, triglycerides.	• Blood-serum	RIA.
Lear *et al.* 1999	Canada	Cross-sectional	AN: 15<br>Control: 15<br>(100 %)	28.57 ± 2.16	AN: 16.8 ± 0.7<br>Control:21.6 ± 0.4	N/A	Leptin.	• Blood-plasma	RIA.

Supplemental Table 2 (continues)

Author/Year	Country	Study Type	N [a] (% Female)	Age (Year) Mean ± SD or Range	BMI (kg/m^2) Mean ± SD or CI	Duration of Illness (Year)	Biomarker	Biomarker Source	Assay
Monteleone *et al.* 1999	Belgium	Cross-sectional	AN:21<br>Control:21<br>(100 %)	23.65 ± 5.17	AN:16.9 ± 2.0<br>Control:21.9 ± 2.1	5.4	Estradiol.	• Blood-plasma	IRMA.
Nakai *et al.* 1999	Japan	Cross-sectional	AN: 20<br>Control: 20<br>(100 %)	21.15 ± 1.21	AN: 13.7 ± 0.4<br>Control:19.9 ± 0.2	3.75	Leptin,<br>tumor necrosis factor-α.	• Blood-plasma	RIA.
Gianotto *et al.* 2000	Italy	Longitudinal with intervention[†]	AN: 9<br>Control: 8<br>(100 %)	26.02 ± 2.55	AN: 14.1 ± 0.6<br>Control: 20.1 ± 0.5	4.4	IGF-1, IGFBP-1, IGFBP-3, insulin.	• Blood-serum	RIA, IRMA.
Hildebrandt *et al.* 2000	Germany	Cross-sectional	AN: 21<br>Control: 20<br>(100 %)	N/A	AN: 14.6 ± 1.8<br>Control: 21.9 ± 2.3	5	CD3 positive, lymphocyte.	• Blood-serum	Automated analyzer, flow cytometry
Monteleone *et al.* 2000a	Italy	Cross-sectional	AN: 15<br>Control: 15<br>(100%)	25.40 ± 5.55	AN: 16.4 ± 1.2<br>Control: 21.5 ± 2.7	6.7	Testosterone.	• Blood-plasma	IRMA
Monteleone et al. 2000b	Italy	Cross-sectional	AN: 21<br>Control: 15<br>(100 %)	25.22 ± 5.10	AN: 16.0 ± 1.6<br>Control:21.7 ± 2.2	5.3	Glucose.	• Blood-plasma	IRMA, enzymatic method.
Rigaud *et al.* 2000	France	Cross-sectional	AN: 16<br>Control: 16<br>(100 %)	24.5 ± 4.00	AN: 13.6 ± 1.1<br>Control: 21.2 ± 1.7		Albumin, calcium, creatinine, fatty acids, free thyroxine, free triiodothyronine, glucose Insulin, Na, norepinephrine, potassium, zinc, thyrotropin, transferrin, phosphorus.	• Blood-serum,<br>• Urine	RIA, high-performance liquid chromatography, gas chromatography, laser nephelometry, automated analyzer.
Corcos *et al.* 2001	France	Cross-sectional	AN: 29<br>Control: 20<br>(100 %)	21.57 ± 2.86	AN: N/A<br>Control: N/A	N/A	Tumor necrosis factor-α.	• Blood-serum	ELISA.
Nakai *et al.* 2001	Japan	Longitudinal with intervention[†]	AN: 20<br>Control: 20<br>(100 %)	18~29	AN: 15 ± 0.5<br>Control: 19.8 ± 0.5		Glucose.	• Blood-not specify	Ferricyanide reduction method.

Supplemental Table 2 (continues)

Author/Year	Country	Study Type	N [a] (% Female)	Age (Year) Mean ± SD or Range	BMI (kg/m^2) Mean ± SD or CI	Duration of Illness (Year)	Biomarker	Biomarker Source	Assay
Jacoangeli *et al.* 2002	Italy	Cross-sectional	AN: 49 Control: 24 (100 %)	23.57 ± 5.43	AN: 16.7 ± 2.92 Control: 22.58 ± 4.12	4.8	25-OH-vitamin D, calcium, cortisol, estradiol, Free thyroxine, free triiodothyronine, IGF-1, osteocalcin, parathyroid hormone, thyroid stimulating hormone.	• Blood-not specify	N/A
Monteleone *et al.* 2002	Belgium	Cross-sectional	AN:33 Control:38 (100 %)	23.98 ± 5.11	AN:15.7 ± 1.6 Control:21.7 ± 2.1	4.3	Cortisol, leptin, prolactin.	• Blood-plasma	RIA, ELISA, IRMA.
Støving et al. 2002	Denmark	Longitudinal without intervention[†]	AN: 13 Control: 10 (100 %)	24.46 ± 2.13	AN: 15 ± 0.4 Control:20.6 ± 0.5	4.7	Cortisol, IGFBP-3, insulin, leptin.	• Blood-serum	RIA
Brambilla *et al.* 2003	Italy	Cross-sectional	AN: 22 Control: 20 (100 %)	23.35 ± 4.29	AN: 19.3 ± 1.1 Control:20.4 ± 1.2	4.7	Cortisol, estradiol, follicle stimulating hormone, Free thyroxine, free triiodithyronine, leptin, prolactin, thyroid stimulating hormone.	• Blood-plasma	IRMA, ELISA
Delporte *et al.* 2003	Belgium	Cross-sectional	AN: 26 Control: 24 (100 %)	21.98 ± 1.59	AN: 14.3 ± 0.4 Control:22.4 ± 0.7	N/A	Adiponectin, cholesterol, C-peptide, glucose, HDL, insulin, LDL, leptin, triglycerides.	• Blood-plasma	RIA, glucose oxidase method, enzymatic method.
Frey *et al.* 2 003	Germany	Cross-sectional	AN: 16 Control: 32 (100 %)	23.6 ± 2.93	AN: 19.87 ± 2.44 Control:19.95 ± 2.40	N/A	Leptin.	• Blood-serum	RIA
Hellzén *et al.* 2003	Sweden	Cross-sectional	AN: 10 Control: 10 (100 %)	25.40 ± 5.10	AN: 19.0 ± 2.4 Control: 20.8 ± 1.4	9.5	Na, potassium.	• Blood-not specify	N/A
Krsek *et al.* 2003	Czech Republic	Cross-sectional	AN: 16 Control: 13 (100 %)	27.77 ± 7.58	AN: 15.2 ± 2.8 Control:20.7 ± 2.0		Albumin, ghrelin, GH, transferrin, IGF-1, IGFBP-1, IGFBP-3, leptin, soluble leptin receptor, total protein.	• Blood-serum • Blood-plasma	RIA, EIA

Supplemental Table 2 (continues)

Author/Year	Country	Study Type	N [a] (% Female)	Age (Year) Mean ± SD or Range	BMI (kg/m^2) Mean ± SD or CI	Duration of Illness (Year)	Biomarker	Biomarker Source	Assay
Bartak *et al.* 2004	USA	Longitudinal with intervention[†]	AN: 10 Control: 10 (100 %)	22.65 ± 1.13	AN: 15.57 ± 0.55 Control:21.56 ± 0.41	N/A	Glycerol, norepinephrine.	• Blood-plasma	High-performance liquid chromatography, IRMA.
Broglio *et al.* 2004	Italy	Longitudinal with intervention[†]	AN: 9 Control: 7 (100 %)	27.00 ± 4.04	AN: 14.7 ± 0.4 Control:20.3 ± 0.5	N/A	ACTH, cortisol, GH, ghrelin, glucose, IGF−1, insulin, prolactin.	• Blood-serum, Blood-plasma	IRMA.
Djurovic *et al.* 2004	Spain	Longitudinal with intervention[†]	AN: 17 Control: 10 (100 %)	23.47 ± 5.17	AN: 14.9 ± 0.5 Control: 21.1 ± 0.3	N/A	Leptin.	• Blood-plasma	RIA.
Grinspoon *et al.* 2004	USA	Longitudinal with intervention[†]	AN: 58 Control: 20 (100 %)	24.91 ± 1.85	AN: 17.04 ± 0.41 Control: 15.9 ± 0.4	N/A	GH, ghrelin, IGF-1.	• Blood-serum	RIA.
Laanfranco *et al.* 2004	Italy	Longitudinal with intervention[†]	AN:10 Control:10 (100%)	22.3 ± 1.49	AN: 15.7 ± 0.6 Control:21.9 ± 0.9	N/A	Cortisol, dehydroepiandrosterone.	• Blood-serum	RIA.
Nedvidkova *et al.* 2004	Czech Republic	Longitudinal with intervention[†]	AN: 5 Control: 6 (100 %)	23.25 ± 1.69	AN: 14.6 ± 0.5 Control:22.1 ± 0.5	N/A	Glycerol, norepinephrine.	• Blood-not specify	High-performance liquid chromatography.
Popovic *et al.* 2004	Spain	Cross-sectional	AN: 20 Control: 19 (100 %)	21.97 ± 3.08	AN: 14.3 ± 0.3 Control:19.8 ± 0.5	N/A	Follicle stimulating hormone, leptin, luteinizing hormone.	• Blood-serum	RIA, IRMA.
Tagami *et al.* 2004	Japan	Longitudinal without intervention[†]	AN: 31 Control: 31 (100 %)	25.96 ± 6.51	AN: 14.0 ± 2.5 Control:27.55 ± 12.41	N/A	Adiponectin, insulin, leptin.	• Blood-serum, Blood-plasma	RIA.
Tanaka *et al.* 2004	Japan	Longitudinal without intervention[†]	AN: 34 Control: 9 (100 %)	21.00 ± 3.17	AN: 15.27 ± 0.92 Control: 21.5 ± 0.4	2-5.2	Alanine aminotransferase, Aspartate aminotransferase, cholesterol, GH, ghrelin, glucose.	• Blood-serum, Blood-plasma	RIA.
Mayer *et al.* 2005	USA	Longitudinal with intervention[†]	AN:29 Control:15 (100 %)	24.69 ± 5.60	AN:15.95 ± 1.61 Control:20.61 ± 0.90	7.38	Cortisol, estradiol, testosterone.	• Blood-serum	CLIA.
Monteleone *et al.* 2005	Italy	Cross-sectional	AN: 27 Control: 24 (100 %)	21.13 ± 4.57	AN: 15.9 ± 2.4 Control:21.8 ± 1.8	3	Free thyroxine, free triiodothyronine.	• Blood-serum	ELISA.

Supplemental Table 2 (continues)

Author/Year	Country	Study Type	N [a] (% Female)	Age (Year) Mean ± SD or Range	BMI (kg/m^2) Mean ± SD or CI	Duration of Illness (Year)	Biomarker	Biomarker Source	Assay
Onur *et al.* 2005	Germany	Longitudinal with intervention†	AN: 28 Control: 49 (100 %)	25.00 ± 4.81	AN: 15.1 ± 1.5 Control: 22.3 ± 2.3	N/A	Free thyroxine, free triiodothyronine, thyroid stimulating hormone.	Blood-plasma	CLIA.
Miljic *et al.* 2006	UK	Cross-sectional	AN: 9 Control: 10 (100 %)	23.71 ± 2.01	AN: 12 ± 0.4 Control: 17.6 ± 0.4	N/A	Adiponectin, Cortisol, C-peptide, GH, ghrelin, glucose, insulin, leptin, prolactin.	• Blood-plasma	RIA, IRMA.
Mondelli *et al.* 2006	Italy	Cross-sectional	AN:6 Control:6 (100%)	29.45 ± 3.13	AN: 15.7 ± 0.5 Control:21.4 ± 1.0	N/A	ACTH, cortisol, GH, prolactin.	• Blood-serum	IRMA, RIA.
Bossu *et al.* 2007	France	Cross-sectional	AN: 6 Control: 14 (100 %)	18~26	AN: 15.8 ± 0.8 Control: 18.65 ± 2.78	N/A	Free triiodithyronine, IGF−1, Leptin, free triiodithyronine	• Blood-plasma	IRMA, RIA.
Connan *et al.* 2007	UK	Longitudinal with intervention†	AN: 18 Control: 15 (100 %)	26.92 ± 5.57	AN: 14.6 ± 1.0 Control: 21.8 ± 2.0	N/A	ACTH, cortisol.	• Blood-serum, • Blood-plasma	EIA, CLIA.
Dominguez *et al.* 2007	USA	Longitudinal with intervention†	AN:28 Control:11 (100 %)	23.48 ± 4.27	AN:16.1 ± 1.7 Control:21.5 ± 0.9	8.17	Estradiol, follicle stimulating hormone, luteinizing hormone, osteocalcin, testosterone.	• Blood-serum	CLIA.
Dostalova *et al.* 2007	Czech Republic	Longitudinal without intervention†	AN: 10 Control: 15 (100 %)	21.62 ± 1.00	AN: 15.7 ± 0.47 Control: 21.2 ± 0.42	N/A	Norepinephrine.	• Blood-plasma	RIA, IRMA, high-performance liquid chromatography.
Germain *et al.* 2007	USA	Cross-sectional	AN: 12 Control: 17 (100 %)	21.08 ± 1.56	AN: 15.2 ± 0.4 Control: 17.64 ± 2.40		Cortisol, estradiol, follicle stimulating hormone, free triiodothyronine, ghrelin, IGF-1, luteinizing hormone, leptin, peptide tyrosine tyrosine.	• Blood-serum, • Blood-plasma	RIA, IRMA.
Kinzig *et al.* 2007	USA	Longitudinal with intervention†	AN: 13 Control: 13 (92.31 %)	27.3 ± 2.41	AN: 16.8 ± 0.3 Control:23.8 ± 0.6	N/A	Glucose, insulin, pancreatic polypeptide.	• Blood-serum	Enzymatic method, Fluoroimmunoassay, RIA.
Lawson *et al.* 2007	USA	Cross-sectional	AN: 140 Control: 41 (100 %)	25.39 ± 2.04	AN: 17.10 ± 0.32 Control: 21.29 ± 0.59	N/A	HDL, IGF-1, LDL.	• Blood-serum	Automated analyzer, IRMA.

Supplemental Table 2 (continues)

Author/Year	Country	Study Type	N [a] (% Female)	Age (Year) Mean ± SD or Range	BMI (kg/m^2) Mean ± SD or CI	Duration of Illness (Year)	Biomarker	Biomarker Source	Assay
Miller *et al.* 2007	USA	Cross-sectional	AN: 169 Control: 27 (100 %)	25.10 ± 0.82	AN: 16.78 ± 0.20 Control:21 ± 0.3	N/A	Dehydroepiandrosterone sulfate, sex hormone–binding globulin, testosterone.	• Blood-serum	RIA, IRMA.
Murialdo *et al.* 2007	Italy	Cross-sectional	AN: 34 Control: 30 (100 %)	25.05 (SD: N/A)	AN: 15.7 (CI: 15.1,16.4) Control: 22.5 (CI: 22.3, 23.6)	4.61	Leptin.	• Blood-not specify	RIA.
Nakahara *et al.* 2007	Japan	Longitudinal with intervention[†]	AN: 14 Control: 12 (100 %)	30.49 ± 7.68	AN: 12.4 ± 1.7 Control: 22.3 ± 2.2	N/A	Free triiodithyronine, ghrelin, glucose, insulin, leptin, peptide tyrosine tyrosine, total protein.	• Blood-serum, Blood-plasma	RIA, glucose oxidase method, EIA, CLIA.
Ohwada *et al.* 2007	Japan	Cross-sectional	AN: 26 Control: 7 (100 %)	23.50 ± 0.98	AN: 13.8 ± 0.6 Control: 22.1 ± 0.5		Bone alkaline phosphatase, calcium, carboxy-terminal collagen crosslinks, estradiol Intact parathyroid hormone, IGF-1, leptin.	• Blood-serum	CLIA, IRMA, RIA.
Otto *et al.* 2007	Germany	Longitudinal with intervention[†]	AN: 16 Control: 7 (100 %)	25.91 ± 2.30	AN: 15.2 ± 0.3 Control:21.2 ± 0.6	N/A	Peptide tyrosine tyrosine.	• Blood-plasma	ELISA.
Rigaud *et al.* 2007	France	Cross-sectional	AN: 15 Control: 15 (100 %)	23.50 ± 3.51	AN: 13.6 ± 1.2 Control:21.6 ± 1.9	N/A	Insulin.	• Blood-plasma	RIA.
Saito *et al.* 2007	Japan	Cross-sectional	AN: 33 Control: 10 (100 %)	23.79 ± 1.37	AN: 13 ± 0.4 Control:19.3 ± 1.2	N/A	CD4, CD8, IGF-1, zinc.	• Blood-not specify	IRMA, atomic absorption spectrophotometry, automated analyzer, fluoroimmunoassay.
Støving *et al.* 2007	Denmark	Longitudinal without intervention[†]	AN: 27 Control: 24 (100 %)	28.50 ± 7.04	AN: 15.22 ± 1.96 Control:23.6 ± 3.7	N/A	Adiponectin, estradiol, follicle stimulating hormone, ghrelin, glucose, insulin, luteinizing hormone.	• Blood-serum, Blood-plasma	RIA, fluorimetric method, glucose dehydrogenase method.
Viapiana *et al.* 2007	Italy	Longitudinal without intervention[†]	AN: 55 Control: 15 (100 %)	25.07 ± 6.77	AN: 14.39 ± 1.69 Control: 22.64 ± 2.74	8.06	25-OH-vitamin D, bone alkaline phosphatase, carboxy-terminal collagen crosslinks, osteocalcin.	• Blood-serum	ELISA, standard laboratory methods.

Supplemental Table 2 (continues)

Author/Year	Country	Study Type	N [a] (% Female)	Age (Year) Mean ± SD or Range	BMI (kg/m²) Mean ± SD or CI	Duration of Illness (Year)	Biomarker	Biomarker Source	Assay
Dostalova *et al.* 2008	Czech Republic	Longitudinal without intervention†	AN: 17 Control: 17 (100 %)	24.85 ± 1.03	AN: 15.9 ± 0.33 Control: 22.9 ± 0.41	N/A	Adiponectin, CRP, fatty acids, free thyroxine, free triiodithyronine, glucose, glycerol, IGF−1, insulin, leptin, resistin, soluble leptin receptor, thyroid stimulating hormone, visfatin.	Blood-plasma	ELISA, RIA, standard laboratory methods.
Harada *et al.* 2008	Japan	Longitudinal with intervention†	AN: 10 Control: 10 (100 %)	22.70 ± 1.57	AN: 13.43 ± 0.29 Control: 21.60 ± 1.15	N/A	Acylated ghrelin, des-acyl ghrelin, leptin, obestatin.	• Blood-plasma	RIA, ELISA
Monteleone *et al.* 2008	Italy	Cross-sectional	AN: 20 Control: 20 (100 %)	23.50 ±6.49	AN: 16.6 ±1.6 Control: 21.1 ± 2.2	N/A	Ghrelin, obestatin.	• Blood-plasma	ELISA.
Nakahara *et al.* 2008	Japan	Cross-sectional	AN: 11 Control: 21 (100 %)	25.95 ± 6.88	AN: 12.4 ± 1.7 Control: 24.94 ± 4.42	N/A	Acylated ghrelin, adiponectin, des-acyl ghrelin, insulin, leptin, obestatin.	• Blood-serum, Blood-plasma	RIA, ELISA, glucose oxidase method, glucose oxidase method.
Dostalova *et al.* 2009	Czech Republic	Cross-sectional	AN: 10 Control: 20 (100 %)	22.80 ± 0.82	AN: 14.5 ± 0.46 Control:21.8 ± 0.36	N/A	Glucose, insulin, leptin, visfatin.	• Blood-plasma	RIA, EIA.
Germain *et al.* 2009	France	Cross-sectional	AN: 15 Control: 19 (100 %)	22.17 ± 2.07	AN: 14.8 ± 0.1 Control: 18.42 ± 2.28	N/A	Acylated ghrelin, GH, ghrelin, IGF-1, leptin, obestatin, sex hormone–binding globulin.	• Blood-plasma	RIA, IRMA, EIA.
Haluzikova *et al.* 2009	Czech Republic	Cross-sectional	AN: 19 Control: 16 (100 %)	24.86 ± 1.06	AN: 15.9 ± 0.33 Control: 22.9 ± 0.41	N/A	Adiponectin, cholesterol, CRP, glucose, insulin, leptin, resistin, soluble leptin receptor, triglycerides.	• Blood-serum	ELISA.
Innis *et al.* 2009	Canada	Cross-sectional	AN: 62 Control: 82 (71.79 %)	27.08 ± 0.98	AN: 18.65 ± 0.61 Control:19.3 ± 0.38	N/A	Homocysteine.	• Blood-plasma	High-performance liquid chromatography
Lawson *et al.* 2009	USA	Cross-sectional	AN: 18 Control: 34 (100 %)	26.60 ± 1.67	AN: 18.2 ±0.2 Control:21.99 ± 0.88	N/A	Cortisol, IGF-1, leptin.	• Blood-serum	CLIA, RIA.

Supplemental Table 2 (continues)

Author/Year	Country	Study Type	N [a] (% Female)	Age (Year) Mean ± SD or Range	BMI (kg/m^2) Mean ± SD or CI	Duration of Illness (Year)	Biomarker	Biomarker Source	Assay
Nakazato *et al* 2009	UK	Cross-sectional	AN: 29<br>Control: 28<br>(100 %)	27.61 ± 8.79	AN: 15.6 ± 1.6<br>Control: 22.3 ± 2.5	10.6	Brain-derived neurotrophic factor.	Blood-serum	EIA.
Arimura *et al.* 2010	Japan	Longitudinal with intervention[†]	AN: 20<br>Control: 12<br>(100 %)	20.75 ± 5.41	AN: 13.27 ± 1.48<br>Control: 21.1 ± 2.1	2.99	Cortisol, estradiol, follicle stimulating hormone, free thyroxine, free triiodithyronine, GH, IGF−1, leptin, luteinizing hormone, thyroid stimulating hormone, prolactin	Blood-not specify	ELISA, RIA, EIA, CLIA.
Brick *et al.* 2010	USA	Cross-sectional	AN:11<br>Control: 21<br>(100 %)	31.79 ± 2.38	AN: 16.6 ± 0.5<br>Control: 28.44 ± 6.64	N/A	Estradiol, fatty acids, insulin, sex hormone–binding globulin, testosterone, thyroid stimulating hormone, thyroid stimulating hormone.	• Blood-serum	CLIA.
Fazeli *et al.* 2010	USA	Cross-sectional	AN: 20<br>Control: 10<br>(100 %)	27.60 ± 1.92	AN: 17.6 ± 0.2<br>Control: 21.9 ± 0.5	N/A	IGF-1, IGFBP-2, leptin, preadipocyte factor-1.	• Blood-not specify	RIA.
Wilhelm *et al.* 2010	Germany	Cross-sectional	AN: 12<br>Control: 20<br>(100 %)	22.86 ± 5.87	AN: 14.31 ± 2.19<br>Control: 21.65 ±1.82	N/A	Homocysteine, vitamin B12.	• Blood-plasma	High-performance liquid chromatography.
Flierl *et al.* 2011	USA	Cross-sectional	AN: 14<br>Control: 17<br>(80.6 %)	30.30 ± 8.91	AN: 13.6 ± 1.5<br>Control: 22.2 ± 2.6	N/A	Total protein.	• Blood-serum	Bicinchoninic acid assay.
Lawson *et al.* 2011	USA	Cross-sectional	AN: 17<br>Control: 19<br>(100 %)	27.59 ± 1.82	AN: 18.1 ± 0.2<br>Control: 24.1 ± 0.5	N/A	Estradiol, leptin, oxytocin.	• Blood-serum	RIA.
Terashi *et al.* 2011	Japan	Longitudinal without intervention[†]	AN: 10<br>Control: 10<br>(100 %)	22.70 ± 1.58	AN: 13.6 ± 0.6<br>Control: 21.6 1.2	N/A	Acylated ghrelin, des-acyl ghrelin.	• Blood-plasma	ELISA.
Uehara *et al.* 2011	Japan	Cross-sectional	AN: 9<br>Control: 9<br>(100 %)	22.61 ± 2.87	AN: 12.71 ± 1.68<br>Control:21.96 ± 2.81	N/A	Acylated ghrelin, des-acyl ghrelin, obestatin.	• Blood-plasma	ELISA.

Supplemental Table 2 (continues)

Author/Year	Country	Study Type	N [a] (% Female)	Age (Year) Mean ± SD or Range	BMI (kg/m^2) Mean ± SD or CI	Duration of Illness (Year)	Biomarker	Biomarker Source	Assay
Kaválková *et al.* 2012	Czech Republic	Longitudinal with intervention[†]	AN: 18 Control: 16 (100 %)	23.59 ± 1.33	AN: 15.58 ± 0.28 Control:21.8 ± 0.51		Albumin, cholesterol, free thyroxine, glucose, HDL, insulin, LDL, leptin, preadipocyte factor-1, total protein, thyroid stimulating hormone, triglycerides.	Blood-serum	ELISA, RIA, CLIA.
Amitani *et al.* 2013	Japan	Cross-sectional	AN: 9 Control: 9 (100 %)	20.17 ± 1.59	AN: 13.01 ± 0.23 Control:21.59 ± 0.43	1.71	Adiponectin, cholesterol fatty acids, glucose, HDL, insulin, LDL, triglycerides.	Blood-serum, Blood-plasma	Standard laboratory methods.
Giel *et al.* 2013	Germany	Cross-sectional	AN: 15 Control: 30 (100 %)	24.37 ± 3.62	AN: 15.4 ± 1.7 Control: 21.55 ± 1.65	N/A	Leptin.	Blood-serum	ELISA.
Nogueira *et al.* 2013	France	Longitudinal with intervention[†]	AN: 11 Control: 10 (100 %)	23.19 ± 1.41	AN: 13.1 ± 0.07 Control:22.3 ± 0.16	N/A	Adiponectin, alanine aminotransferase, aspartate aminotransferase, estradiol, free thyroxine, free triiodothyronine, GH, ghrelin, glucose, IGF-1, insulin, leptin, resistin, testosterone, thyroid stimulating hormone.	• Blood-serum	Automated analyzer, hexokinase oxidase method, CLIA, IRMA, ELISA.
Stengel *et al.* 2013	Germany	Cross-sectional	AN: 8 Control: 32 (60 %)	43.22 ± 9.55	AN: 12.6 ± 0.7 Control:43.63 ± 17.59	N/A	Albumin, cortisol, thyroid stimulating hormone, ghrelin, glucose, insulin, thyroid stimulating hormone, total protein.	• Blood-serum, blood-plasma	Standard laboratory methods, ELISA.
Terra *et al.* 2013	Spain	Cross-sectional	AN: 28 Control: 33 (100 %)	30.21 ± 2.93	AN: 16.8 ± 0.2 Control:21.8 ± 0.3	8.3	Cholesterol, c-peptide, glucose, HDL, insulin, LDL, triglycerides.	• Blood-plasma	Automated analyzer
Holsen *et al.* 2014	USA	Cross-sectional	AN: 13 Control: 12 (100 %)	21.90 ± 2.13	AN: 18.1 ± 0.8 Control:22.5 ± 1.4	4.9	Acylated ghrelin.	• Blood-plasma	RIA.

Supplemental Table 2 (continues)

Author/Year	Country	Study Type	N [a] (% Female)	Age (Year) Mean ± SD or Range	BMI (kg/m^2) Mean ± SD or CI	Duration of Illness (Year)	Biomarker	Biomarker Source	Assay
Barja-Fernández *et al.* 2015	Spain	Cross-sectional	AN: 30<br>Control: 119<br>(100 %)	36.10 ± 12.43	AN: 16.5 ±1.3<br>Control: 33.76 ± 11.62		Cholesterol, free thyroxine, free triiodithyronine, glucose, HDL, insulin, LDL, leptin, triglycerides, thyroid stimulating hormone.	Blood-plasma	IRMA, ELISA, CLIA.
Eddy *et al.* 2015	USA	Cross-sectional	AN: 75<br>Control: 22<br>(100 %)	26.03 ± 1.08	AN: 17.3 ± 0.1<br>Control:22.4 ± 0.4	6-10.2	brain-derived neurotrophic factor, leptin, peptide tyrosine tyrosine.	Blood-serum	RIA, ELISA.
Fazeli *et al.* 2015	USA	Cross-sectional	AN: 20<br>Control: 26<br>(100 %)	25.75 (SD: N/A)	AN: 17.8 (CI: 15.9,18.3)<br>Control:22.4 (CI: 21.4, 23.2)	10.3	Carboxy-terminal collagen crosslinks.	Blood-not specify	Fluoroimmunoassay
Fernandez-Aranda *et al.* 2015	Spain	Cross-sectional	AN: 64<br>Control: 175<br>(100 %)	28.87 ± 9.09	AN: 17.4 ± 1.4<br>Control: 28.88 ± 10.84	5.5	Cholecystokinin, ghrelin peptide tyrosine tyrosine.	Blood-serum	ELISA.
Galusca *et al.* 2015	France	Cross-sectional	AN: 23<br>Control: 36<br>(100 %)	22.78 ± 1.19	AN: 14.6 ± 0.5<br>Control: 18.12 ± 2.83	N/A	Cortisol, estradiol, free triiodothyronine, GH, IGF-1, leptin.	Blood-plasma	RIA, IRMA.
Schorr *et al.* 2015	USA	Cross-sectional	AN: 18<br>Control: 42<br>(100 %)	28.00 ± 7.00	AN: 18.2 ± 1.0<br>Control: 26.75 ± 5.50	N/A	Cortisol, estradiol.	• Blood-serum,<br>• Saliva	CLIA, EIA.
Urano *et al.* 2015	Japan	Cross-sectional	AN: 54<br>Control: 15<br>(100 %)	28.37 (SD: N/A)	AN: 14.9 (CI: 13.3,16.5)<br>Control: 20.1 (CI: 18.7, 21.4)	7.75-10.3	25-OH-vitamin D, adiponectin, albumin, bone alkaline phosphatase, calcium, estradiol, IGF-1, intact parathyroid hormone, leptin, osteocalcin, phosphorus.	• Blood-serum	BCP, RIA, CLIA, accucare calcium arsenazo III kit, Malachite green phosphate assay, latex particle-enhanced turbidimetric immunoassay.
Baskaran *et al.* 2016	USA	Cross-sectional	AN:13<br>Control: 24<br>(100 %)	27.80 ± 6.90	AN: 18.5 ± 0.9<br>Control: 26.65 ± 5.63		Cortisol, estradiol, ghrelin, IGF−1, insulin, peptide tyrosine tyrosine.	• Blood-serum,<br>• Urine	RIA, CLIA, EIA.

Supplemental Table 2 (continues)

Author/Year	Country	Study Type	N [a] (% Female)	Age (Year) Mean ± SD or Range	BMI (kg/m^2) Mean ± SD or CI	Duration of Illness (Year)	Biomarker	Biomarker Source	Assay
Germain *et al.* 2016	France	Cross-sectional	AN: 15<br>Control: 20<br>(100%)	21.66 ± 1.78	AN: 15.2 ± 0.92<br>Control: 18.65 ± 2.85	N/A	Testosterone.	Blood-plasma	RIA.
Gill *et al.* 2016	USA	Cross-sectional	AN:23<br>Control:18<br>(100 %)	26.25 ± 5.39	AN:16.8 ± 1.3<br>Control:22.1 ± 1.6	N/A	Estradiol.	Blood-serum	CLIA.
Baranowska-Bik *et al.* 2017	Poland	Cross-sectional	AN: 20<br>Control: 27<br>(100 %)	24.49 ± 6.97	AN: 15.62 ± 1.13<br>Control: 21.61 ± 2.08	N/A	Adiponectin, leptin resistin, soluble leptin receptor, visfatin.	Blood-plasma	RIA, ELISA.
Carlsson *et al.* 2017	Sweden	Cross-sectional	AN: 20<br>Control: 78<br>(100 %)	23.62 ± 3.38	AN: 16.6 ± 0.7<br>Control:24.9 ± 4.7	8	25-OH-vitamin D, Albumin, calcium, creatinine, leptin, parathyroid hormone.	Blood-serum, Blood-plasma	ELISA, CLIA, high-performance liquid chromatography, colorimetric method.
Cinkajzlova *et al.* 2017	Czech Republic	Longitudinal with intervention†	AN: 22<br>Control: 15<br>(100 %)	23.85 ± 1.33	AN: 15.4 ± 0.26<br>Control: 21.9 ± 0.54	9	Cholesterol, glucose, HDL, insulin, LDL, leptin, triglycerides.	Blood-not specify	RIA.
Estour *et al.* 2017	France	Longitudinal without intervention†	AN:40<br>Control:110<br>(100 %)	25.13 ± 6.37	AN: 16.0 ± 0.8<br>Control: 18.66 ± 2.76	N/A	ACTH, albumin, cortisol free thyroxine, free triiodothyronine, IGF-1, leptin, prolactin, thyroid stimulating hormone, testosterone.	• Blood-not specify	N/A
Matsumoto *et al.* 2017	Japan	Cross-sectional	AN: 19<br>Control: 22<br>(100 %)	23.29 ± 7.26	AN: 14.51 ± 1.81<br>Control: 20.83 ± 1.67	6.21	Brain-derived neurotrophic factor.	• Blood-serum	ELISA
Schorr *et al.* 2017	USA	Cross-sectional	AN: 16<br>Control: 43<br>(100 %)	28.27 ± 7.78	AN: 18.3 ± 0.9<br>Control: 26.66 ± 6.42	N/A	25-OH-vitamin D, IGF-1, leptin, oxytocin, parathyroid hormone.	• Blood-serum	ELISA, RIA, CLIA, high-performance liquid chromatography.
Burdo *et al.* 2020	Canada	Cross-sectional	AN: 53<br>Control: 36<br>(100 %)	25.37 ± 7.3	AN: 15.2 ± 1.49<br>Control: 23.31 ± 3.0	8.5	Vitamin B12.	• Blood-not specify	ELISA
Vaz-Leal *et al.* 2020	Span	Longitudinal with intervention†	AN: 24<br>Control: 58<br>(100 %)	21.53 ± 3.49	AN: 16.8 ± 1.0<br>Control: 21.7 ± 1.3	N/A	Lymphocyte	• Blood-not specify	Fluoroimmunoassay

Note. † Only baseline data were used for analysis; [a] number of participants in AN include AN restrictive and binge/purging type; SD = standard deviation; N/A = not available; ACTH = Adrenocorticotropic hormone; CRP = c-reactive protein; GH = growth hormone; HDL = high-density lipoprotein cholesterol; LDL = low-density lipoprotein) cholesterol; IGF−1 = insulin-like growth factor 1; IGFBP-1 = insulin-like growth factor binding protein-1; IGFBP-2 = Insulin-like growth factors binding protein-2; IGFBP−3 = insulin-like growth factor binding protein-3; CI= 95% confidence interval; BCP = Bromocresol purple method; CLIA = chemiluminiscence immunoassay; EIA = enzyme immunoassay; ELISA = enzyme-linked immunosorbent assay; IRMA = immunoradiometric assay; RIA = radioimmunoassay.

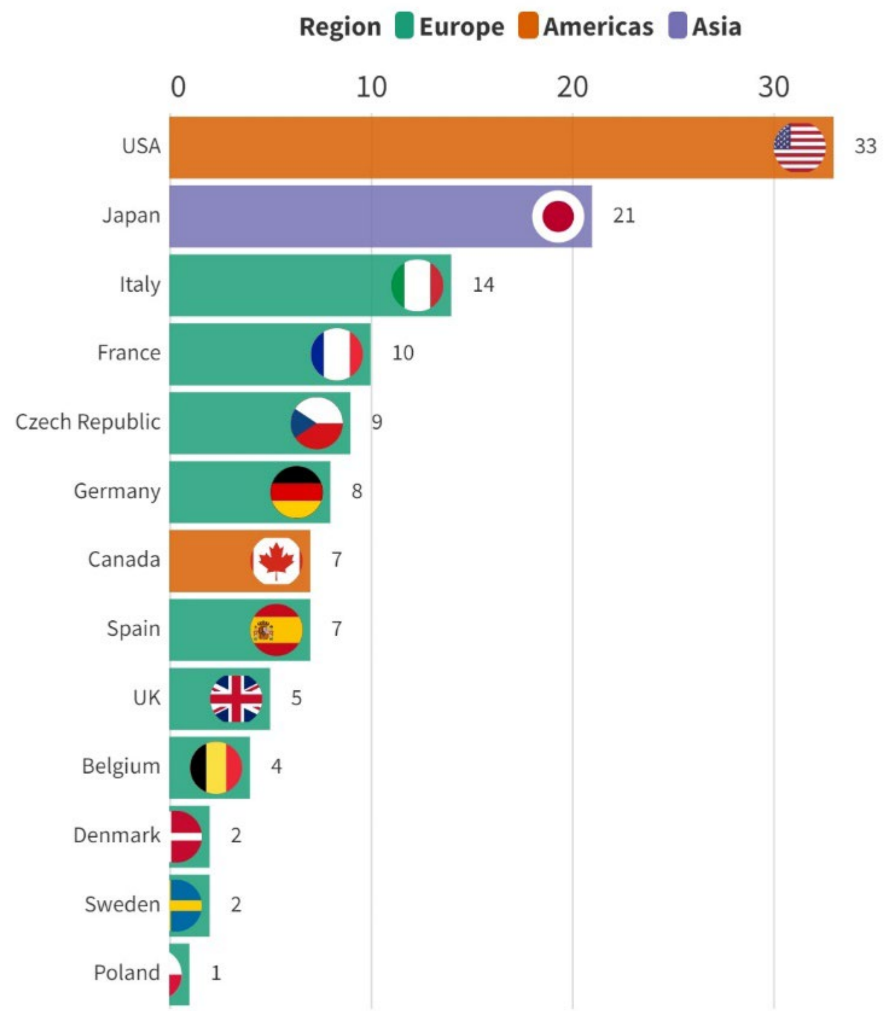

Supplemental Figure 1. List of countries of included studies (n =123).

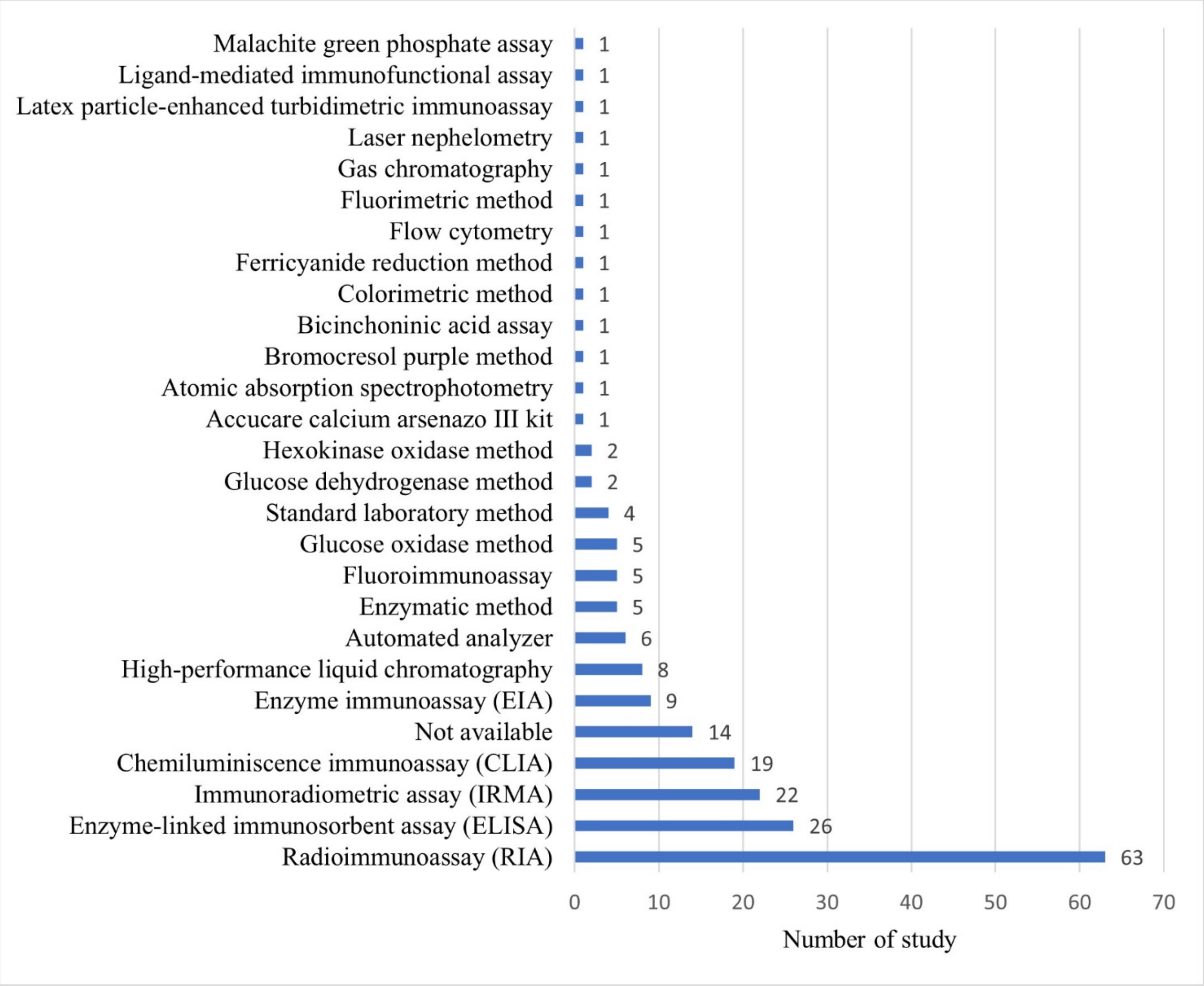

Supplemental Figure 2. List of assay methods of included studies (n = 123). Forty-seven studies utilized more than one type of assay.

Supplemental Table 3. Quality assessment of the included studies (n = 123)

Author	Year	Was a random or pseudo random sample used?	Was the inclusion criteria clearly defined?	Were confounding factors identified and control strategies stated?	Were outcomes assessed using objective criteria	Was there sufficient description of the groups?	Was follow up carried out over a sufficient time period?	Were the outcomes of people who withdrew described and included in analysis?	Were outcomes measured in a reliable way?	Was appropriate statistical analysis used?	Quality Score ("Yes"+1, "No" 0, "Unclear" -1, "non-applicable" 0)
Ainley	1991	No	Yes	No	Yes	Yes	Na	Na	Yes	Yes	5
Amitani	2013	No	Yes	No	Yes	Yes	Na	Na	Yes	Yes	5
Baranowska-Bik	2017	No	Yes	No	Yes	Yes	Yes	Unclear	Yes	Yes	5
Barja-Fernández	2015	No	Yes	Yes	Yes	Yes	Na	Na	Yes	Yes	6
Bartak	2004	No	Yes	No	Yes	Yes	Na	Na	Yes	Yes	5
Baskaran	2016	No	Yes	No	Yes	Yes	Yes	Na	Yes	Yes	6
Bossu	2007	No	Yes	No	Yes	Yes	Na	Na	Yes	Yes	5
Brick	2010	No	Yes	No	Yes	Yes	Na	Na	Yes	Yes	5
Broglio	2004	No	Yes	No	Yes	Yes	Yes	Na	Yes	Yes	6
Carlsson	2017	No	Yes	No	Yes	Yes	Na	Na	Yes	Yes	5
Cassanueva	1997	No	Yes	No	Yes	Yes	Na	Na	Yes	Yes	5
Case	1997	No	Yes	No	Yes	Yes	Na	Na	Yes	Yes	5
Cason	1986	No	Yes	No	Yes	Yes	Na	Na	Yes	Yes	5
Casper	1988	No	Yes	Yes	Yes	Yes	Yes	Unclear	Yes	Yes	6
Castillo	1985	No	Yes	No	Yes	Yes	Yes	Na	Yes	Yes	6
Cinkajzlova	2017	No	Yes	No	Yes	Yes	Yes	Na	Yes	Yes	6
Connan	2007	No	Yes	Yes	Yes	Yes	Yes	No	Yes	Yes	7
Corcos	2001	No	Yes	No	Yes	Yes	Na	Na	Yes	Yes	5
Counts	1992	No	Yes	No	Yes	Yes	Yes	Unclear	Yes	Yes	5
Dempsey	1984	No	Yes	No	Yes	Yes	Na	Na	Yes	Yes	5
Djurovic	2004	No	Yes	No	Yes	Yes	Yes	Na	Yes	Yes	6
Dominguez	2007	No	Yes	No	Yes	Yes	Yes	Na	Yes	Yes	6
Dostalova	2007	No	Yes	No	Yes	Yes	Yes	Na	Yes	Yes	6
Dostalova	2008	No	Yes	No	Yes	Yes	Na	Na	Yes	Yes	5
Dostalova	2009	No	Yes	No	Yes	Yes	Na	Na	Yes	Yes	5

Supplemental Table 3 (continues)

Author	Year	Was a random or pseudo random sample used?	Was the inclusion criteria clearly defined?	Were confounding factors identified and control strategies stated?	Were outcomes assessed using objective criteria	Was there sufficient description of the groups?	Was follow up carried out over a sufficient time period?	Were the outcomes of people who withdrew described and included in analysis?	Were outcomes measured in a reliable way?	Was appropriate statistical analysis used?	Quality Score (“Yes”+1, “No” 0, “Unclear” -1, “non-applicable” 0)
Duclos	1999	No	Yes	No	Yes	Yes	Yes	Na	Yes	Yes	6
Eddy	2015	No	Yes	Yes	Yes	Yes	Na	Na	Yes	Yes	6
Estour	2017	No	Yes	No	Yes	Yes	Na	Na	Yes	Yes	5
Fazeli	2010	No	Yes	No	Yes	Yes	Na	Na	Yes	Yes	5
Fazeli	2015	No	Yes	Yes	Yes	Yes	Na	Na	Yes	Yes	6
Fernandez-Aranda	2015	No	Yes	Yes	Yes	Yes	Na	Na	Yes	Yes	6
Ferrari	1990	No	Yes	No	Yes	Yes	Yes	Na	Yes	Yes	6
Ferron	1997	No	Yes	No	Yes	Yes	Na	Na	Yes	Yes	5
Flierl	2011	No	Yes	No	Yes	Yes	Yes	Unclear	Yes	Yes	5
Foppiani	1996	No	Yes	No	Yes	Yes	Yes	Na	Yes	Yes	6
Frey	2003	No	Yes	No	Yes	Yes	Na	Na	Yes	Yes	5
Fukuda	1999	No	Yes	No	Yes	Yes	Na	Na	Yes	Yes	5
Galusca	2015	No	Yes	No	Yes	Yes	Na	Na	Yes	Yes	5
Geracioti	1992	No	Yes	No	Yes	Yes	Yes	Na	Yes	Yes	6
Germain	2007	No	Yes	No	Yes	Yes	Yes	Na	Yes	Yes	6
Germain	2016	No	Yes	No	Yes	Yes	Na	Na	Yes	Yes	5
Germain	2009	No	Yes	No	Yes	Yes	Yes	Na	Yes	Yes	6
Gianotto	2000	No	Yes	No	Yes	Yes	Yes	Na	Yes	Yes	6
Giel	2013	No	Yes	No	Yes	Yes	Yes	Na	Yes	Yes	6
Gill	2016	No	Yes	Yes	Yes	Yes	Na	Na	Yes	Yes	6
Grindpoon	1996	No	Yes	No	Yes	Yes	Na	Na	Yes	Yes	5
Gwirtsman	1989	No	Yes	No	Yes	Yes	Yes	Na	Yes	Unclear	4
Hadigan	1995	No	Yes	No	Yes	Yes	Na	Na	Yes	Yes	5
Haluzi´k	1999	No	Yes	No	Yes	Yes	Na	Na	Yes	Yes	5
Haluzikova	2009	No	Yes	No	Yes	Yes	Na	Na	Yes	Yes	5

Supplemental Table 3 (continues)

Author	Year	Was a random or pseudo random sample used?	Was the inclusion criteria clearly defined?	Were confounding factors identified and control strategies stated?	Were outcomes assessed using objective criteria	Was there sufficient description of the groups?	Was follow up carried out over a sufficient time period?	Were the outcomes of people who withdrew described and included in analysis?	Were outcomes measured in a reliable way?	Was appropriate statistical analysis used?	Quality Score ("Yes"+1, "No" 0, "Unclear" -1, "non-applicable" 0)
Harada	2008	No	Yes	No	Yes	Yes	Yes	Na	Yes	Yes	6
Hellzén	2003	No	Yes	Yes	Yes	Yes	Na	Na	Yes	Yes	6
Hildebrandt	2000	No	Yes	No	Yes	Yes	Na	Na	Yes	Yes	5
Holsen	2014	No	Yes	Yes	Yes	Yes	Na	Na	Yes	Yes	6
Innis	2009	No	Yes	No	Yes	Yes	Na	Na	Yes	Yes	5
Jacoangeli	2002	No	Yes	No	Yes	Yes	Na	Na	Yes	Unclear	3
Johnston	1984	No	Yes	No	Yes	Yes	Yes	Na	Yes	Yes	6
Kavalkova	2012	No	Yes	No	Yes	Yes	Yes	Na	Yes	Yes	6
Kaye	1990	No	Yes	No	Yes	Yes	Yes	Unclear	Yes	Yes	5
Kennedy	1993	No	Yes	No	Yes	Yes	Yes	Na	Yes	Yes	6
Kinzig	2007	No	Yes	No	Yes	Yes	Yes	Na	Yes	Yes	6
Krsek	2003	No	Yes	No	Yes	Yes	Na	Na	Yes	Yes	5
Lawson	2009	No	Yes	No	Yes	Yes	Na	Na	Yes	Yes	5
Lawson	2007	No	Yes	No	Yes	Yes	Na	Na	Yes	Yes	5
Lawson	2011	No	Yes	Yes	Yes	Yes	Na	Na	Yes	Yes	6
Lear	1999	No	Yes	No	Yes	Yes	Na	Na	Yes	Yes	5
Lesem	1989	No	Yes	No	Yes	Yes	Yes	Na	Yes	Yes	6
Matsumoto	2017	No	Yes	No	Yes	Yes	Na	Na	Yes	Yes	5
Mayer	2005	No	Yes	No	Yes	Yes	Yes	Na	Yes	Yes	6
Miljic	2006	No	Yes	No	Yes	Yes	Yes	Na	Yes	Yes	6
Miller	2007	No	Yes	No	Yes	Yes	Na	Na	Yes	Yes	5
Mondelli	2006	No	Yes	No	Yes	Yes	Yes	Na	Yes	Yes	6
Monteleone	2008	No	Yes	No	Yes	Yes	Na	Na	Yes	Yes	5
Monteleone	2000a	No	Yes	No	Yes	Yes	Yes	Na	Yes	Yes	6
Monteleone	2000b	No	Yes	No	Yes	Yes	Na	Na	Yes	Yes	5
Monteleone	2005	No	Yes	No	Yes	Yes	Na	Na	Yes	Yes	5

Supplemental Table 3 (continues)

Author	Year	Was a random or pseudo random sample used?	Was the inclusion criteria clearly defined?	Were confounding factors identified and control strategies stated?	Were outcomes assessed using objective criteria	Was there sufficient description of the groups?	Was follow up carried out over a sufficient time period?	Were the outcomes of people who withdrew described and included in analysis?	Were outcomes measured in a reliable way?	Was appropriate statistical analysis used?	Quality Score ("Yes"+1, "No" 0, "Unclear" -1, "non-applicable" 0)
Monteleone	2002	No	Yes	No	Yes	Yes	Na	Na	Yes	Yes	5
Mori	1990	No	Yes	No	Yes	Yes	Yes	Na	Yes	No	5
Mortola	1993	No	Yes	No	Yes	Yes	Yes	Na	Yes	Yes	6
Nakahara	2007	No	Yes	Yes	Yes	Yes	Yes	Na	Yes	Yes	7
Nakahara	2008	No	Yes	No	Yes	Yes	Na	Na	Yes	Yes	5
Nakai	1999	No	Yes	No	Yes	Yes	Na	Na	Yes	Yes	5
Nakai	2001	No	Yes	No	Yes	Yes	Yes	Na	Yes	Yes	6
Nakazato	2009	No	Yes	No	Yes	Yes	Na	Na	Yes	Yes	5
Nedvidkova	2004	No	Yes	No	Yes	Yes	Yes	Na	Yes	Yes	6
Nishita	1989	No	Yes	No	Yes	Yes	Yes	Na	Yes	Yes	6
Nogueira	2013	No	Yes	Yes	Yes	Yes	Yes	Na	Yes	Yes	7
Onur	2005	No	Yes	No	Yes	Yes	Yes	No	Yes	Yes	6
Popovic	2004	No	Yes	No	Yes	Yes	Na	Na	Yes	Yes	5
Rigaud	2000	No	Yes	No	Yes	Yes	Yes	Na	Yes	Yes	6
Rigaud	2007	No	Yes	No	Yes	Yes	Yes	Na	Yes	Yes	6
Saito	2007	No	Yes	No	Yes	Yes	Na	Na	Yes	Unclear	3
Schorr	2015	No	Yes	Yes	Yes	Yes	Na	Na	Yes	Yes	6
Schorr	2017	No	Yes	Yes	Yes	Yes	Na	Na	Yes	Yes	6
Stengel	2013	No	Yes	No	Yes	Yes	Na	Na	Yes	Yes	5
Støving	2002	No	Yes	No	Yes	Yes	Yes	Na	Yes	Yes	6
Støving	2007	No	Yes	No	Yes	Yes	Na	Na	Yes	Yes	5
Tagami	2004	No	Yes	No	Yes	Yes	Yes	Na	Yes	Yes	6
Tamia	1991	No	Yes	No	Yes	Yes	Yes	Na	Yes	Yes	6
Tanaka	2004	No	Yes	No	Yes	Yes	Yes	Na	Yes	Yes	6
Terra	2013	No	Yes	No	Yes	Yes	Yes	Na	Yes	Yes	6
Uehara	2011	No	Yes	No	Yes	Yes	Yes	Na	Yes	Yes	6

Supplemental Table 3 (continues)

Author	Year	Was a random or pseudo random sample used?	Was the inclusion criteria clearly defined?	Were confounding factors identified and control strategies stated?	Were outcomes assessed using objective criteria	Was there sufficient description of the groups?	Was follow up carried out over a sufficient time period?	Were the outcomes of people who withdrew described and included in analysis?	Were outcomes measured in a reliable way?	Was appropriate statistical analysis used?	Quality Score ("Yes"+1, "No" 0, "Unclear" -1, "non-applicable" 0)
Urano	2015	No	Yes	No	Yes	Yes	Na	Na	Yes	Yes	5
Viapiana	2007	No	Yes	No	Yes	Yes	Yes	Na	Yes	Yes	6
Wilhelm	2010	No	Yes	No	Yes	Yes	Yes	Yes	Yes	Yes	7
Zumoff	1983	No	No	No	Yes	Yes	Yes	Na	Yes	Yes	5
Zuniga-Guajardo	1986	No	Yes	No	Yes	Yes	Yes	Na	Yes	Yes	6
Arimura	2010	No	Yes	No	Yes	Yes	Yes	Na	Yes	Yes	6
Brambilla	2003	No	Yes	No	Yes	Yes	Na	Na	Yes	Yes	5
Delporte	2003	No	Yes	No	Yes	Yes	Na	Na	Yes	Yes	5
Fujimoto	1997	No	Yes	No	Yes	Yes	Yes	Na	Yes	Yes	6
Fukushima	1993	No	Yes	No	Yes	Yes	Yes	Na	Yes	Yes	6
Grinspoon	2004	No	Yes	No	Yes	Yes	Yes	Na	Yes	Yes	6
Laanfranco	2004	No	Yes	No	Yes	Yes	Yes	Na	Yes	Yes	6
Monteleone	1999	No	Yes	No	Yes	Yes	Na	Na	Yes	Yes	5
Murialdo	2007	No	Yes	No	Yes	Yes	Na	Na	Yes	Yes	5
Ohwada	2007	No	Yes	No	Yes	Yes	Na	Na	Yes	Yes	5
Otto	2007	No	Yes	No	Yes	Yes	Yes	Na	Yes	Yes	6
Pirke	1992	No	Yes	No	Yes	Yes	Na	Na	Yes	Unclear	3
Takeno	1990	No	Yes	No	Yes	Yes	Yes	Na	Yes	Yes	6
Terashi	2011	No	Yes	No	Yes	Yes	Yes	Unclear	Yes	Yes	5
Burdo	2020	No	Yes	No	Yes	Yes	Na	Na	Yes	Yes	5
Vaz-Leal	2020	No	Yes	No	Yes	Yes	Yes	Na	Yes	Yes	6

Supplemental Document 3. Individual Forest Plots for all 52 Peripheral Biomarkers.

N = number of participants; M = mean value of the biomarker; SD = standard deviation; SMD = standardized mean difference; RE model = random-effects meta-analysis model; 95% CI = 95% confidence intervals.

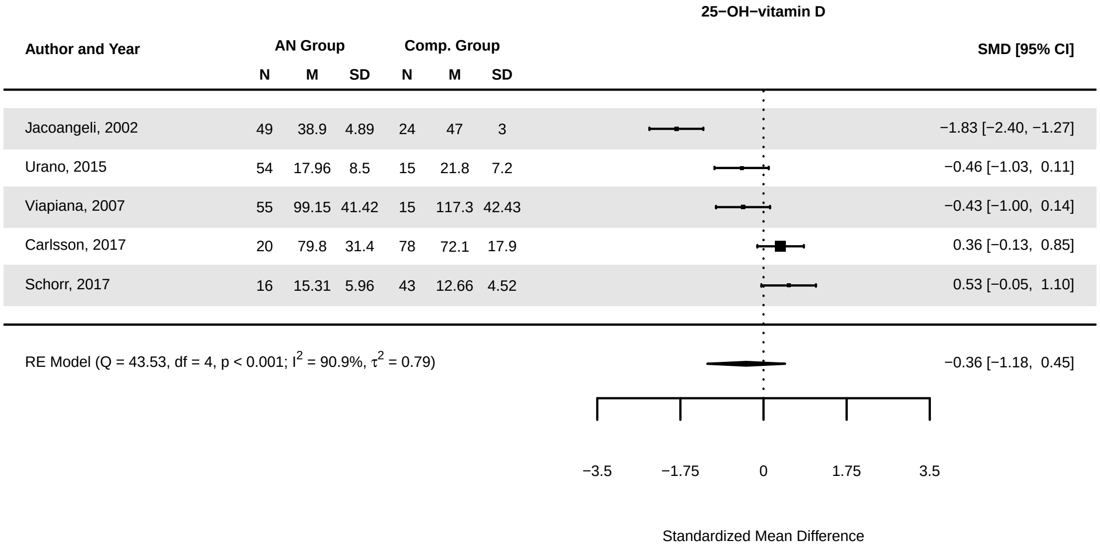

Acylated ghrelin

Author and Year	AN Group N	AN Group M	AN Group SD	Comp. Group N	Comp. Group M	Comp. Group SD	SMD [95% CI]
Uehara, 2011	9	120.2	95.54	9	77.88	74.04	0.47 [−0.42, 1.36]
Germain, 2009	15	1123	782	19	811	364.04	0.52 [−0.15, 1.19]
Holsen, 2014	13	59.5	75.6	12	14.4	12.1	0.79 [−0.00, 1.58]
Terashi, 2011	10	28.6	6.48	10	22.3	6.48	0.93 [0.04, 1.82]
Harada, 2008	10	28.6	6.3	10	22.3	6.48	0.94 [0.06, 1.83]
Nakahara, 2008	11	33.7	8.53	21	20.33	10.99	1.27 [0.50, 2.05]
RE Model (Q = 2.79, df = 5, p = 0.732; I^2 = 0.0%, τ^2 = 0.00)							0.81 [0.48, 1.14]

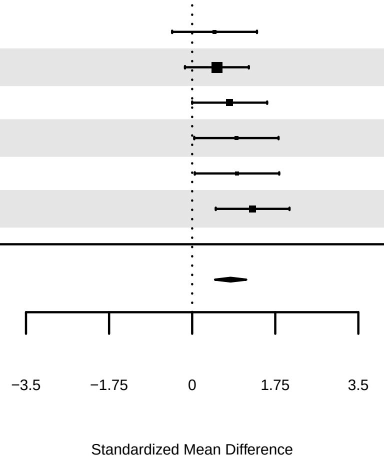

Adiponectin

Author and Year	AN Group N	AN Group M	AN Group SD	Comp. Group N	Comp. Group M	Comp. Group SD	SMD [95% CI]
Nogueira, 2013	11	6.7	1.17	10	10.9	1.56	−2.95 [−4.16, −1.73]
Amitani, 2013	9	6.16	3.7	9	7.59	1.72	−0.47 [−1.37, 0.42]
Tagami, 2004	31	11	7.8	31	14.99	9.78	−0.45 [−0.94, 0.05]
Miljic, 2006	9	33.5	24.04	10	37.2	12.6	−0.19 [−1.05, 0.67]
Baranowska−Bik, 2017	20	6.89	2.62	27	5.39	1.91	0.66 [0.08, 1.24]
Urano, 2015	54	19.07	9.25	15	13.1	5.84	0.68 [0.11, 1.26]
Støving, 2007	35	13.12	4.84	24	9.7	3.9	0.75 [0.22, 1.28]
Delporte, 2003	26	16.1	4.5	24	11.8	4.31	0.96 [0.38, 1.54]
Haluzikova, 2009	19	36.5	18.37	16	19.9	8.86	1.09 [0.40, 1.79]
Dostalova, 2008	17	36.5	17.32	17	19.9	9.16	1.17 [0.46, 1.88]
Nakahara, 2008	11	13.7	6	21	8.61	2.56	1.23 [0.45, 2.00]
RE Model (Q = 65.19, df = 10, p < 0.001; I^2 = 90.2%, τ^2 = 1.05)							0.29 [−0.36, 0.93]

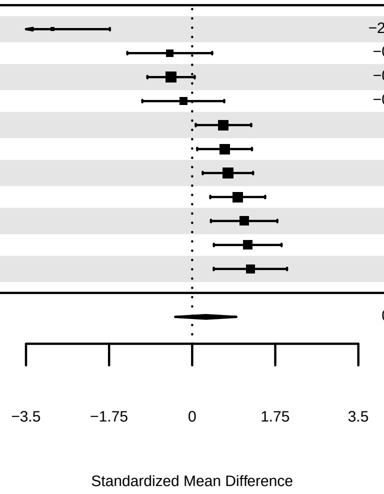

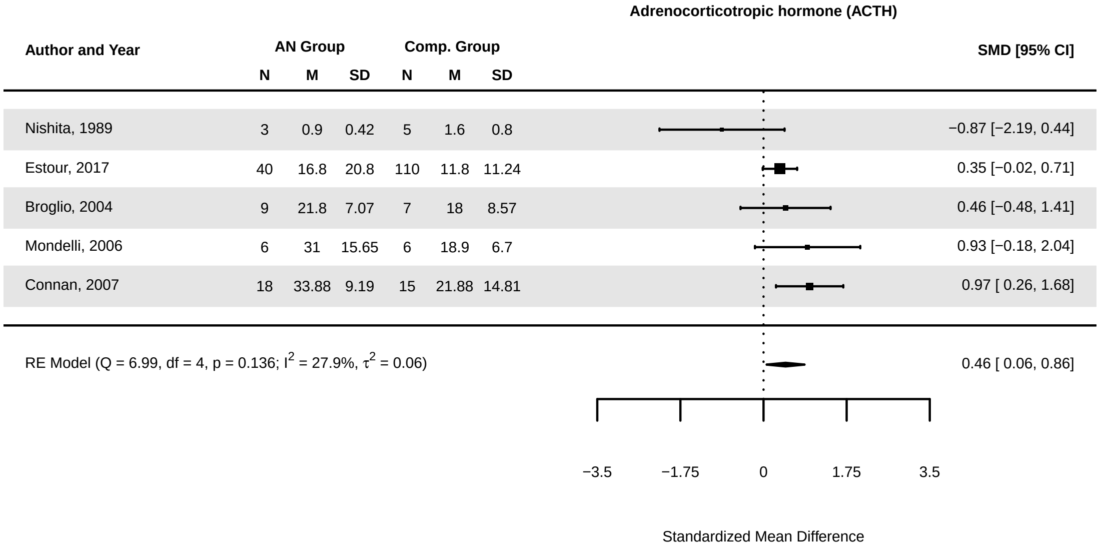
Adrenocorticotropic hormone (ACTH)
Author and Year
AN Group
Comp. Group
N M SD N M SD
SMD [95% CI]
Nishita, 1989 3 0.9 0.42 5 1.6 0.8 −0.87 [−2.19, 0.44]
Estour, 2017 40 16.8 20.8 110 11.8 11.24 0.35 [−0.02, 0.71]
Broglio, 2004 9 21.8 7.07 7 18 8.57 0.46 [−0.48, 1.41]
Mondelli, 2006 6 31 15.65 6 18.9 6.7 0.93 [−0.18, 2.04]
Connan, 2007 18 33.88 9.19 15 21.88 14.81 0.97 [0.26, 1.68]
RE Model (Q = 6.99, df = 4, p = 0.136; I^2 = 27.9%, τ^2 = 0.06) 0.46 [0.06, 0.86]
−3.5 −1.75 0 1.75 3.5
Standardized Mean Difference

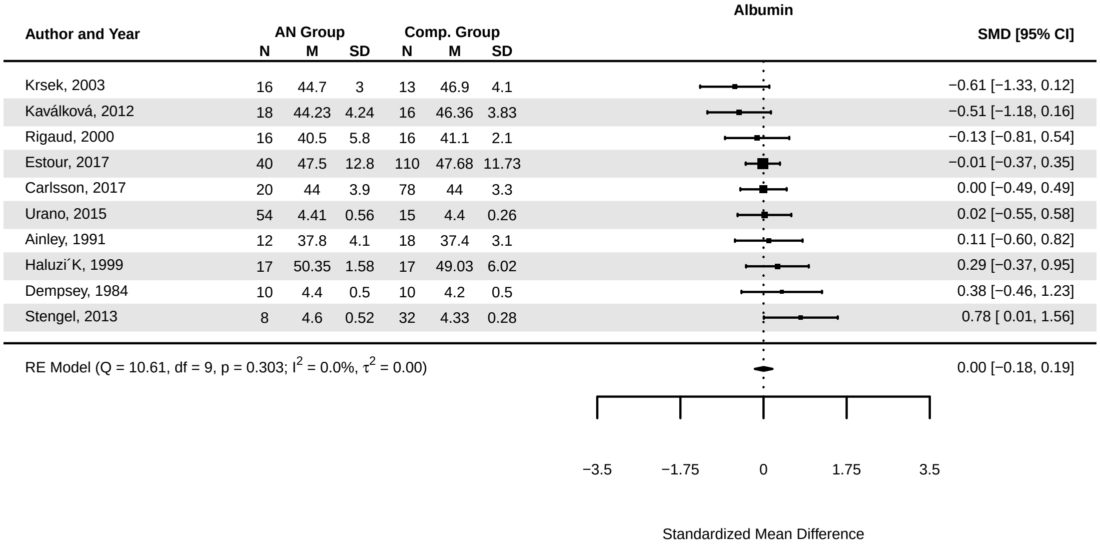
Albumin
Author and Year
AN Group
Comp. Group
N M SD N M SD
SMD [95% CI]
Krsek, 2003 16 44.7 3 13 46.9 4.1 −0.61 [−1.33, 0.12]
Kaválková, 2012 18 44.23 4.24 16 46.36 3.83 −0.51 [−1.18, 0.16]
Rigaud, 2000 16 40.5 5.8 16 41.1 2.1 −0.13 [−0.81, 0.54]
Estour, 2017 40 47.5 12.8 110 47.68 11.73 −0.01 [−0.37, 0.35]
Carlsson, 2017 20 44 3.9 78 44 3.3 0.00 [−0.49, 0.49]
Urano, 2015 54 4.41 0.56 15 4.4 0.26 0.02 [−0.55, 0.58]
Ainley, 1991 12 37.8 4.1 18 37.4 3.1 0.11 [−0.60, 0.82]
Haluzi´K, 1999 17 50.35 1.58 17 49.03 6.02 0.29 [−0.37, 0.95]
Dempsey, 1984 10 4.4 0.5 10 4.2 0.5 0.38 [−0.46, 1.23]
Stengel, 2013 8 4.6 0.52 32 4.33 0.28 0.78 [0.01, 1.56]
RE Model (Q = 10.61, df = 9, p = 0.303; I^2 = 0.0%, τ^2 = 0.00) 0.00 [−0.18, 0.19]
−3.5 −1.75 0 1.75 3.5
Standardized Mean Difference

Bone alkaline phosphatase

Author and Year	AN Group N	AN Group M	AN Group SD	Comp. Group N	Comp. Group M	Comp. Group SD	SMD [95% CI]
Viapiana, 2007	55	37.47	12.93	15	46.67	16.45	−0.66 [−1.24, −0.09]
Urano, 2015	54	13.61	5.9	15	8.9	1.83	0.88 [0.29, 1.46]
Ohwada, 2007	26	22.97	3.76	7	16.9	2.2	1.69 [0.78, 2.60]
RE Model (Q = 23.30, df = 2, p < 0.001; I^2 = 91.7%, τ^2 = 1.28)							0.60 [−0.74, 1.95]

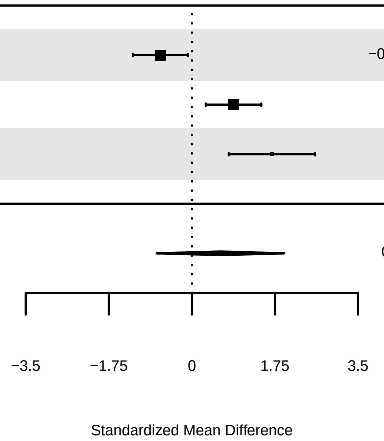

Brain−derived neurotrophic factor (BDNF)

Author and Year	AN Group N	AN Group M	AN Group SD	Comp. Group N	Comp. Group M	Comp. Group SD	SMD [95% CI]
Nakazato, 2009	29	11.7	4.9	28	15.1	5.5	−0.64 [−1.17, −0.12]
Matsumoto, 2017	19	13.96	6.02	22	16.58	3.5	−0.53 [−1.14, 0.08]
Eddy, 2015	75	14.8	2.05	22	14.6	1.4	0.10 [−0.37, 0.57]
Eddy, 2015	75	14.8	2.05	22	14.6	1.4	0.10 [−0.37, 0.57]
RE Model (Q = 7.03, df = 3, p = 0.071; I^2 = 57.5%, τ^2 = 0.09)							−0.22 [−0.61, 0.18]

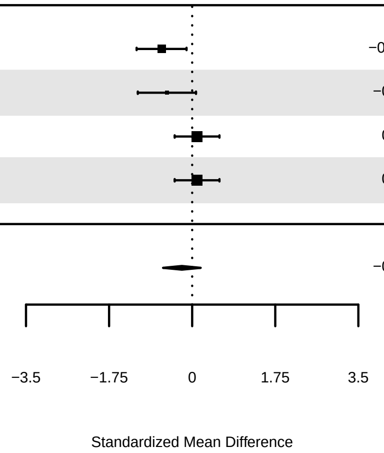

C−peptide

Author and Year	AN Group N	AN Group M	AN Group SD	Comp. Group N	Comp. Group M	Comp. Group SD	SMD [95% CI]
Zuniga−Guajardo, 1986	9	0.26	0.08	7	0.41	0.04	−2.15 [−3.35, −0.96]
Miljic, 2006	9	0.33	0.22	10	0.36	0.21	−0.13 [−0.99, 0.73]
Delporte, 2003	26	0.68	0.45	24	0.52	0.09	0.48 [−0.08, 1.03]
Terra, 2013	28	1.4	0.22	33	1.3	0.09	0.61 [0.10, 1.11]
RE Model (Q = 18.80, df = 3, p < 0.001; I^2 = 90.6%, τ^2 = 1.23)							−0.21 [−1.37, 0.95]

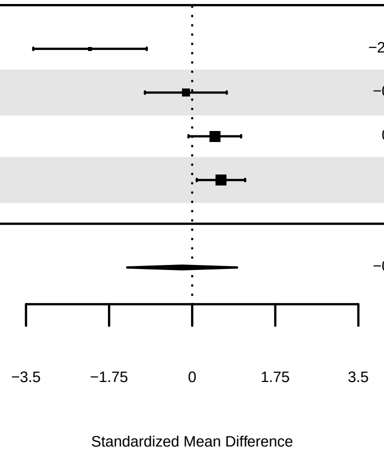

C−reactive protein

Author and Year	AN Group N	AN Group M	AN Group SD	Comp. Group N	Comp. Group M	Comp. Group SD	SMD [95% CI]
Haluzikova, 2009	19	0.9	2.75	16	3.2	4.45	−0.62 [−1.29, 0.05]
Dostalova, 2008	17	0.9	2.6	17	3.2	4.6	−0.60 [−1.27, 0.07]
Stengel, 2013	8	0.2	0.26	32	1.13	1.92	−0.52 [−1.29, 0.24]
RE Model (Q = 0.04, df = 2, p = 0.982; I^2 = 0.0%, τ^2 = 0.00)							−0.59 [−0.99, −0.18]

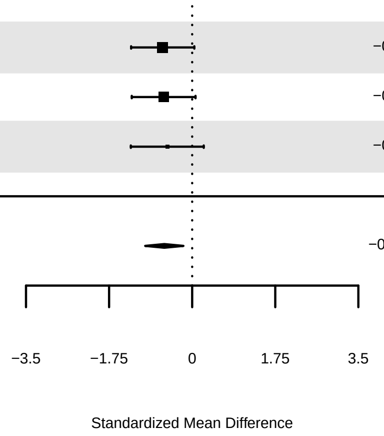

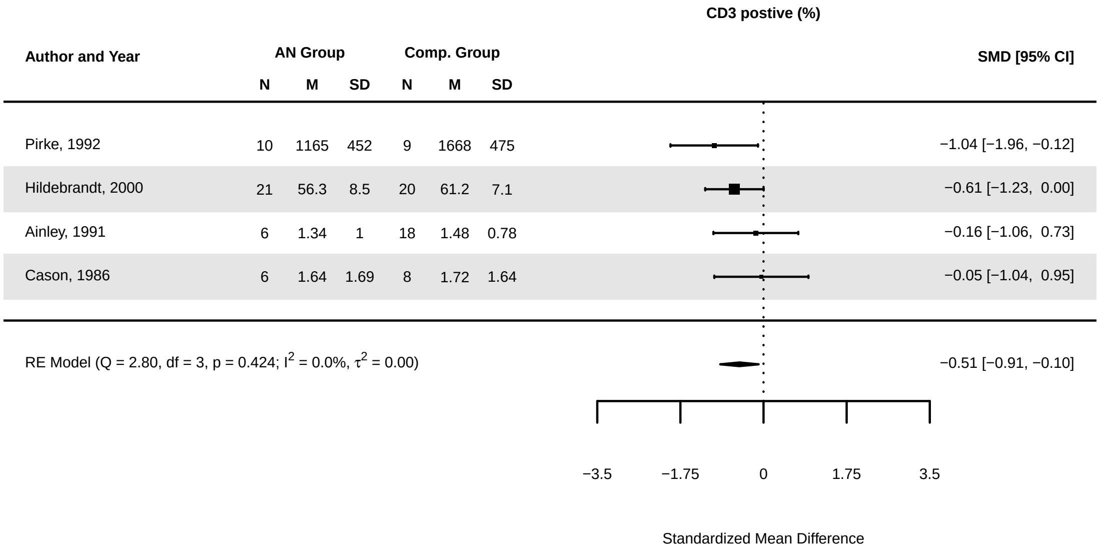
CD3 postive (%)
Author and Year
AN Group
Comp. Group
N M SD N M SD
SMD [95% CI]
Pirke, 1992 10 1165 452 9 1668 475 −1.04 [−1.96, −0.12]
Hildebrandt, 2000 21 56.3 8.5 20 61.2 7.1 −0.61 [−1.23, 0.00]
Ainley, 1991 6 1.34 1 18 1.48 0.78 −0.16 [−1.06, 0.73]
Cason, 1986 6 1.64 1.69 8 1.72 1.64 −0.05 [−1.04, 0.95]
RE Model (Q = 2.80, df = 3, p = 0.424; I2 = 0.0%, τ2 = 0.00) −0.51 [−0.91, −0.10]
−3.5 −1.75 0 1.75 3.5
Standardized Mean Difference

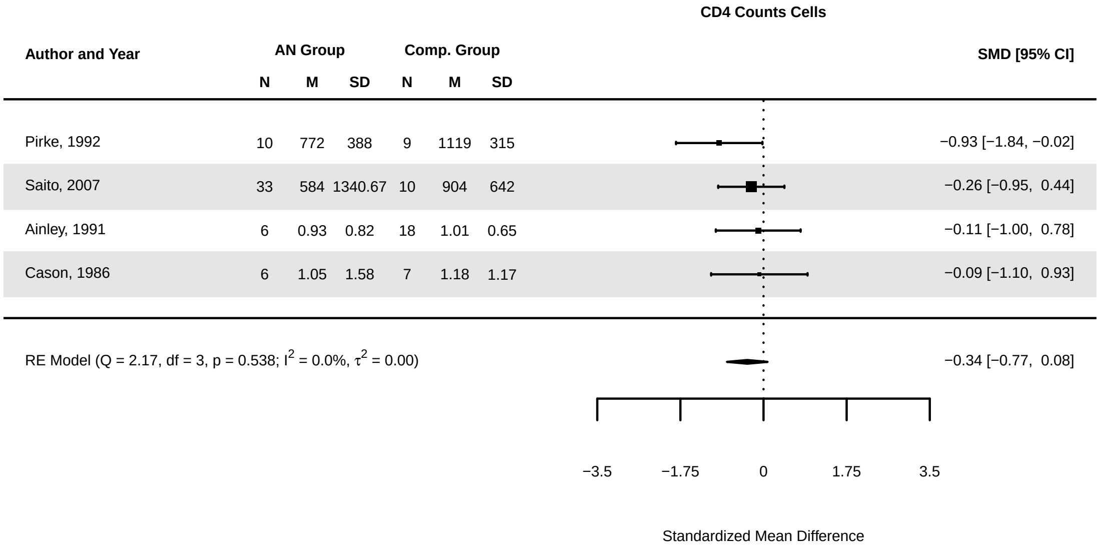
CD4 Counts Cells
Author and Year
AN Group
Comp. Group
N M SD N M SD
SMD [95% CI]
Pirke, 1992 10 772 388 9 1119 315 −0.93 [−1.84, −0.02]
Saito, 2007 33 584 1340.67 10 904 642 −0.26 [−0.95, 0.44]
Ainley, 1991 6 0.93 0.82 18 1.01 0.65 −0.11 [−1.00, 0.78]
Cason, 1986 6 1.05 1.58 7 1.18 1.17 −0.09 [−1.10, 0.93]
RE Model (Q = 2.17, df = 3, p = 0.538; I2 = 0.0%, τ2 = 0.00) −0.34 [−0.77, 0.08]
−3.5 −1.75 0 1.75 3.5
Standardized Mean Difference

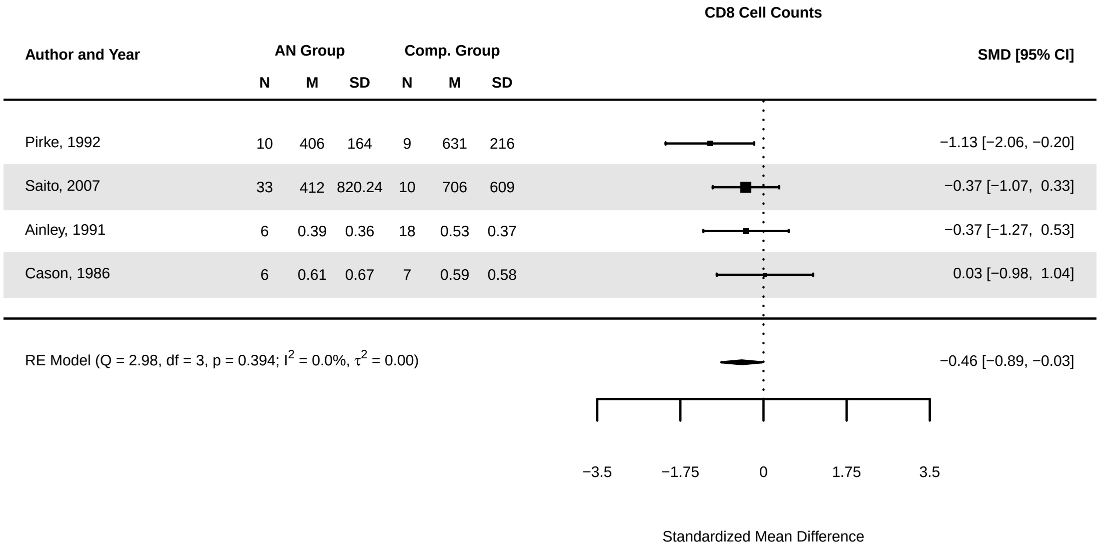
CD8 Cell Counts
Author and Year
AN Group
Comp. Group
SMD [95% CI]
N M SD N M SD
Pirke, 1992 10 406 164 9 631 216 −1.13 [−2.06, −0.20]
Saito, 2007 33 412 820.24 10 706 609 −0.37 [−1.07, 0.33]
Ainley, 1991 6 0.39 0.36 18 0.53 0.37 −0.37 [−1.27, 0.53]
Cason, 1986 6 0.61 0.67 7 0.59 0.58 0.03 [−0.98, 1.04]
RE Model (Q = 2.98, df = 3, p = 0.394; I2 = 0.0%, τ2 = 0.00) −0.46 [−0.89, −0.03]
−3.5
−1.75
0
1.75
3.5
Standardized Mean Difference

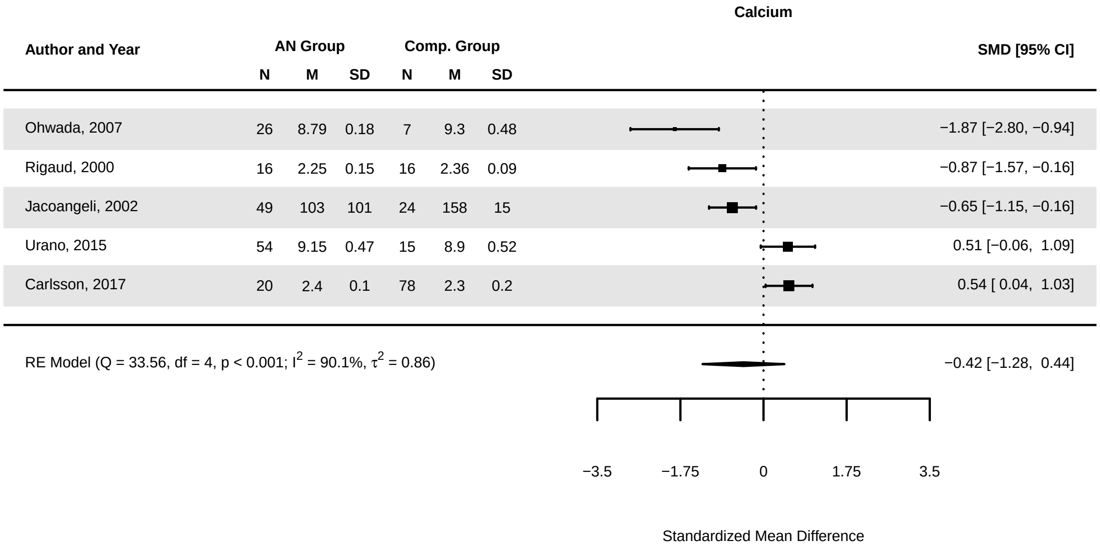
Calcium
Author and Year
AN Group
Comp. Group
SMD [95% CI]
N M SD N M SD
Ohwada, 2007 26 8.79 0.18 7 9.3 0.48 −1.87 [−2.80, −0.94]
Rigaud, 2000 16 2.25 0.15 16 2.36 0.09 −0.87 [−1.57, −0.16]
Jacoangeli, 2002 49 103 101 24 158 15 −0.65 [−1.15, −0.16]
Urano, 2015 54 9.15 0.47 15 8.9 0.52 0.51 [−0.06, 1.09]
Carlsson, 2017 20 2.4 0.1 78 2.3 0.2 0.54 [0.04, 1.03]
RE Model (Q = 33.56, df = 4, p < 0.001; I2 = 90.1%, τ2 = 0.86) −0.42 [−1.28, 0.44]
−3.5
−1.75
0
1.75
3.5
Standardized Mean Difference

Carboxy−terminal collagen crosslinks

Author and Year	AN Group N	AN Group M	AN Group SD	Comp. Group N	Comp. Group M	Comp. Group SD	SMD [95% CI]
Fazeli, 2015	20	0.8	0.3	26	0.81	0.3	−0.03 [−0.61, 0.54]
Ohwada, 2007	26	269.37	216.58	7	176	62.46	0.46 [−0.36, 1.29]
Estour, 2017	40	5743	3687	110	3899.29	2760.44	0.60 [0.24, 0.97]
Viapiana, 2007	55	0.78	0.43	15	0.5	0.25	0.69 [0.12, 1.27]
RE Model (Q = 4.05, df = 3, p = 0.256; I^2 = 29.2%, τ^2 = 0.03)							0.46 [0.13, 0.78]

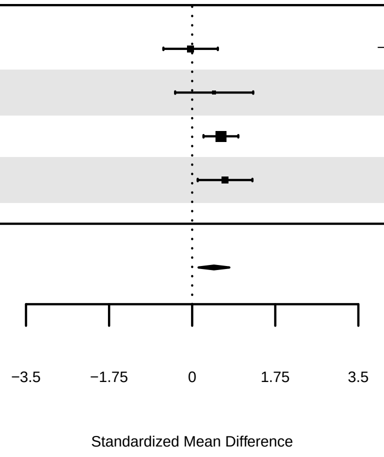

Cholecystokinin

Author and Year	AN Group N	AN Group M	AN Group SD	Comp. Group N	Comp. Group M	Comp. Group SD	SMD [95% CI]
Fernandez−Aranda, 2015	64	1.43	0.79	175	1.53	0.67	−0.14 [−0.43, 0.14]
Geracioti, 1992	6	0.9	0.8	6	0.6	0.2	0.47 [−0.59, 1.54]
Fujimoto, 1997	7	15.7	8.32	8	8.7	1.85	1.13 [0.10, 2.17]
RE Model (Q = 6.28, df = 2, p = 0.043; I^2 = 66.0%, τ^2 = 0.31)							0.36 [−0.42, 1.13]

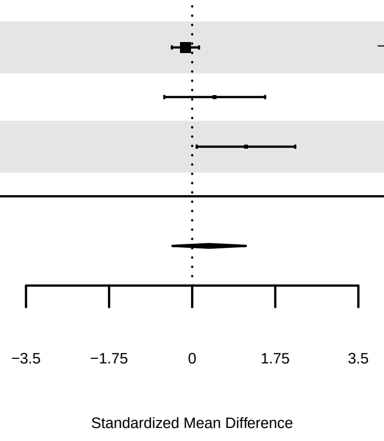

Cholesterol

Author and Year	AN Group N	AN Group M	AN Group SD	Comp. Group N	Comp. Group M	Comp. Group SD	SMD [95% CI]
Haluzi´K, 1999	17	5.42	1.2	17	5.42	0.98	0.00 [−0.66, 0.66]
Barja−Fernández, 2015	30	191.4	44.5	119	185.86	46.86	0.12 [−0.28, 0.52]
Terra, 2013	28	191.4	5.94	33	190.1	6.08	0.21 [−0.29, 0.71]
Cinkajzlova, 2017	22	7.66	14.2	15	4.36	0.74	0.29 [−0.35, 0.94]
Kaválková, 2012	18	7.64	12.78	16	4.32	0.73	0.35 [−0.32, 1.01]
Delporte, 2003	26	4.81	1.1	24	4.39	0.71	0.44 [−0.11, 1.00]
Haluzikova, 2009	19	5.2	1.31	16	4.3	1	0.75 [0.07, 1.42]
Amitani, 2013	9	202	25.48	9	179.78	24.18	0.85 [−0.07, 1.77]
Tanaka, 2004	34	5.9	1.63	9	4.6	0.56	0.86 [0.12, 1.60]
Case, 1997	9	5.7	0.76	10	4.14	0.47	2.39 [1.24, 3.54]
RE Model (Q = 18.98, df = 9, p = 0.025; I^2 = 42.9%, τ^2 = 0.08)							0.47 [0.20, 0.74]

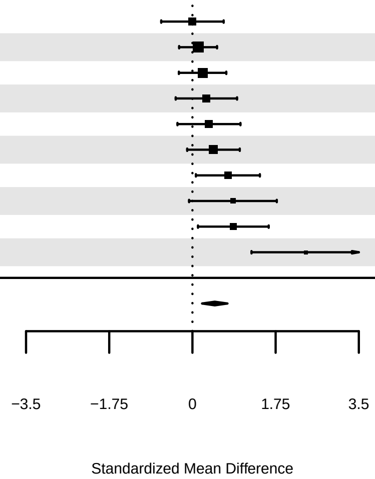

Cortisol

Author and Year	AN Group N	AN Group M	AN Group SD	Comp. Group N	Comp. Group M	Comp. Group SD	SMD [95% CI]
Nishita, 1989	4	7.5	2.07	5	21.7	11.6	−1.42 [−2.76, −0.08]
Hadigan, 1995	10	9.11	5.3	8	12.6	3.5	−0.72 [−1.64, 0.19]
Casper, 1988	7	14.9	11.18	14	14.5	1.8	0.06 [−0.81, 0.93]
Lanfranco, 2004	10	405	181.2	10	384.6	110.1	0.13 [−0.71, 0.97]
Laanfranco, 2004	10	405	181.2	10	384.6	110.1	0.13 [−0.71, 0.97]
Arimura, 2010	20	18.01	4.55	12	16.6	3	0.34 [−0.36, 1.04]
Estour, 2017	40	363	199	110	261.49	174.24	0.56 [0.19, 0.92]
Brambilla, 2003	22	125.8	58	20	94.9	47.6	0.57 [−0.04, 1.18]
Duclos, 1999	19	243.9	217.64	6	107.9	15.42	0.68 [−0.22, 1.59]
Jacoangeli, 2002	49	54	12.1	24	47	3	0.69 [0.19, 1.18]
Lawson, 2009	18	11.2	3.71	34	8.29	2.5	0.97 [0.37, 1.56]
Zumoff, 1983	14	11.3	3.84	10	7.3	2.58	1.14 [0.29, 1.99]
Schorr, 2015	18	0.74	0.43	42	0.36	0.24	1.22 [0.63, 1.80]
Ferrari, 1990	23	15.07	6.28	43	9.58	2.99	1.23 [0.69, 1.77]
Broglio, 2004	9	506	107.76	7	360.6	108.02	1.27 [0.24, 2.31]
Støving, 2002	13	402	197.45	10	192	36	1.34 [0.46, 2.22]
Monteleone, 2002	33	489	133	38	298	128	1.45 [0.93, 1.97]
Baskaran, 2016	13	11.3	3.3	24	6.85	1.85	1.78 [1.01, 2.56]
Mayer, 2005	29	20	4.9	15	11.59	3.5	1.84 [1.12, 2.57]
Miljic, 2006	9	744	169.42	10	425.8	117.3	2.11 [1.02, 3.20]
Germain, 2007	12	364	69.64	17	230.94	54.04	2.12 [1.22, 3.03]
Connan, 2007	18	331.13	89.88	15	165.96	47.14	2.19 [1.33, 3.04]
Galusca, 2015	23	453	93.8	36	263	75.66	2.26 [1.60, 2.91]
Stengel, 2013	8	994.4	292.35	32	475.63	139.07	2.86 [1.88, 3.85]
Mondelli, 2006	6	227	32.42	6	111	19.67	3.99 [2.08, 5.90]
RE Model (Q = 120.30, df = 24, p < 0.001; I^2 = 85.3%, τ^2 = 0.76)							1.11 [0.73, 1.49]

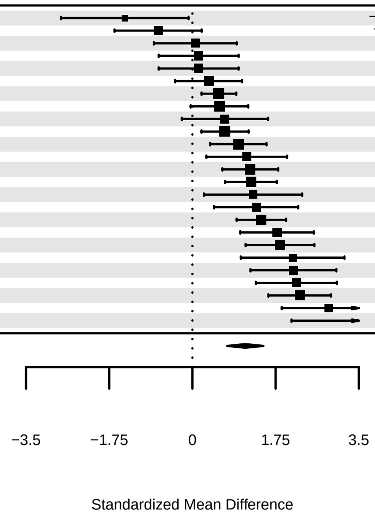

Creatinine

Author and Year	AN Group N	AN Group M	AN Group SD	Comp. Group N	Comp. Group M	Comp. Group SD	SMD [95% CI]
Rigaud, 2000	16	702	83	16	1283	162	−4.40 [−5.67, −3.13]
Johnston, 1984	6	678	98.38	12	1043	132.66	−2.83 [−4.14, −1.51]
Duclos, 1999	19	3.7	2.12	6	7	1.78	−1.56 [−2.54, −0.57]
Carlsson, 2017	20	63	9.8	78	69	9.8	−0.61 [−1.10, −0.11]
RE Model (Q = 35.76, df = 3, p < 0.001; I^2 = 91.4%, τ^2 = 2.45)							−2.28 [−3.90, −0.65]

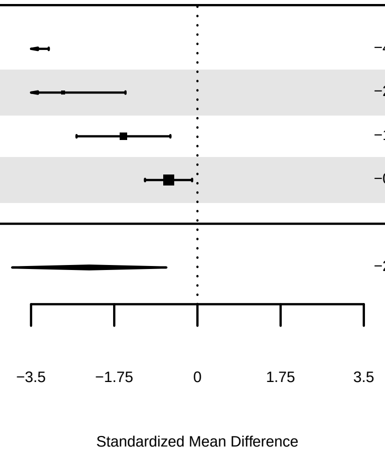

Des−acyl ghrelin

Author and Year	AN Group N	AN Group M	AN Group SD	Comp. Group N	Comp. Group M	Comp. Group SD	SMD [95% CI]
Uehara, 2011	9	1516.68	1400.24	9	677.52	477.21	0.76 [−0.15, 1.68]
Terashi, 2011	10	340.1	116.28	10	230.8	60.03	1.13 [0.22, 2.04]
Harada, 2008	10	340.1	116.28	10	230.8	60.03	1.13 [0.22, 2.04]
Nakahara, 2008	11	421.1	135.02	21	206.18	103.44	1.82 [0.98, 2.66]
RE Model (Q = 3.00, df = 3, p = 0.392; I^2 = 5.3%, τ^2 = 0.01)							1.24 [0.78, 1.70]

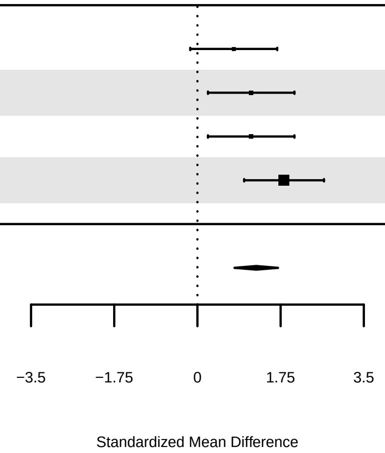

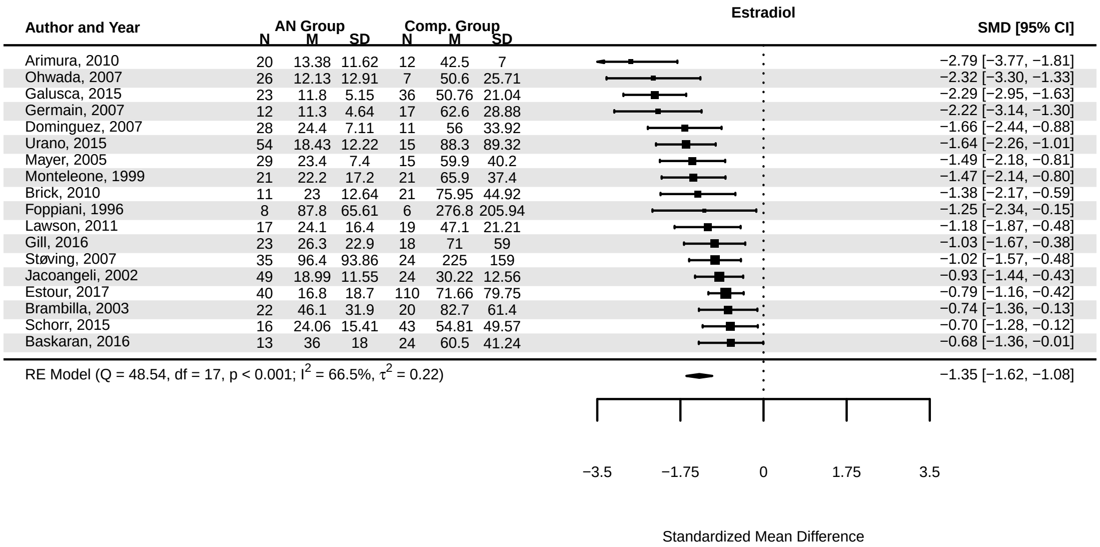
Estradiol
Author and Year
AN Group
N M SD
Comp. Group
N M SD
SMD [95% CI]
Arimura, 2010 20 13.38 11.62 12 42.5 7 −2.79 [−3.77, −1.81]
Ohwada, 2007 26 12.13 12.91 7 50.6 25.71 −2.32 [−3.30, −1.33]
Galusca, 2015 23 11.8 5.15 36 50.76 21.04 −2.29 [−2.95, −1.63]
Germain, 2007 12 11.3 4.64 17 62.6 28.88 −2.22 [−3.14, −1.30]
Dominguez, 2007 28 24.4 7.11 11 56 33.92 −1.66 [−2.44, −0.88]
Urano, 2015 54 18.43 12.22 15 88.3 89.32 −1.64 [−2.26, −1.01]
Mayer, 2005 29 23.4 7.4 15 59.9 40.2 −1.49 [−2.18, −0.81]
Monteleone, 1999 21 22.2 17.2 21 65.9 37.4 −1.47 [−2.14, −0.80]
Brick, 2010 11 23 12.64 21 75.95 44.92 −1.38 [−2.17, −0.59]
Foppiani, 1996 8 87.8 65.61 6 276.8 205.94 −1.25 [−2.34, −0.15]
Lawson, 2011 17 24.1 16.4 19 47.1 21.21 −1.18 [−1.87, −0.48]
Gill, 2016 23 26.3 22.9 18 71 59 −1.03 [−1.67, −0.38]
Støving, 2007 35 96.4 93.86 24 225 159 −1.02 [−1.57, −0.48]
Jacoangeli, 2002 49 18.99 11.55 24 30.22 12.56 −0.93 [−1.44, −0.43]
Estour, 2017 40 16.8 18.7 110 71.66 79.75 −0.79 [−1.16, −0.42]
Brambilla, 2003 22 46.1 31.9 20 82.7 61.4 −0.74 [−1.36, −0.13]
Schorr, 2015 16 24.06 15.41 43 54.81 49.57 −0.70 [−1.28, −0.12]
Baskaran, 2016 13 36 18 24 60.5 41.24 −0.68 [−1.36, −0.01]
RE Model (Q = 48.54, df = 17, p < 0.001; I2 = 66.5%, τ2 = 0.22) −1.35 [−1.62, −1.08]
−3.5 −1.75 0 1.75 3.5
Standardized Mean Difference

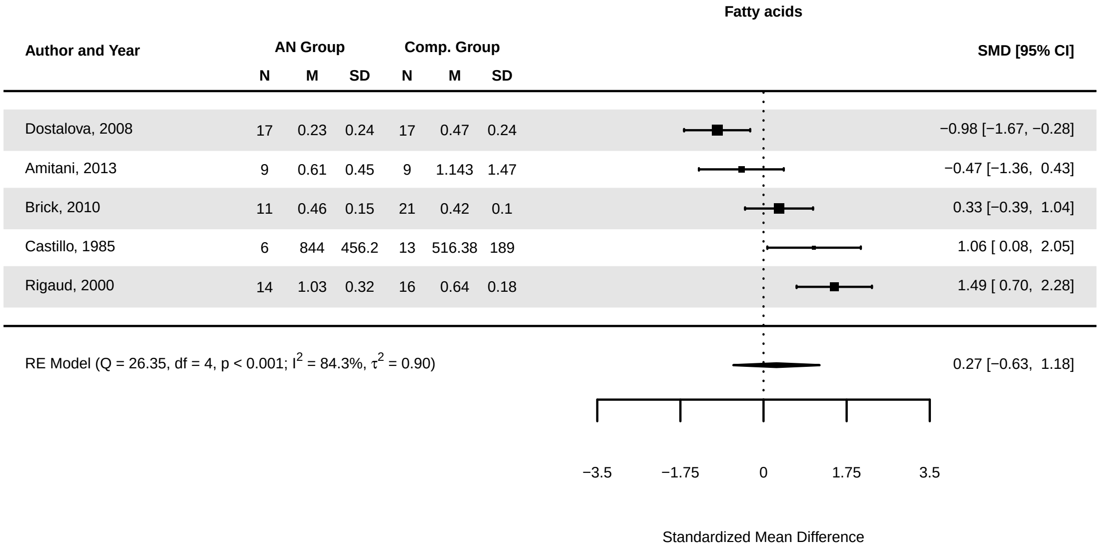
Fatty acids
Author and Year
AN Group
N M SD
Comp. Group
N M SD
SMD [95% CI]
Dostalova, 2008 17 0.23 0.24 17 0.47 0.24 −0.98 [−1.67, −0.28]
Amitani, 2013 9 0.61 0.45 9 1.143 1.47 −0.47 [−1.36, 0.43]
Brick, 2010 11 0.46 0.15 21 0.42 0.1 0.33 [−0.39, 1.04]
Castillo, 1985 6 844 456.2 13 516.38 189 1.06 [0.08, 2.05]
Rigaud, 2000 14 1.03 0.32 16 0.64 0.18 1.49 [0.70, 2.28]
RE Model (Q = 26.35, df = 4, p < 0.001; I2 = 84.3%, τ2 = 0.90) 0.27 [−0.63, 1.18]
−3.5 −1.75 0 1.75 3.5
Standardized Mean Difference

Follicle stimulating hormone

Author and Year	AN Group N	AN Group M	AN Group SD	Comp. Group N	Comp. Group M	Comp. Group SD	SMD [95% CI]
Germain, 2007	12	3	1.98	17	6.48	2.71	−1.39 [−2.19, −0.58]
Støving, 2007	35	2.8	2.5	24	5.5	1.7	−1.20 [−1.76, −0.65]
Dominguez, 2007	28	1.8	2.15	11	3.7	1.34	−0.95 [−1.66, −0.23]
Arimura, 2010	20	3.56	3.4	12	5.43	1.1	−0.65 [−1.37, 0.06]
Estour, 2017	40	3.8	2.9	110	5.6036	3.6446	−0.52 [−0.88, −0.15]
Popovic, 2004	20	3.9	3.92	19	4.9	3.39	−0.27 [−0.88, 0.35]
Brambilla, 2003	22	6.1	2.6	20	5	1.9	0.47 [−0.13, 1.07]
RE Model ($Q = 22.54$, $df = 6$, $p < 0.001$; $I^2 = 75.6\%$, $\tau^2 = 0.28$)							−0.62 [−1.08, −0.17]

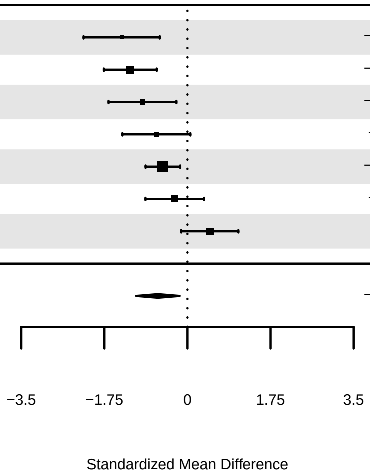

Free thyroxine

Author and Year	AN Group N	AN Group M	AN Group SD	Comp. Group N	Comp. Group M	Comp. Group SD	SMD [95% CI]
Nogueira, 2013	11	12.7	0.44	10	13.9	0.45	−2.59 [−3.73, −1.45]
Fukushima, 1993	8	0.92	0.13	16	1.34	0.19	−2.34 [−3.40, −1.29]
Arimura, 2010	20	0.94	0.13	12	1.24	0.14	−2.19 [−3.07, −1.31]
Rigaud, 2000	14	7.2	2.4	16	10.6	0.4	−1.99 [−2.85, −1.13]
Onur, 2005	28	13.4	1.4	49	16.7	2.7	−1.41 [−1.92, −0.90]
Dostalova, 2008	17	12.3	1.8	17	14.6	2.36	−1.07 [−1.77, −0.37]
Estour, 2017	40	14.1	2.5	110	16.4	2.54	−0.90 [−1.28, −0.53]
Kaválková, 2012	18	12.35	2.1	16	14.19	1.93	−0.89 [−1.58, −0.20]
Jacoangeli, 2002	49	11.18	1.2	24	12.98	3.1	−0.88 [−1.38, −0.38]
Barja−Fernández, 2015	30	0.9	0.1	119	1.02	0.15	−0.84 [−1.25, −0.43]
Monteleone, 2005	27	8.7	1.3	24	9.3	1.9	−0.37 [−0.91, 0.18]
Brambilla, 2003	22	1.2	0.4	20	1.3	0.2	−0.31 [−0.90, 0.29]
RE Model ($Q = 39.78$, $df = 11$, $p < 0.001$; $I^2 = 79.4\%$, $\tau^2 = 0.35$)							−1.21 [−1.60, −0.82]

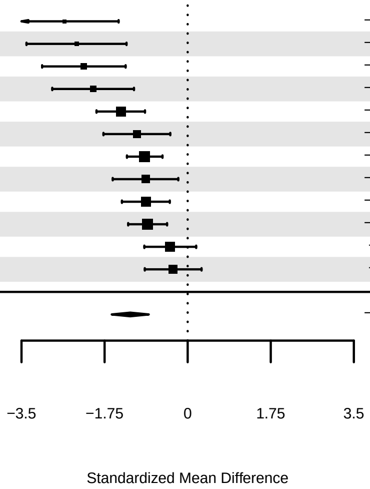

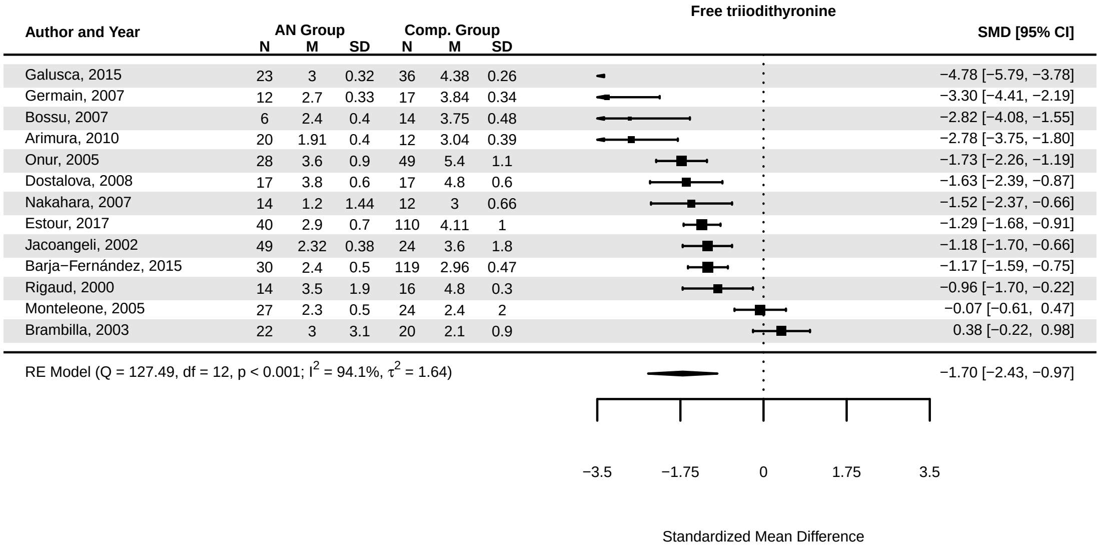
Free triiodithyronine
Author and Year
AN Group
Comp. Group
N M SD N M SD
SMD [95% CI]
Galusca, 2015 23 3 0.32 36 4.38 0.26 −4.78 [−5.79, −3.78]
Germain, 2007 12 2.7 0.33 17 3.84 0.34 −3.30 [−4.41, −2.19]
Bossu, 2007 6 2.4 0.4 14 3.75 0.48 −2.82 [−4.08, −1.55]
Arimura, 2010 20 1.91 0.4 12 3.04 0.39 −2.78 [−3.75, −1.80]
Onur, 2005 28 3.6 0.9 49 5.4 1.1 −1.73 [−2.26, −1.19]
Dostalova, 2008 17 3.8 0.6 17 4.8 0.6 −1.63 [−2.39, −0.87]
Nakahara, 2007 14 1.2 1.44 12 3 0.66 −1.52 [−2.37, −0.66]
Estour, 2017 40 2.9 0.7 110 4.11 1 −1.29 [−1.68, −0.91]
Jacoangeli, 2002 49 2.32 0.38 24 3.6 1.8 −1.18 [−1.70, −0.66]
Barja−Fernández, 2015 30 2.4 0.5 119 2.96 0.47 −1.17 [−1.59, −0.75]
Rigaud, 2000 14 3.5 1.9 16 4.8 0.3 −0.96 [−1.70, −0.22]
Monteleone, 2005 27 2.3 0.5 24 2.4 2 −0.07 [−0.61, 0.47]
Brambilla, 2003 22 3 3.1 20 2.1 0.9 0.38 [−0.22, 0.98]
RE Model (Q = 127.49, df = 12, p < 0.001; I² = 94.1%, τ² = 1.64) −1.70 [−2.43, −0.97]
−3.5 −1.75 0 1.75 3.5
Standardized Mean Difference

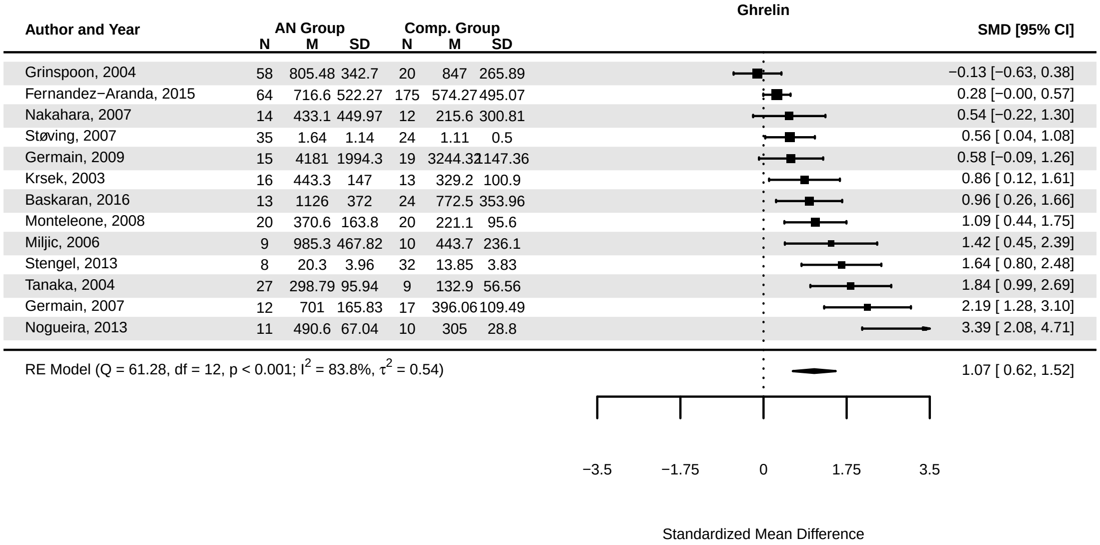
Ghrelin
Author and Year
AN Group
Comp. Group
N M SD N M SD
SMD [95% CI]
Grinspoon, 2004 58 805.48 342.7 20 847 265.89 −0.13 [−0.63, 0.38]
Fernandez−Aranda, 2015 64 716.6 522.27 175 574.27 495.07 0.28 [−0.00, 0.57]
Nakahara, 2007 14 433.1 449.97 12 215.6 300.81 0.54 [−0.22, 1.30]
Støving, 2007 35 1.64 1.14 24 1.11 0.5 0.56 [0.04, 1.08]
Germain, 2009 15 4181 1994.3 19 3244.32 1147.36 0.58 [−0.09, 1.26]
Krsek, 2003 16 443.3 147 13 329.2 100.9 0.86 [0.12, 1.61]
Baskaran, 2016 13 1126 372 24 772.5 353.96 0.96 [0.26, 1.66]
Monteleone, 2008 20 370.6 163.8 20 221.1 95.6 1.09 [0.44, 1.75]
Miljic, 2006 9 985.3 467.82 10 443.7 236.1 1.42 [0.45, 2.39]
Stengel, 2013 8 20.3 3.96 32 13.85 3.83 1.64 [0.80, 2.48]
Tanaka, 2004 27 298.79 95.94 9 132.9 56.56 1.84 [0.99, 2.69]
Germain, 2007 12 701 165.83 17 396.06 109.49 2.19 [1.28, 3.10]
Nogueira, 2013 11 490.6 67.04 10 305 28.8 3.39 [2.08, 4.71]
RE Model (Q = 61.28, df = 12, p < 0.001; I² = 83.8%, τ² = 0.54) 1.07 [0.62, 1.52]
−3.5 −1.75 0 1.75 3.5
Standardized Mean Difference

Glucose

Author and Year	AN Group N	AN Group M	AN Group SD	Comp. Group N	Comp. Group M	Comp. Group SD	SMD [95% CI]
Nogueira, 2013	11	3.9	0.15	10	4.5	0.12	−4.22 [−5.73, −2.70]
Amitani, 2013	9	71.98	2.88	9	85.22	4.04	−3.59 [−5.06, −2.13]
Fukushima, 1993	8	75.5	5.55	16	87.1	6.58	−1.79 [−2.75, −0.82]
Zuniga−Guajardo, 1986	9	84.3	4.24	7	91.5	4.16	−1.62 [−2.71, −0.53]
Tamia, 1991	19	4	0.42	8	4.6	0.26	−1.52 [−2.42, −0.62]
Kinzig, 2007	13	75.56	5.54	13	85.1	6.92	−1.47 [−2.32, −0.63]
Tanaka, 2004	34	4.14	0.4	9	4.7	0.28	−1.45 [−2.23, −0.66]
Tagami, 1991	31	4.18	0.67	25	5.09	0.58	−1.42 [−2.00, −0.84]
Kaválková, 2012	18	3.97	0.28	16	4.38	0.3	−1.38 [−2.12, −0.65]
Rigaud, 2000	14	4.3	0.5	16	4.8	0.2	−1.31 [−2.08, −0.54]
Cinkajzlova, 2017	22	4.02	0.27	15	4.39	0.29	−1.30 [−2.01, −0.59]
Barja−Fernández, 2015	30	70.1	16.4	119	95.12	20.19	−1.28 [−1.70, −0.85]
Miljic, 2006	9	3.4	0.56	10	4.1	0.6	−1.15 [−2.08, −0.22]
Dostalova, 2009	10	4.2	0.36	20	4.7	0.52	−1.03 [−1.81, −0.24]
Broglio, 2004	9	3.4	0.28	7	3.8	0.48	−1.00 [−1.99, −0.00]
Stengel, 2013	8	71.6	14.55	32	104.65	38.42	−0.92 [−1.70, −0.13]
Delporte, 2003	26	4.3	0.5	24	4.7	0.47	−0.81 [−1.38, −0.24]
Monteleone, 2000b	21	82.7	11.5	15	93.6	20.4	−0.68 [−1.34, −0.01]
Castillo, 1985	6	62.5	11.18	13	67.3	6.92	−0.55 [−1.49, 0.39]
Nakai, 2001	17	29.9	9.2	11	35.2	9.8	−0.55 [−1.30, 0.20]
Haluzikova, 2009	19	3.8	0.25	16	4.1	0.96	−0.44 [−1.09, 0.22]
Dostalova, 2008	17	3.8	0.24	17	4.1	1	−0.40 [−1.07, 0.26]
Nakahara, 2007	14	4.1	1.8	12	4.7	1.32	−0.36 [−1.12, 0.39]
Støving, 2007	35	4.89	2.16	24	5.1	0.5	−0.12 [−0.64, 0.39]
RE Model (Q = 66.49, df = 23, p < 0.001; I^2 = 65.2%, τ^2 = 0.26)							−1.12 [−1.38, −0.86]

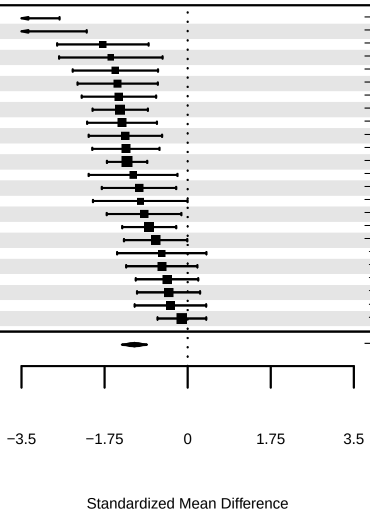

Glycerol

Author and Year	AN Group N	AN Group M	AN Group SD	Comp. Group N	Comp. Group M	Comp. Group SD	SMD [95% CI]
Bartak, 2004	10	159.3	86.7	10	191.9	77.7	−0.38 [−1.23, 0.47]
Zuniga−Guajardo, 1986	9	52.5	11.87	7	56.6	9.55	−0.35 [−1.30, 0.59]
Castillo, 1985	6	143.1	70.7	13	142.09	49.79	0.02 [−0.91, 0.94]
Dostalova, 2007	10	139.9	57.24	15	135.8	65.14	0.06 [−0.71, 0.84]
Nedvidkova, 2004	5	141.9	17.8	6	96.3	8.72	3.08 [1.40, 4.77]
RE Model (Q = 14.15, df = 4, p = 0.007; I^2 = 82.5%, τ^2 = 1.11)							0.33 [−0.71, 1.36]

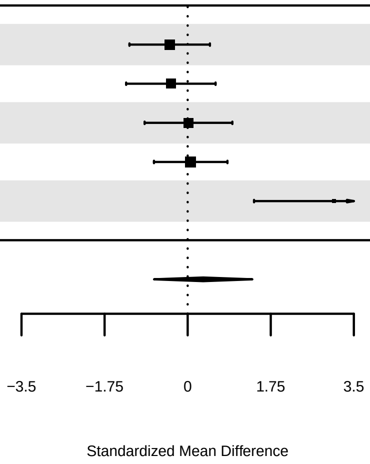

Growth Hormone

Author and Year	AN Group N	AN Group M	AN Group SD	Comp. Group N	Comp. Group M	Comp. Group SD	SMD [95% CI]
Arimura, 2010	20	5.42	4.48	12	9.4	9.9	−0.56 [−1.27, 0.15]
Estour, 2017	40	7.9	12.2	110	6.14	18.51	0.10 [−0.26, 0.46]
Grinspoon, 2004	58	4.25	4.32	20	2.3	2.2	0.50 [−0.01, 1.00]
Tanaka, 2004	34	13.89	29.16	9	0.6	0.3	0.50 [−0.23, 1.23]
Germain, 2009	15	11.6	15.7	19	4.75	1.71	0.64 [−0.04, 1.32]
Counts, 1992	11	14	24.4	6	0.5	0.1	0.64 [−0.33, 1.61]
Gianotti, 2000	9	6.8	9.1	8	1.6	0.8	0.74 [−0.20, 1.68]
Galusca, 2015	23	12.2	7.5	14	6.6	3.6	0.86 [0.19, 1.54]
Krsek, 2003	16	4.9	2.8	13	2.6	1.3	0.99 [0.23, 1.74]
Miljic, 2006	9	8.5	10.5	10	0.6	0.6	1.05 [0.12, 1.97]
Mondelli, 2006	6	9.5	6.7	6	2.8	3.4	1.16 [0.02, 2.31]
Ferrari, 1990	23	5.53	3.6	43	2.11	1.74	1.33 [0.78, 1.88]
RE Model (Q = 27.65, df = 11, p = 0.004; I^2 = 59.0%, τ^2 = 0.16)							0.62 [0.31, 0.93]

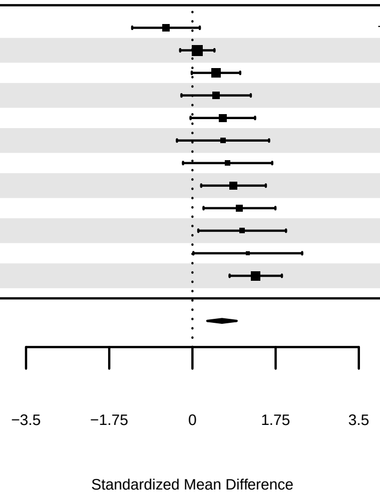

High−density lipoprotein cholesterol (HDL)

Author and Year	AN Group N	AN Group M	AN Group SD	Comp. Group N	Comp. Group M	Comp. Group SD	SMD [95% CI]
Kaválková, 2012	18	1.57	0.41	16	1.76	0.34	−0.49 [−1.16, 0.18]
Cinkajzlova, 2017	22	1.59	0.45	15	1.76	0.37	−0.40 [−1.04, 0.25]
Amitani, 2013	9	66.78	9.13	9	68.22	6.92	−0.17 [−1.05, 0.71]
Delporte, 2003	26	1.45	0.35	24	1.42	0.28	0.09 [−0.45, 0.64]
Barja−Fernández, 2015	30	71.7	16.4	119	54.39	15.26	1.11 [0.69, 1.53]
Lawson, 2007	140	69.96	17.11	41	47.98	14.51	1.32 [0.95, 1.69]
Terra, 2013	28	78.8	6.43	33	67.9	2.46	2.28 [1.64, 2.93]
RE Model (Q = 68.70, df = 6, p < 0.001; I^2 = 92.7%, τ^2 = 0.99)							0.56 [−0.22, 1.33]

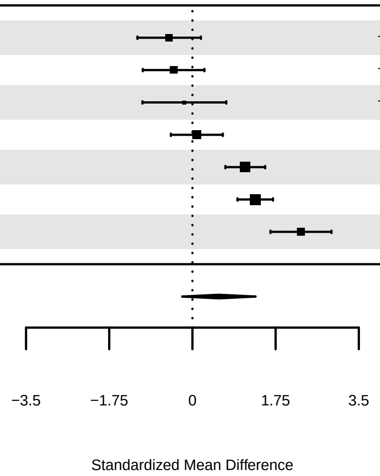

Insulin

Author and Year	AN Group N	AN Group M	AN Group SD	Comp. Group N	Comp. Group M	Comp. Group SD	SMD [95% CI]
Nogueira, 2013	11	3	0.75	10	7.7	1.47	−3.93 [−5.37, −2.48]
Nakahara, 2008	11	19.9	6	21	51.25	21.42	−1.71 [−2.54, −0.89]
Fukushima, 1993	8	3.6	1.05	16	6.3	1.93	−1.53 [−2.46, −0.61]
Amitani, 2013	9	3.01	1.38	9	5.87	2.37	−1.40 [−2.40, −0.41]
Dostalova, 2009	10	2.2	0.87	20	6.9	4.05	−1.36 [−2.17, −0.54]
Baskaran, 2016	13	2.6	1.4	24	6.15	3.07	−1.33 [−2.05, −0.60]
Broglio, 2004	9	8.1	2.26	7	13.2	5.14	−1.28 [−2.31, −0.24]
Støving, 2002	13	21	13.85	10	41	18	−1.22 [−2.09, −0.35]
Rigaud, 2000	14	26	3	16	29	2	−1.16 [−1.92, −0.40]
Stengel, 2013	8	2.1	0.79	32	13.25	11.31	−1.07 [−1.86, −0.27]
Tagami, 2004	31	5	5.6	25	14.46	13.78	−0.92 [−1.47, −0.38]
Kaválková, 2012	18	15.79	3.09	16	19.29	4.33	−0.92 [−1.61, −0.23]
Cinkajzlova, 2017	22	15.8	3.62	15	19.3	4.19	−0.89 [−1.56, −0.21]
Brick, 2010	11	5.3	2.52	21	8.72	4.7	−0.81 [−1.55, −0.07]
Barja−Fernández, 2015	30	6.8	6.4	119	13.82	12.21	−0.62 [−1.02, −0.21]
Rigaud, 2007	15	5.9	1.1	15	6.5	1	−0.56 [−1.27, 0.16]
Nakahara, 2007	14	17.4	41.82	12	42.1	57.7	−0.48 [−1.24, 0.28]
Haluzikova, 2009	19	12	4.2	16	14.3	5.42	−0.47 [−1.13, 0.19]
Dostalova, 2008	17	12	3.96	17	14.3	5.6	−0.46 [−1.13, 0.20]
Støving, 2007	27	27.26	19.06	24	35	14	−0.45 [−1.00, 0.10]
Delporte, 2003	26	67	55	24	73	43.16	−0.12 [−0.67, 0.43]
Kinzig, 2007	13	4.7	2.77	13	4.5	2.07	0.08 [−0.67, 0.82]
Gianotto, 2000	9	7.3	7.07	8	6.8	1.58	0.09 [−0.81, 0.99]
Miljic, 2006	9	11.85	6.5	10	8.1	9.6	0.43 [−0.44, 1.30]
Terra, 2013	28	10.4	2.5	33	8.2	0.8	1.21 [0.67, 1.75]
RE Model (Q = 108.63, df = 24, p < 0.001; I^2 = 79.4%, τ^2 = 0.49)							−0.76 [−1.08, −0.45]

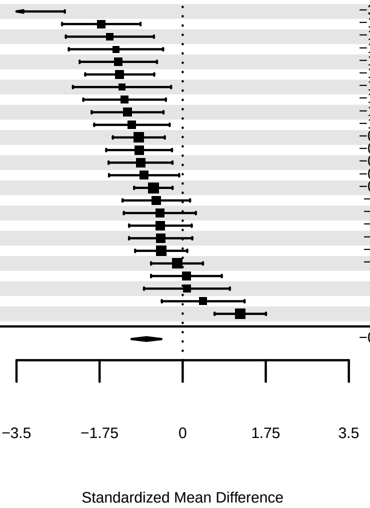

Insulin−like growth factor 1

Author and Year	AN Group N	AN Group M	AN Group SD	Comp. Group N	Comp. Group M	Comp. Group SD	SMD [95% CI]
Galusca, 2015	23	128	28.14	36	264.83	47.86	−3.26 [−4.05, −2.48]
Jacoangeli, 2002	49	198	14	24	254	27	−2.89 [−3.56, −2.21]
Gianotto, 2000	9	96.6	62.22	8	274.4	66.93	−2.62 [−3.88, −1.36]
Foppiani, 1996	8	15.6	7.93	6	30.4	3.57	−2.14 [−3.41, −0.87]
Arimura, 2010	20	150.3	90.4	12	300	57	−1.83 [−2.66, −1.00]
Germain, 2009	15	151	71.09	19	288.68	77.12	−1.80 [−2.59, −1.02]
Germain, 2007	12	163	53.06	17	290.06	82.4	−1.72 [−2.56, −0.87]
Ohwada, 2007	26	126.15	75.83	7	325	208.2	−1.70 [−2.61, −0.79]
Saito, 2007	33	114.2	72.4	10	235.8	94.2	−1.54 [−2.30, −0.77]
Dostalova, 2008	17	161.6	43.64	17	217.5	37.84	−1.34 [−2.07, −0.61]
Grindpoon, 1996	22	202	93	23	310	72	−1.28 [−1.91, −0.65]
Fazeli, 2010	20	146.4	48.38	10	203.5	51	−1.13 [−1.92, −0.34]
Bossu, 2007	6	168	62	14	249.5	79.37	−1.04 [−2.01, −0.07]
Estour, 2017	40	157	61	110	238.51	86.82	−1.00 [−1.38, −0.63]
Cassanueva, 1997	10	157.4	86.1	18	228	60.19	−0.98 [−1.77, −0.18]
Urano, 2015	54	118.26	70.07	15	176	40.99	−0.88 [−1.46, −0.29]
Krsek, 2003	16	132	79.4	13	202.5	99.2	−0.77 [−1.51, −0.03]
Baskaran, 2016	13	174	73	24	238.5	88.65	−0.75 [−1.44, −0.07]
Lawson, 2009	18	187.5	72.15	24	236.73	79.29	−0.63 [−1.25, −0.02]
Schorr, 2017	16	157.97	89.45	43	203.9	104.38	−0.45 [−1.02, 0.12]
Lawson, 2007	140	325.64	149.17	41	355.85	134.44	−0.21 [−0.55, 0.14]
Grinspoon, 2004	58	28.08	10.79	20	22.6	8.71	0.53 [0.02, 1.04]
RE Model (Q = 148.56, df = 21, p < 0.001; I^2 = 85.5%, τ^2 = 0.66)							−1.28 [−1.66, −0.91]

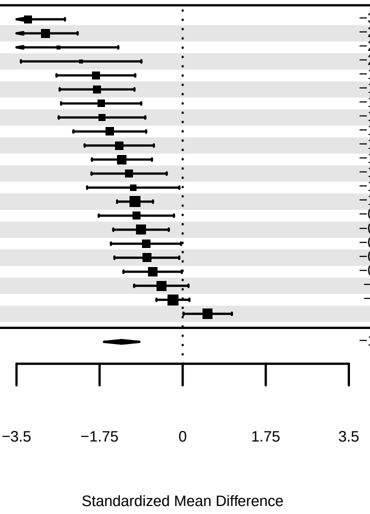

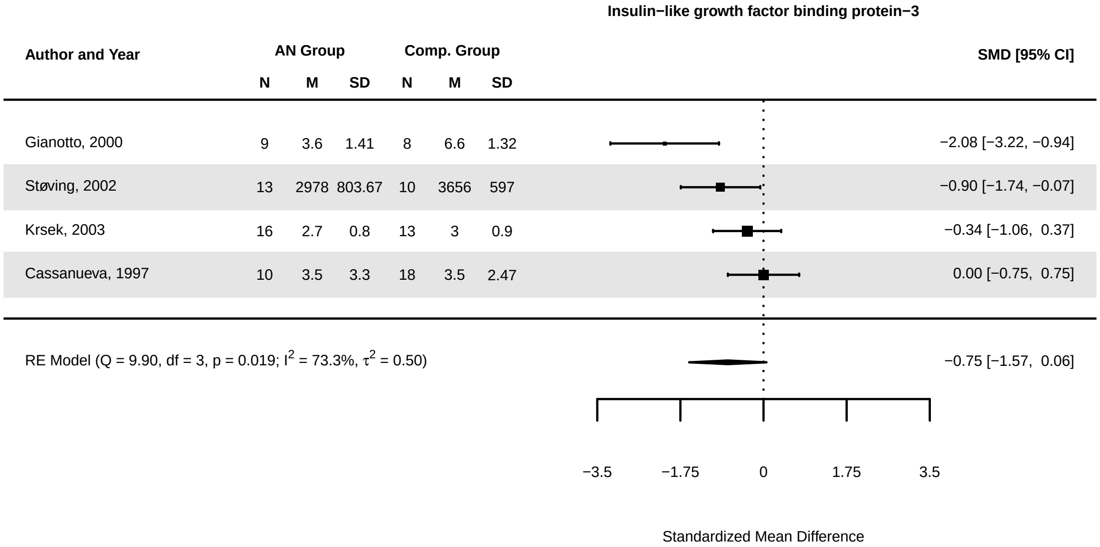
Insulin−like growth factor binding protein−3
Author and Year
AN Group
Comp. Group
N M SD N M SD
SMD [95% CI]
Gianotto, 2000 9 3.6 1.41 8 6.6 1.32 −2.08 [−3.22, −0.94]
Støving, 2002 13 2978 803.67 10 3656 597 −0.90 [−1.74, −0.07]
Krsek, 2003 16 2.7 0.8 13 3 0.9 −0.34 [−1.06, 0.37]
Cassanueva, 1997 10 3.5 3.3 18 3.5 2.47 0.00 [−0.75, 0.75]
RE Model (Q = 9.90, df = 3, p = 0.019; I2 = 73.3%, τ2 = 0.50) −0.75 [−1.57, 0.06]
−3.5 −1.75 0 1.75 3.5
Standardized Mean Difference

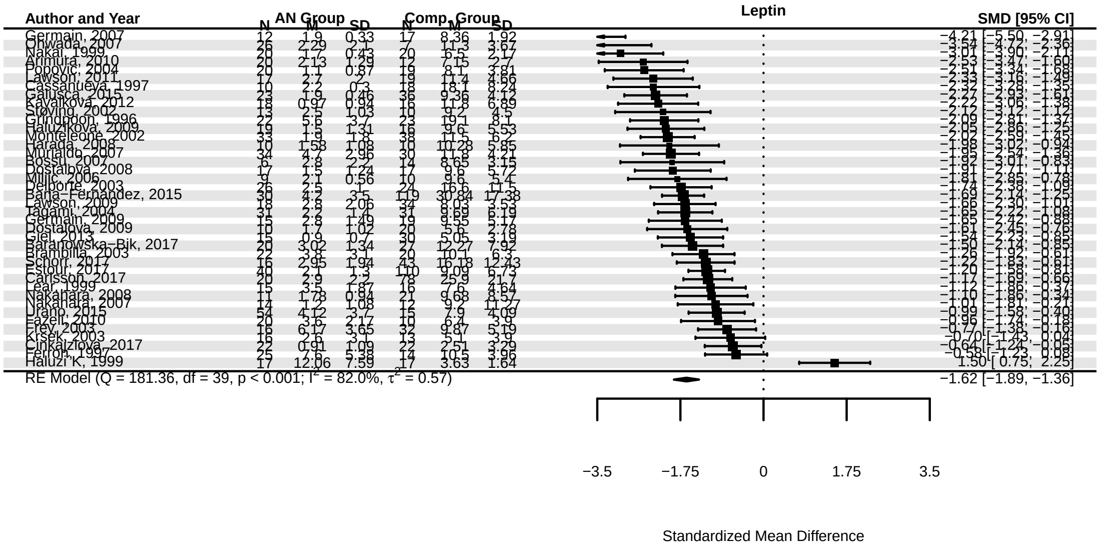
Leptin
Author and Year
AN Group
Comp. Group
N M SD N M SD
SMD [95% CI]
Germain, 2007 12 1.9 0.33 17 8.36 1.92 −4.21 [−5.50, −2.91]
Ohwada, 2007 26 2.29 2.1 7 11.3 3.67 −3.54 [−4.72, −2.36]
Nakai, 1999 20 1.7 0.43 20 6.5 2.17 −3.01 [−3.90, −2.11]
Arimura, 2010 20 2.13 1.29 12 7.15 2.7 −2.53 [−3.47, −1.60]
Popovic, 2004 20 1.1 0.87 19 8.1 3.81 −2.51 [−3.34, −1.68]
Lawson, 2011 17 2.7 2 19 11.4 4.66 −2.33 [−3.16, −1.49]
Cassanueva, 1997 10 2.2 0.3 18 18.1 8.24 −2.32 [−3.28, −1.35]
Galusca, 2015 23 1.9 0.46 36 9.36 4.12 −2.27 [−2.93, −1.61]
Kavalkova, 2012 18 0.97 0.94 16 11.8 6.89 −2.22 [−3.06, −1.38]
Støving, 2002 13 2.5 1.03 10 9.2 4.5 −2.12 [−3.12, −1.12]
Grinspoon, 1996 22 5.6 3.7 23 19.1 8.1 −2.09 [−2.81, −1.37]
Haluzíková, 2009 19 1.5 1.31 16 9.6 5.53 −2.05 [−2.86, −1.25]
Monteleone, 2002 33 1.9 1.8 38 11.5 6.2 −2.02 [−2.59, −1.45]
Harada, 2008 10 1.58 1.08 10 10.28 5.85 −1.98 [−3.02, −0.94]
Murialdo, 2007 34 4.7 2.96 30 11.8 4.21 −1.95 [−2.54, −1.36]
Bossu, 2007 6 2.8 2.2 14 8.65 3.15 −1.92 [−3.01, −0.83]
Dostalova, 2008 17 1.5 1.24 17 9.6 5.72 −1.91 [−2.71, −1.11]
Miljic, 2006 9 2.1 0.56 10 9.6 5.4 −1.81 [−2.85, −0.78]
Delporte, 2003 26 2.5 1 24 16.6 11.5 −1.74 [−2.38, −1.09]
Baña−Fernández, 2015 30 4.2 3.5 119 30.84 17.38 −1.69 [−2.14, −1.25]
Lawson, 2009 18 2.8 2.06 34 8.03 3.53 −1.66 [−2.30, −1.01]
Tagami, 2004 31 2.2 1.4 31 9.69 6.19 −1.65 [−2.22, −1.08]
Germain, 2009 15 2.8 1.49 19 9.55 5.17 −1.65 [−2.42, −0.88]
Dostalova, 2009 10 1.7 1.02 20 5.6 2.78 −1.61 [−2.45, −0.76]
Giel, 2013 15 0.9 0.7 30 5.05 3.19 −1.54 [−2.23, −0.85]
Baranowska−Bik, 2017 20 3.02 1.34 27 12.27 7.92 −1.50 [−2.14, −0.85]
Brambilla, 2003 22 3.8 3.1 20 10.1 6.3 −1.26 [−1.92, −0.61]
Schorr, 2017 16 2.95 1.94 43 16.18 12.43 −1.22 [−1.83, −0.61]
Estour, 2017 40 2.1 1.3 110 9.09 6.73 −1.20 [−1.58, −0.81]
Carlsson, 2017 20 2.9 2.2 78 25.9 21.7 −1.17 [−1.69, −0.66]
Lear, 1999 15 3.5 1.87 16 7.6 4.64 −1.12 [−1.86, −0.37]
Nakahara, 2008 11 1.78 0.94 21 9.68 8.57 −1.10 [−1.86, −0.34]
Nakahara, 2007 14 1.2 1.08 12 9.2 11.27 −1.01 [−1.81, −0.21]
Urano, 2015 54 4.12 3.7 15 7.9 4.09 −0.99 [−1.58, −0.40]
Fazeli, 2010 20 3.6 2.17 10 6.4 3.9 −0.96 [−1.74, −0.18]
Frey, 2003 16 6.17 3.65 32 9.87 5.19 −0.77 [−1.38, −0.16]
Krsek, 2003 16 2.6 3.1 13 5.1 3.9 −0.70 [−1.43, 0.04]
Cinkajzlova, 2017 22 0.91 1.09 22 2.51 3.29 −0.64 [−1.24, −0.05]
Ferron, 1997 25 7.6 5.38 14 10.5 3.96 −0.58 [−1.23, 0.08]
Haluzík, 1999 17 12.06 7.59 17 3.63 1.64 1.50 [0.75, 2.25]
RE Model (Q = 181.36, df = 39, p < 0.001; I2 = 82.0%, τ2 = 0.57) −1.62 [−1.89, −1.36]
−3.5 −1.75 0 1.75 3.5
Standardized Mean Difference

Low−density lipoprotein cholesterol (LDL)

Author and Year	AN Group N	AN Group M	AN Group SD	Comp. Group N	Comp. Group M	Comp. Group SD	SMD [95% CI]
Lawson, 2007	140	91.07	25.87	41	96.73	21.71	−0.23 [−0.57, 0.12]
Barja−Fernández, 2015	30	103.5	35.3	119	108.61	41.23	−0.13 [−0.53, 0.27]
Cinkajzlova, 2017	22	2.35	0.77	15	2.18	0.48	0.25 [−0.40, 0.89]
Delporte, 2003	26	2.95	0.9	24	2.67	0.76	0.33 [−0.22, 0.88]
Kaválková, 2012	18	2.37	0.7	16	2.15	0.5	0.35 [−0.31, 1.01]
Amitani, 2013	9	105.89	22.68	9	95.44	20.11	0.46 [−0.43, 1.36]
Terra, 2013	28	113.8	5.21	33	105.7	4.25	1.70 [1.12, 2.28]
RE Model (Q = 34.58, df = 6, p < 0.001; I^2 = 82.5%, τ^2 = 0.34)							0.37 [−0.12, 0.85]

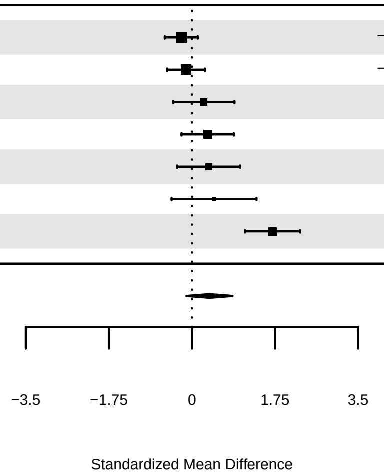

Luteining hormone

Author and Year	AN Group N	AN Group M	AN Group SD	Comp. Group N	Comp. Group M	Comp. Group SD	SMD [95% CI]
Arimura, 2010	20	0.5	0.72	12	5.5	2	−3.64 [−4.77, −2.51]
Germain, 2007	12	1	0.7	17	10.8059	7.8989	−1.56 [−2.39, −0.74]
Støving, 2007	35	2.0714	3.4415	24	5.1	1.7	−1.04 [−1.59, −0.50]
Estour, 2017	40	1.6	2.2	110	7.8545	8.3754	−0.86 [−1.23, −0.48]
Dominguez, 2007	28	1.5	3.18	11	2.7	2.95	−0.38 [−1.07, 0.31]
Popovic, 2004	20	1	4.4	19	3.1	8.9	−0.30 [−0.91, 0.32]
RE Model (Q = 31.10, df = 5, p < 0.001; I^2 = 91.9%, τ^2 = 1.13)							−1.23 [−2.12, −0.33]

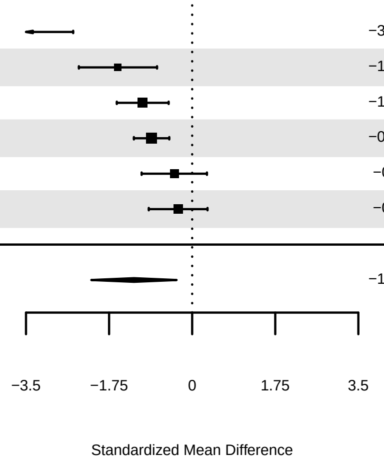

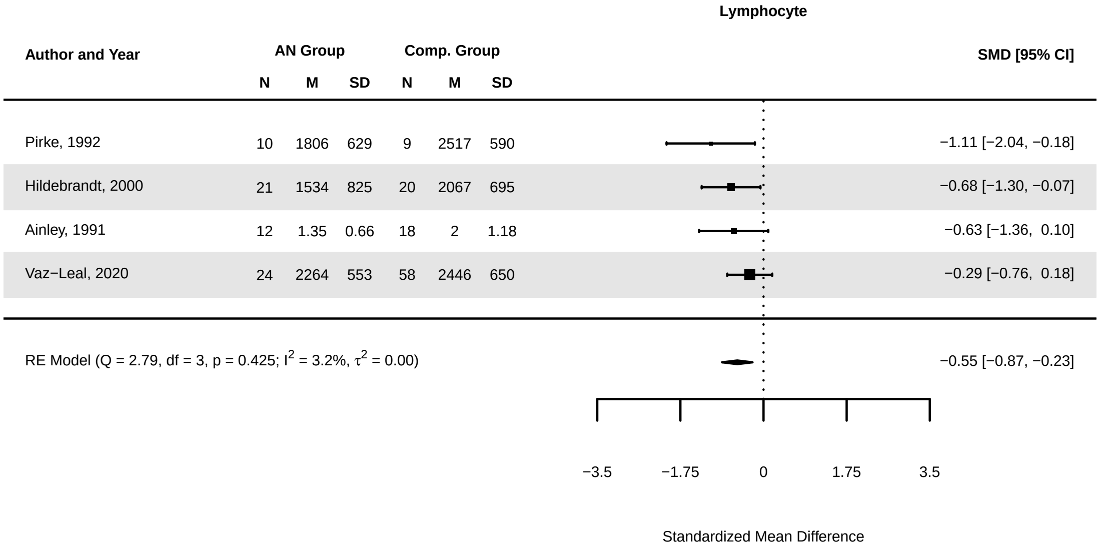
Lymphocyte
Author and Year
AN Group
Comp. Group
SMD [95% CI]
N M SD N M SD
Pirke, 1992 10 1806 629 9 2517 590 −1.11 [−2.04, −0.18]
Hildebrandt, 2000 21 1534 825 20 2067 695 −0.68 [−1.30, −0.07]
Ainley, 1991 12 1.35 0.66 18 2 1.18 −0.63 [−1.36, 0.10]
Vaz−Leal, 2020 24 2264 553 58 2446 650 −0.29 [−0.76, 0.18]
RE Model (Q = 2.79, df = 3, p = 0.425; I² = 3.2%, τ² = 0.00) −0.55 [−0.87, −0.23]
−3.5
−1.75
0
1.75
3.5
Standardized Mean Difference

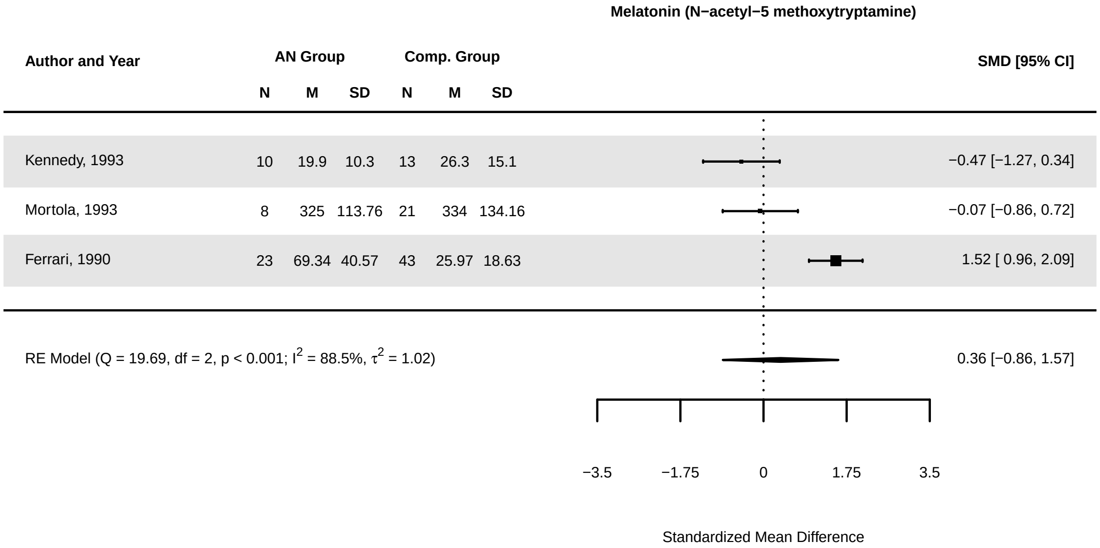
Melatonin (N−acetyl−5 methoxytryptamine)
Author and Year
AN Group
Comp. Group
SMD [95% CI]
N M SD N M SD
Kennedy, 1993 10 19.9 10.3 13 26.3 15.1 −0.47 [−1.27, 0.34]
Mortola, 1993 8 325 113.76 21 334 134.16 −0.07 [−0.86, 0.72]
Ferrari, 1990 23 69.34 40.57 43 25.97 18.63 1.52 [0.96, 2.09]
RE Model (Q = 19.69, df = 2, p < 0.001; I² = 88.5%, τ² = 1.02) 0.36 [−0.86, 1.57]
−3.5
−1.75
0
1.75
3.5
Standardized Mean Difference

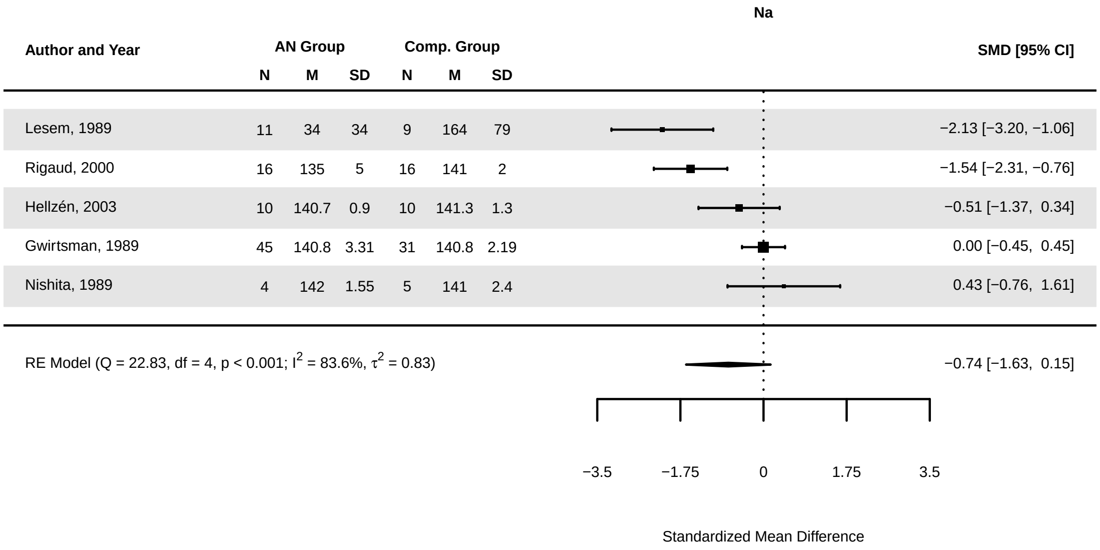
Na
Author and Year
AN Group
Comp. Group
SMD [95% CI]
N M SD N M SD
Lesem, 1989 11 34 34 9 164 79 −2.13 [−3.20, −1.06]
Rigaud, 2000 16 135 5 16 141 2 −1.54 [−2.31, −0.76]
Hellzén, 2003 10 140.7 0.9 10 141.3 1.3 −0.51 [−1.37, 0.34]
Gwirtsman, 1989 45 140.8 3.31 31 140.8 2.19 0.00 [−0.45, 0.45]
Nishita, 1989 4 142 1.55 5 141 2.4 0.43 [−0.76, 1.61]
RE Model (Q = 22.83, df = 4, p < 0.001; I² = 83.6%, τ² = 0.83) −0.74 [−1.63, 0.15]
−3.5
−1.75
0
1.75
3.5
Standardized Mean Difference

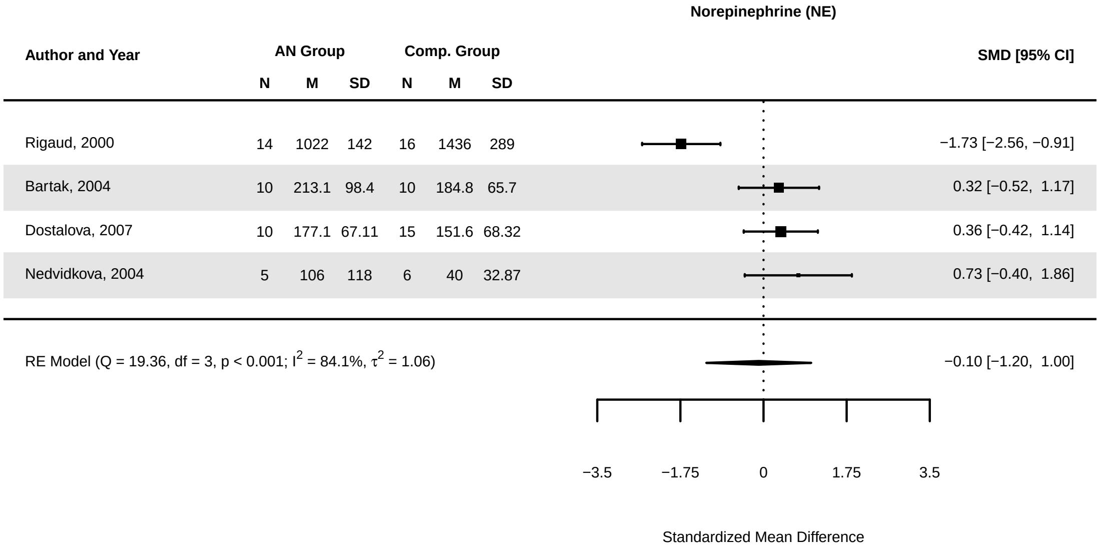
Norepinephrine (NE)
Author and Year
AN Group
Comp. Group
SMD [95% CI]
N M SD N M SD
Rigaud, 2000 14 1022 142 16 1436 289 −1.73 [−2.56, −0.91]
Bartak, 2004 10 213.1 98.4 10 184.8 65.7 0.32 [−0.52, 1.17]
Dostalova, 2007 10 177.1 67.11 15 151.6 68.32 0.36 [−0.42, 1.14]
Nedvidkova, 2004 5 106 118 6 40 32.87 0.73 [−0.40, 1.86]
RE Model (Q = 19.36, df = 3, p < 0.001; I² = 84.1%, τ² = 1.06) −0.10 [−1.20, 1.00]
−3.5
−1.75
0
1.75
3.5
Standardized Mean Difference

Obestatin

Author and Year	AN Group N	AN Group M	AN Group SD	Comp. Group N	Comp. Group M	Comp. Group SD	SMD [95% CI]
Monteleone, 2008	20	86.2	24.4	20	68.3	14.8	0.87 [0.23, 1.51]
Uehara, 2011	9	72.56	19.68	9	48.44	31.73	0.87 [−0.05, 1.79]
Germain, 2009	15	393	93.54	19	274.74	75.18	1.38 [0.64, 2.12]
Harada, 2008	10	64.5	6.72	10	49.2	6.45	2.22 [1.14, 3.31]
Nakahara, 2008	11	78.3	8.85	21	53.99	9.75	2.51 [1.57, 3.44]
RE Model (Q = 11.46, df = 4, p = 0.022; I^2 = 66.2%, τ^2 = 0.36)							1.51 [0.86, 2.16]

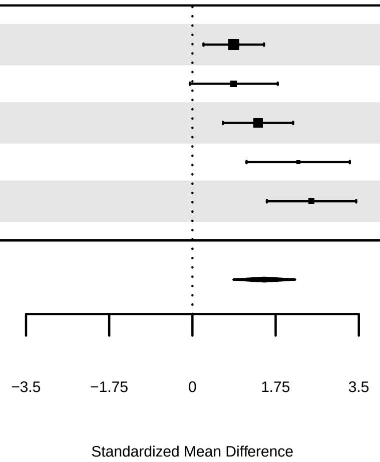

Osteocalcin

Author and Year	AN Group N	AN Group M	AN Group SD	Comp. Group N	Comp. Group M	Comp. Group SD	SMD [95% CI]
Urano, 2015	54	7.33	3.29	15	13.8	3.75	−1.89 [−2.53, −1.24]
Viapiana, 2007	55	11.94	8.98	15	23.15	7.69	−1.27 [−1.87, −0.67]
Estour, 2017	40	10.9	4.8	110	15.52	6.63	−0.74 [−1.11, −0.37]
Dominguez, 2007	28	8	3.05	11	8	2.7	0.00 [−0.68, 0.68]
Jacoangeli, 2002	49	6.2	0.29	24	6.1	0.15	0.39 [−0.10, 0.88]
RE Model (Q = 39.27, df = 4, p < 0.001; I^2 = 91.1%, τ^2 = 0.75)							−0.69 [−1.50, 0.11]

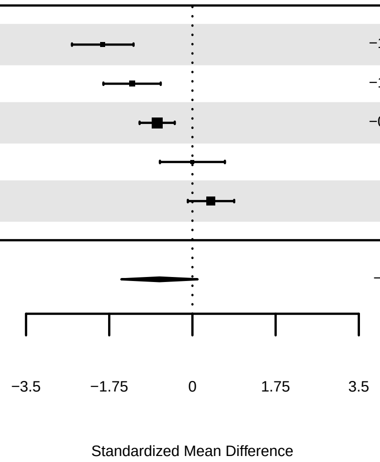

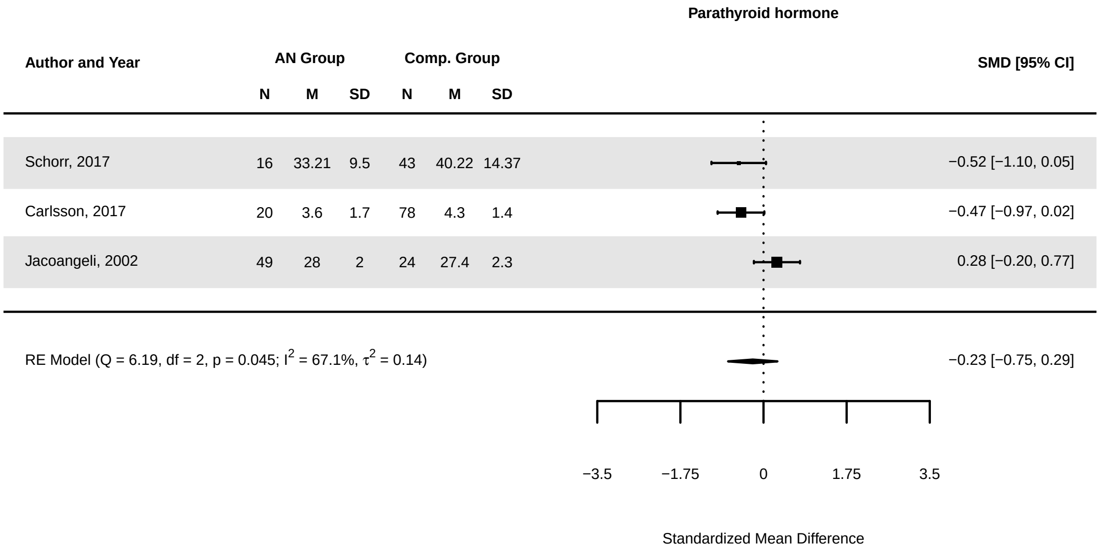
Parathyroid hormone
Author and Year
AN Group
Comp. Group
N M SD N M SD
SMD [95% CI]
Schorr, 2017 16 33.21 9.5 43 40.22 14.37 −0.52 [−1.10, 0.05]
Carlsson, 2017 20 3.6 1.7 78 4.3 1.4 −0.47 [−0.97, 0.02]
Jacoangeli, 2002 49 28 2 24 27.4 2.3 0.28 [−0.20, 0.77]
RE Model (Q = 6.19, df = 2, p = 0.045; I2 = 67.1%, τ2 = 0.14) −0.23 [−0.75, 0.29]
−3.5 −1.75 0 1.75 3.5
Standardized Mean Difference

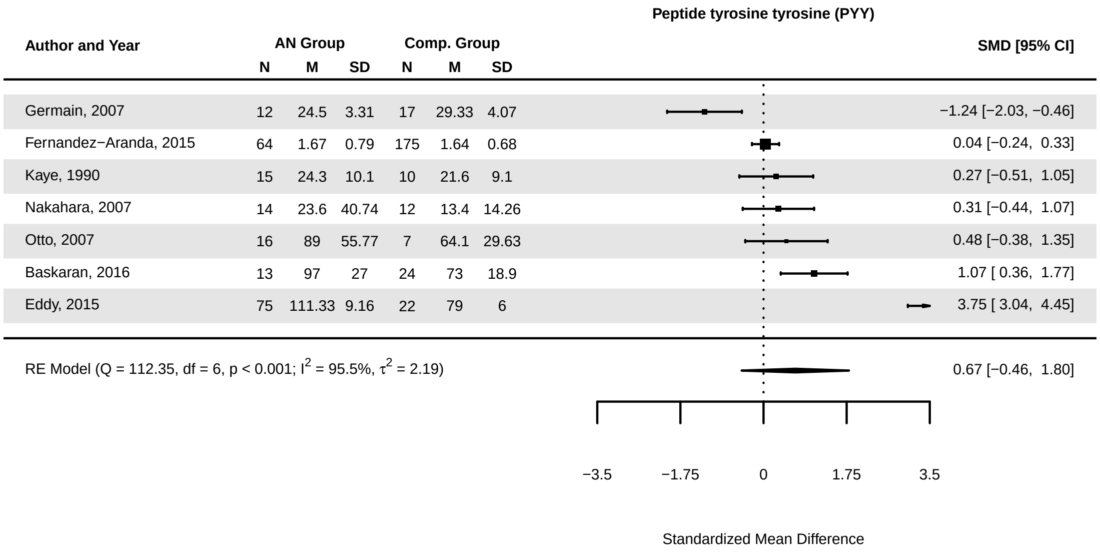
Peptide tyrosine tyrosine (PYY)
Author and Year
AN Group
Comp. Group
N M SD N M SD
SMD [95% CI]
Germain, 2007 12 24.5 3.31 17 29.33 4.07 −1.24 [−2.03, −0.46]
Fernandez−Aranda, 2015 64 1.67 0.79 175 1.64 0.68 0.04 [−0.24, 0.33]
Kaye, 1990 15 24.3 10.1 10 21.6 9.1 0.27 [−0.51, 1.05]
Nakahara, 2007 14 23.6 40.74 12 13.4 14.26 0.31 [−0.44, 1.07]
Otto, 2007 16 89 55.77 7 64.1 29.63 0.48 [−0.38, 1.35]
Baskaran, 2016 13 97 27 24 73 18.9 1.07 [0.36, 1.77]
Eddy, 2015 75 111.33 9.16 22 79 6 3.75 [3.04, 4.45]
RE Model (Q = 112.35, df = 6, p < 0.001; I2 = 95.5%, τ2 = 2.19) 0.67 [−0.46, 1.80]
−3.5 −1.75 0 1.75 3.5
Standardized Mean Difference

Potassium (K)

Author and Year	AN Group N	AN Group M	AN Group SD	Comp. Group N	Comp. Group M	Comp. Group SD	SMD [95% CI]
Rigaud, 2000	16	3.6	0.4	16	4.1	0.2	−1.54 [−2.32, −0.77]
Casper, 1988	7	3.8	0.53	14	4.08	0.25	−0.74 [−1.64, 0.16]
Gwirtsman, 1989	45	4.14	0.39	31	4.28	0.32	−0.38 [−0.84, 0.08]
Hellzén, 2003	10	4.2	0.5	10	4.2	0.2	0.00 [−0.84, 0.84]
Tamia, 1991	19	1.66	0.59	8	1.59	0.21	0.13 [−0.67, 0.93]
RE Model (Q = 11.22, df = 4, p = 0.024; I^2 = 67.1%, τ^2 = 0.28)							−0.50 [−1.07, 0.07]

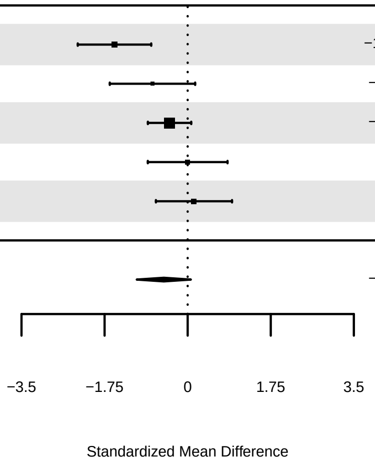

Prolactin

Author and Year	AN Group N	AN Group M	AN Group SD	Comp. Group N	Comp. Group M	Comp. Group SD	SMD [95% CI]
Arimura, 2010	20	3.87	3.28	12	13.9	4.1	−2.71 [−3.68, −1.75]
Hadigan, 1995	10	6.64	2.4	8	14.4	4	−2.31 [−3.47, −1.15]
Monteleone, 2002	33	7.2	3.2	38	18	7.4	−1.83 [−2.38, −1.28]
Mori, 1990	9	10	3.95	6	13.5	5.14	−0.74 [−1.75, 0.27]
Broglio, 2004	9	140	124.45	7	208	78.38	−0.60 [−1.56, 0.36]
Brambilla, 2003	22	2.6	2.2	20	3.6	1.3	−0.54 [−1.14, 0.07]
Ferrari, 1990	23	10.35	6.61	43	13.3	6.33	−0.45 [−0.96, 0.05]
Estour, 2017	40	288	253	110	359.2	240.07	−0.29 [−0.65, 0.07]
Mondelli, 2006	6	6.65	2.23	6	7.1	4.47	−0.12 [−1.16, 0.93]
Miljic, 2006	9	538.8	754.05	10	353.6	226.5	0.33 [−0.54, 1.19]
RE Model (Q = 52.96, df = 9, p < 0.001; I^2 = 86.2%, τ^2 = 0.75)							−0.90 [−1.49, −0.30]

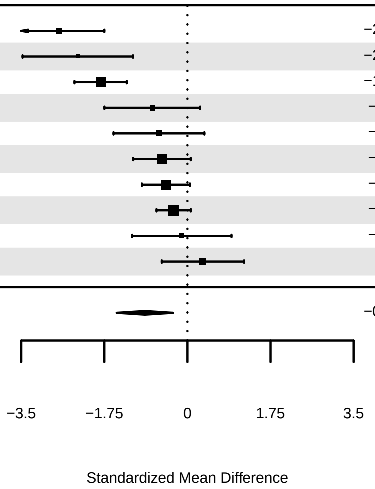

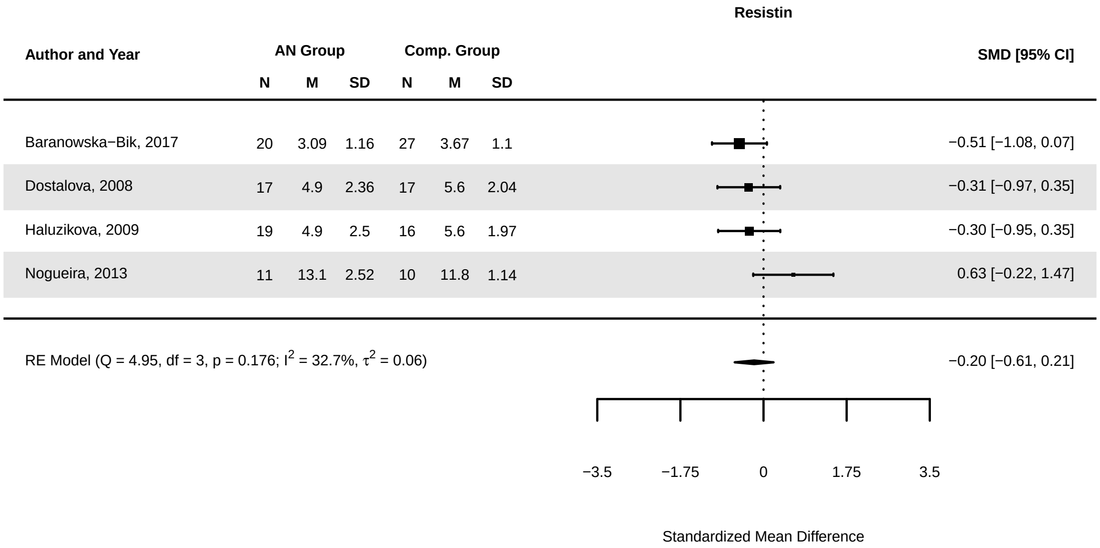
Resistin
Author and Year
AN Group
Comp. Group
N M SD N M SD
SMD [95% CI]
Baranowska−Bik, 2017 20 3.09 1.16 27 3.67 1.1 −0.51 [−1.08, 0.07]
Dostalova, 2008 17 4.9 2.36 17 5.6 2.04 −0.31 [−0.97, 0.35]
Haluzikova, 2009 19 4.9 2.5 16 5.6 1.97 −0.30 [−0.95, 0.35]
Nogueira, 2013 11 13.1 2.52 10 11.8 1.14 0.63 [−0.22, 1.47]
RE Model (Q = 4.95, df = 3, p = 0.176; I2 = 32.7%, τ2 = 0.06) −0.20 [−0.61, 0.21]
−3.5
−1.75
0
1.75
3.5
Standardized Mean Difference

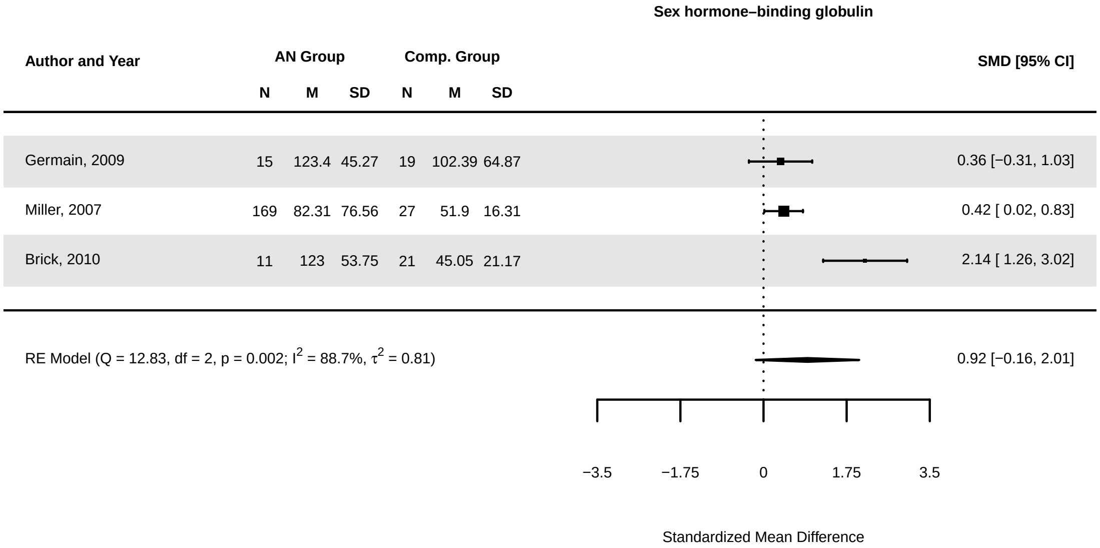
Sex hormone–binding globulin
Author and Year
AN Group
Comp. Group
N M SD N M SD
SMD [95% CI]
Germain, 2009 15 123.4 45.27 19 102.39 64.87 0.36 [−0.31, 1.03]
Miller, 2007 169 82.31 76.56 27 51.9 16.31 0.42 [0.02, 0.83]
Brick, 2010 11 123 53.75 21 45.05 21.17 2.14 [1.26, 3.02]
RE Model (Q = 12.83, df = 2, p = 0.002; I2 = 88.7%, τ2 = 0.81) 0.92 [−0.16, 2.01]
−3.5
−1.75
0
1.75
3.5
Standardized Mean Difference

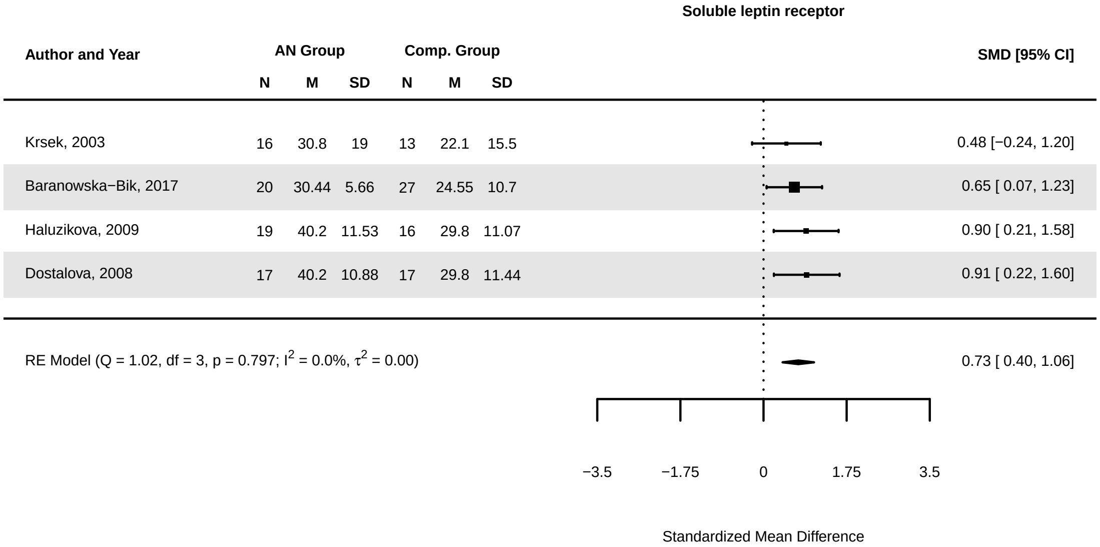
Soluble leptin receptor
Author and Year
AN Group
Comp. Group
SMD [95% CI]
N M SD N M SD
Krsek, 2003 16 30.8 19 13 22.1 15.5 0.48 [−0.24, 1.20]
Baranowska−Bik, 2017 20 30.44 5.66 27 24.55 10.7 0.65 [0.07, 1.23]
Haluzikova, 2009 19 40.2 11.53 16 29.8 11.07 0.90 [0.21, 1.58]
Dostalova, 2008 17 40.2 10.88 17 29.8 11.44 0.91 [0.22, 1.60]
RE Model (Q = 1.02, df = 3, p = 0.797; I2 = 0.0%, τ2 = 0.00) 0.73 [0.40, 1.06]
−3.5
−1.75
0
1.75
3.5
Standardized Mean Difference

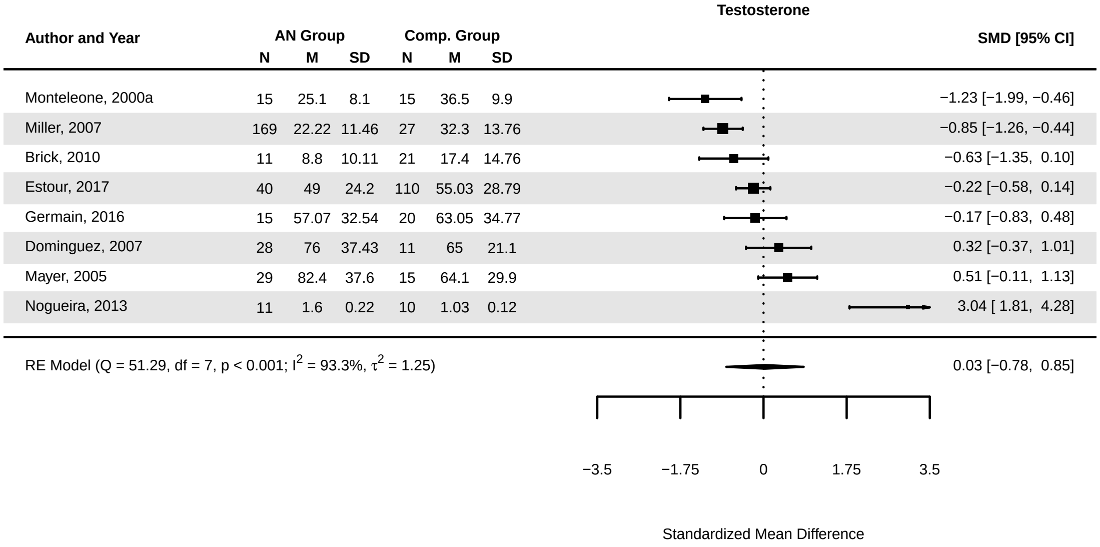
Testosterone
Author and Year
AN Group
Comp. Group
SMD [95% CI]
N M SD N M SD
Monteleone, 2000a 15 25.1 8.1 15 36.5 9.9 −1.23 [−1.99, −0.46]
Miller, 2007 169 22.22 11.46 27 32.3 13.76 −0.85 [−1.26, −0.44]
Brick, 2010 11 8.8 10.11 21 17.4 14.76 −0.63 [−1.35, 0.10]
Estour, 2017 40 49 24.2 110 55.03 28.79 −0.22 [−0.58, 0.14]
Germain, 2016 15 57.07 32.54 20 63.05 34.77 −0.17 [−0.83, 0.48]
Dominguez, 2007 28 76 37.43 11 65 21.1 0.32 [−0.37, 1.01]
Mayer, 2005 29 82.4 37.6 15 64.1 29.9 0.51 [−0.11, 1.13]
Nogueira, 2013 11 1.6 0.22 10 1.03 0.12 3.04 [1.81, 4.28]
RE Model (Q = 51.29, df = 7, p < 0.001; I2 = 93.3%, τ2 = 1.25) 0.03 [−0.78, 0.85]
−3.5
−1.75
0
1.75
3.5
Standardized Mean Difference

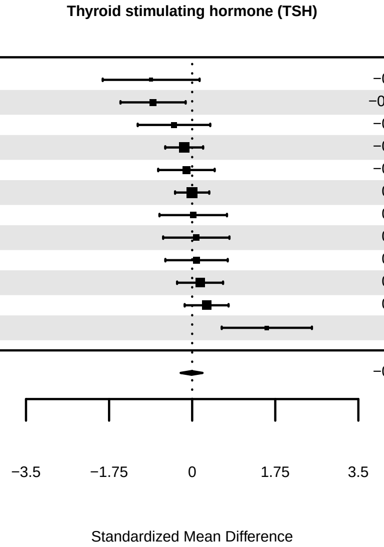

Author and Year	AN Group N	AN Group M	AN Group SD	Comp. Group N	Comp. Group M	Comp. Group SD	SMD [95% CI]
Mori, 1990	9	1.41	0.36	6	1.9	0.73	−0.86 [−1.88, 0.16]
Kaválková, 2012	18	1.45	0.86	16	2.25	1.04	−0.82 [−1.51, −0.14]
Stengel, 2013	8	1.9	1.32	32	2.55	1.73	−0.38 [−1.15, 0.38]
Barja−Fernández, 2015	30	1.6	0.7	119	3.07	9.69	−0.17 [−0.57, 0.23]
Brambilla, 2003	22	1.6	0.9	20	1.7	0.7	−0.12 [−0.72, 0.47]
Estour, 2017	40	1.8	2.1	110	1.8	0.8	0.00 [−0.36, 0.36]
Brick, 2010	11	1.7	1.26	21	1.67	1.35	0.02 [−0.69, 0.73]
Arimura, 2010	20	1.92	2.84	12	1.71	1.1	0.09 [−0.61, 0.78]
Dostalova, 2008	17	1.8	1.04	17	1.7	1.08	0.09 [−0.56, 0.75]
Jacoangeli, 2002	49	1.93	1.03	24	1.73	1.47	0.17 [−0.32, 0.65]
Onur, 2005	28	1.7	1.1	49	1.4	0.9	0.30 [−0.16, 0.77]
Nogueira, 2013	11	2	0.31	10	1.5	0.3	1.57 [0.62, 2.52]
RE Model (Q = 22.87, df = 11, p = 0.018; I^2 = 44.9%, τ^2 = 0.07)							−0.01 [−0.24, 0.22]

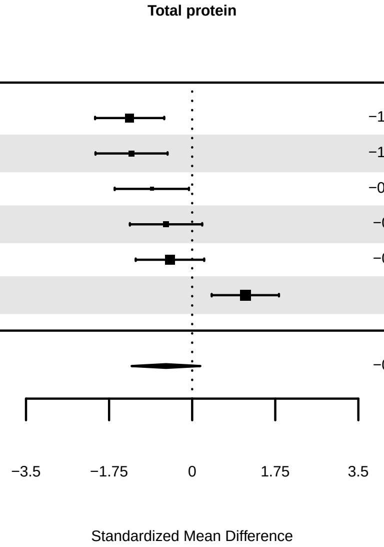

Author and Year	AN Group N	AN Group M	AN Group SD	Comp. Group N	Comp. Group M	Comp. Group SD	SMD [95% CI]
Kaválková, 2012	18	69.66	7.58	16	78.4	4.95	−1.32 [−2.04, −0.59]
Flierl, 2011	14	61.9	8.2	17	73.7	9.6	−1.28 [−2.04, −0.52]
Stengel, 2013	8	7	0.52	32	7.43	0.49	−0.85 [−1.63, −0.07]
Nakahara, 2007	14	6.2	2.52	12	7.5	1.98	−0.55 [−1.31, 0.21]
Krsek, 2003	16	73.8	4.2	13	75.7	3.6	−0.47 [−1.19, 0.25]
Haluzi´K, 1999	17	82.4	3.13	17	76.9	6.02	1.12 [0.41, 1.83]
RE Model (Q = 29.71, df = 5, p < 0.001; I^2 = 82.5%, τ^2 = 0.67)							−0.55 [−1.28, 0.17]

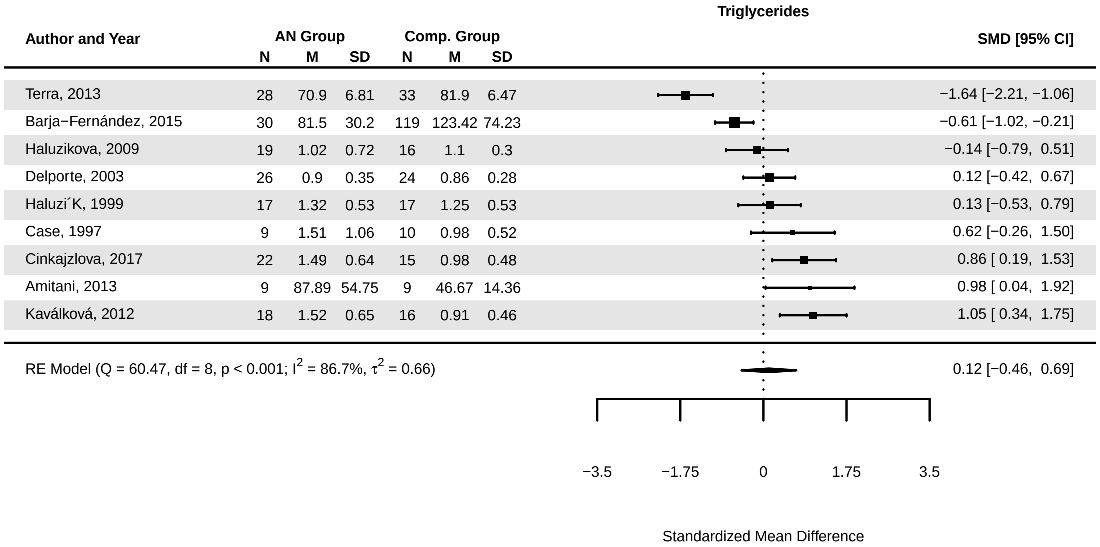
Triglycerides
Author and Year
AN Group
Comp. Group
N M SD N M SD
SMD [95% CI]
Terra, 2013 28 70.9 6.81 33 81.9 6.47 −1.64 [−2.21, −1.06]
Barja−Fernández, 2015 30 81.5 30.2 119 123.42 74.23 −0.61 [−1.02, −0.21]
Haluzikova, 2009 19 1.02 0.72 16 1.1 0.3 −0.14 [−0.79, 0.51]
Delporte, 2003 26 0.9 0.35 24 0.86 0.28 0.12 [−0.42, 0.67]
Haluzi´K, 1999 17 1.32 0.53 17 1.25 0.53 0.13 [−0.53, 0.79]
Case, 1997 9 1.51 1.06 10 0.98 0.52 0.62 [−0.26, 1.50]
Cinkajzlova, 2017 22 1.49 0.64 15 0.98 0.48 0.86 [0.19, 1.53]
Amitani, 2013 9 87.89 54.75 9 46.67 14.36 0.98 [0.04, 1.92]
Kaválková, 2012 18 1.52 0.65 16 0.91 0.46 1.05 [0.34, 1.75]
RE Model (Q = 60.47, df = 8, p < 0.001; I2 = 86.7%, τ2 = 0.66) 0.12 [−0.46, 0.69]
−3.5 −1.75 0 1.75 3.5
Standardized Mean Difference

Supplemental Document 4. Individual Funnel Plots for all 52 Peripheral Biomarkers.

The funnel plot displays the observed effect sizes on the x-axis and the corresponding standard errors (i.e., the square root of the sampling variances) on the y-axis. The vertical line indicates the omnibus estimate based on the model. A pseudo confidence interval region is drawn around this value with bounds equal to ±1.96 SE, where SE is the standard error value from the y-axis.

If asymmetry is noted on one side of the plot or the other, this is an indication that publication bias might be present. Egger's regression test and three-parameter selection models (see main document for details) provides quantitative support for potential publication bias.

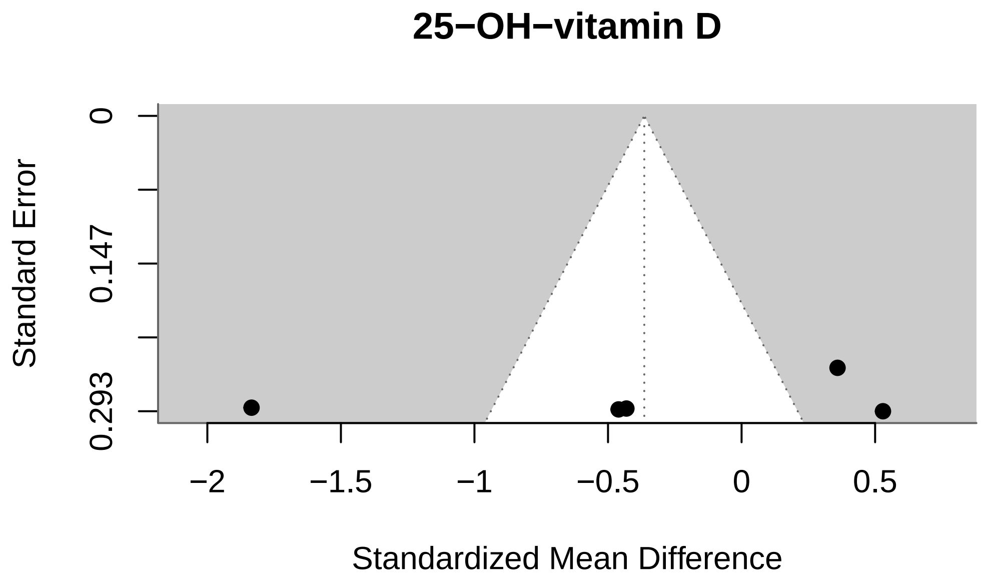

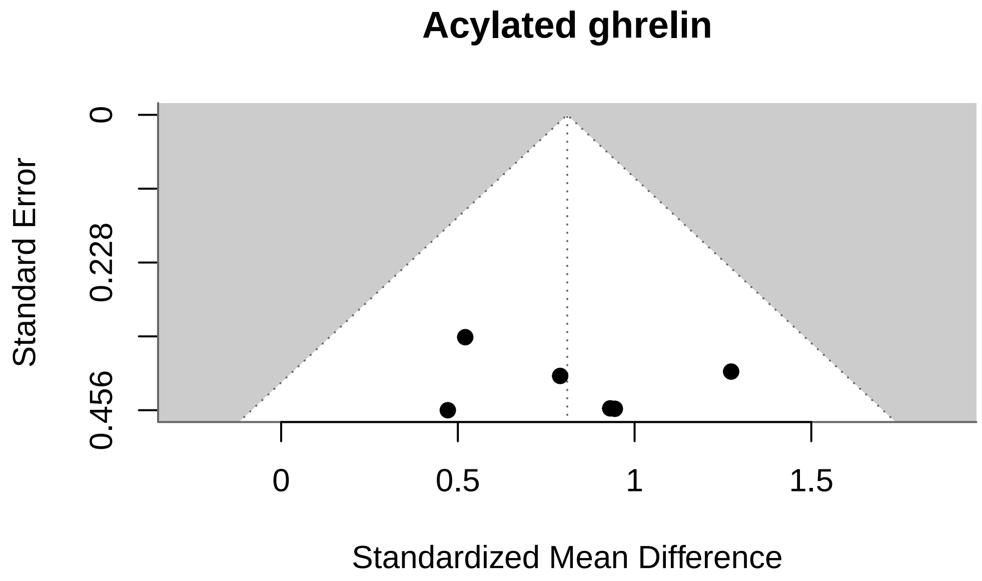
Acylated ghrelin
Standard Error
0
0.228
0.456
0
0.5
1
1.5
Standardized Mean Difference

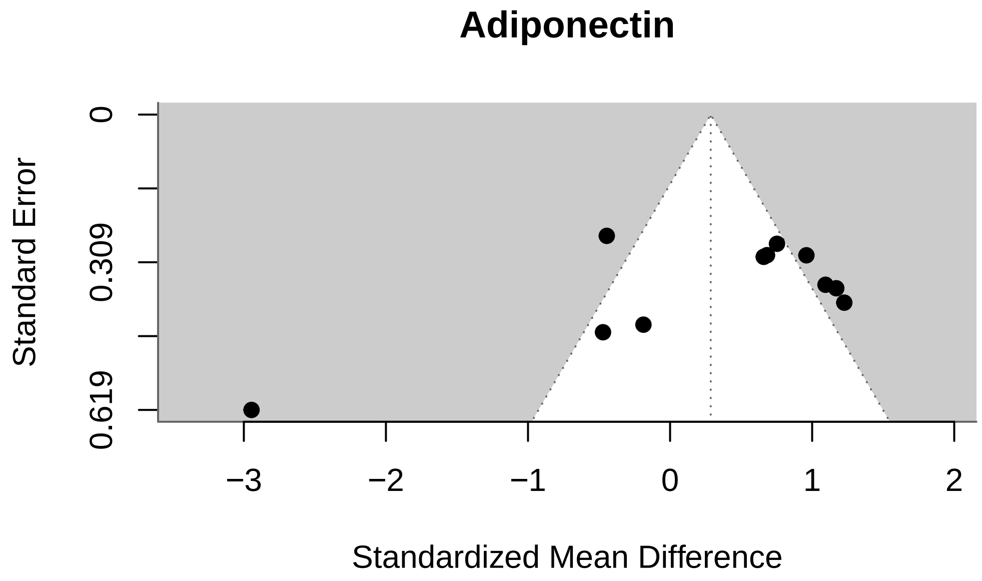
Adiponectin
Standard Error
0
0.309
0.619
−3
−2
−1
0
1
2
Standardized Mean Difference

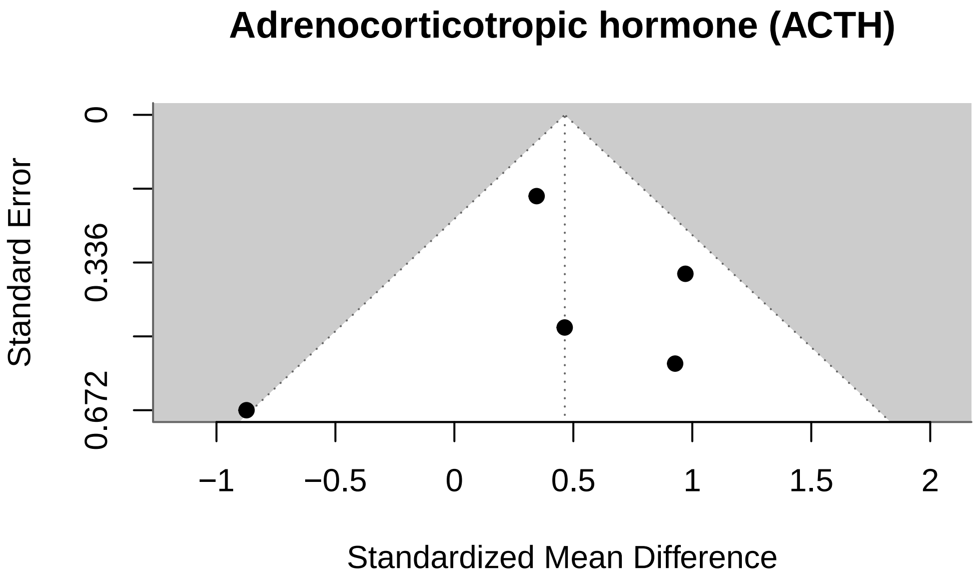
Adrenocorticotropic hormone (ACTH)
Standard Error
0
0.336
0.672
−1
−0.5
0
0.5
1
1.5
2
Standardized Mean Difference

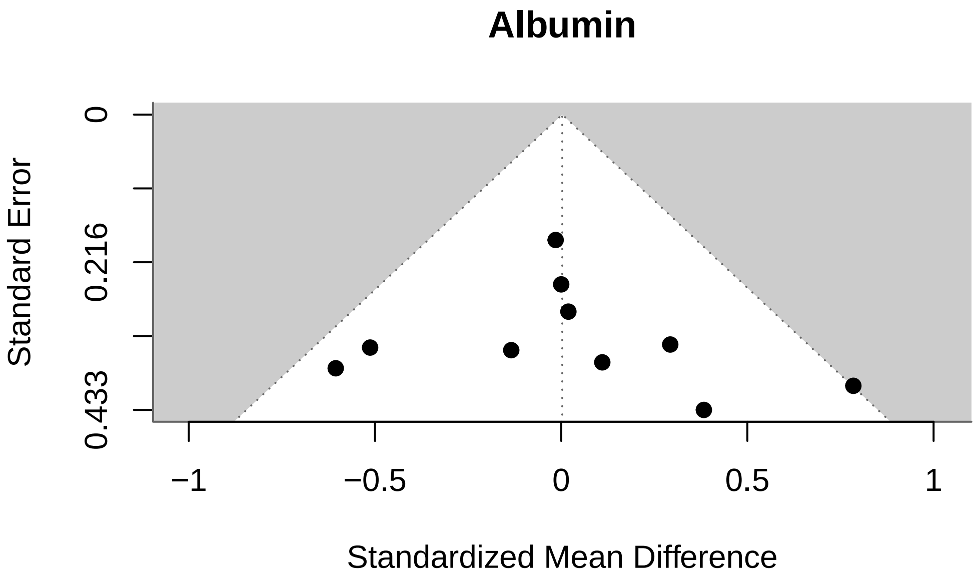
Albumin
Standard Error
0
0.216
0.433
−1
−0.5
0
0.5
1
Standardized Mean Difference

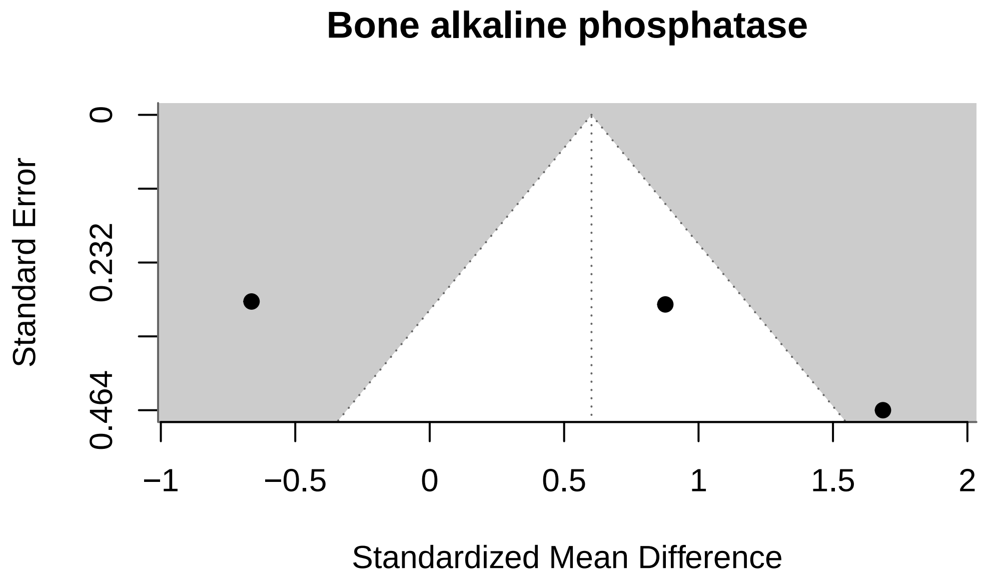
Bone alkaline phosphatase
Standard Error
0
0.232
0.464
−1
−0.5
0
0.5
1
1.5
2
Standardized Mean Difference

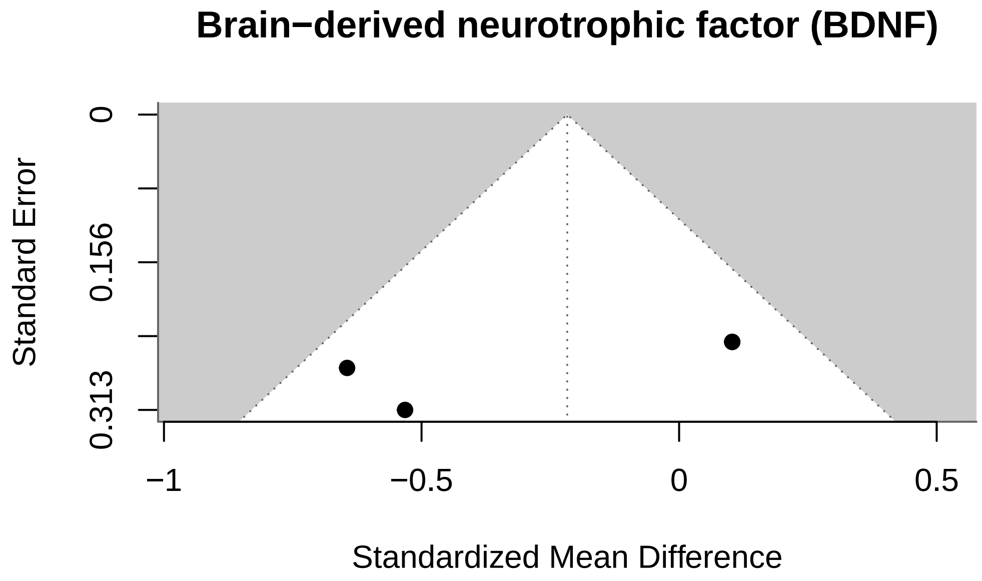
Brain−derived neurotrophic factor (BDNF)
Standard Error
0
0.156
0.313
−1
−0.5
0
0.5
Standardized Mean Difference

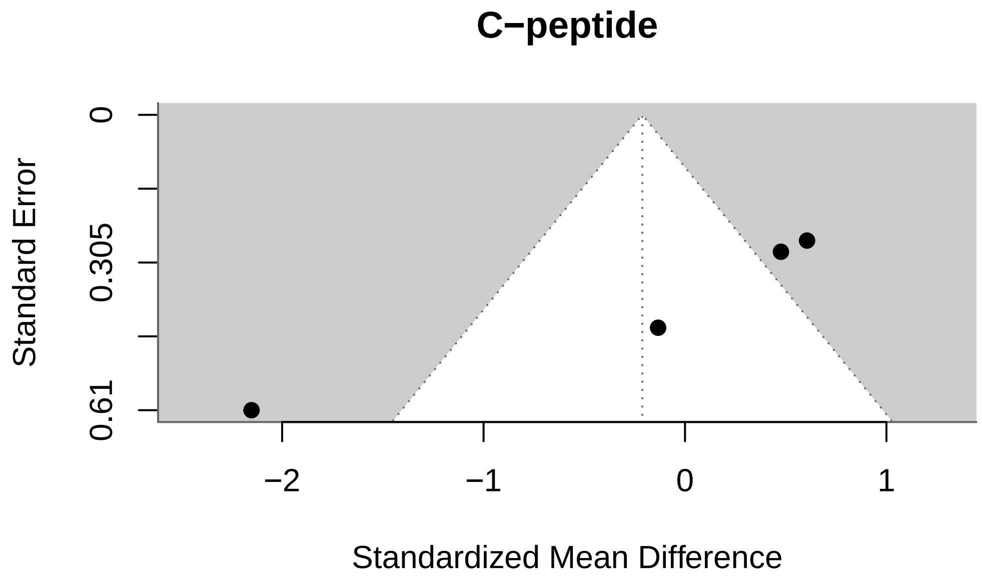
C−peptide
Standard Error
0
0.305
0.61
−2
−1
0
1
Standardized Mean Difference

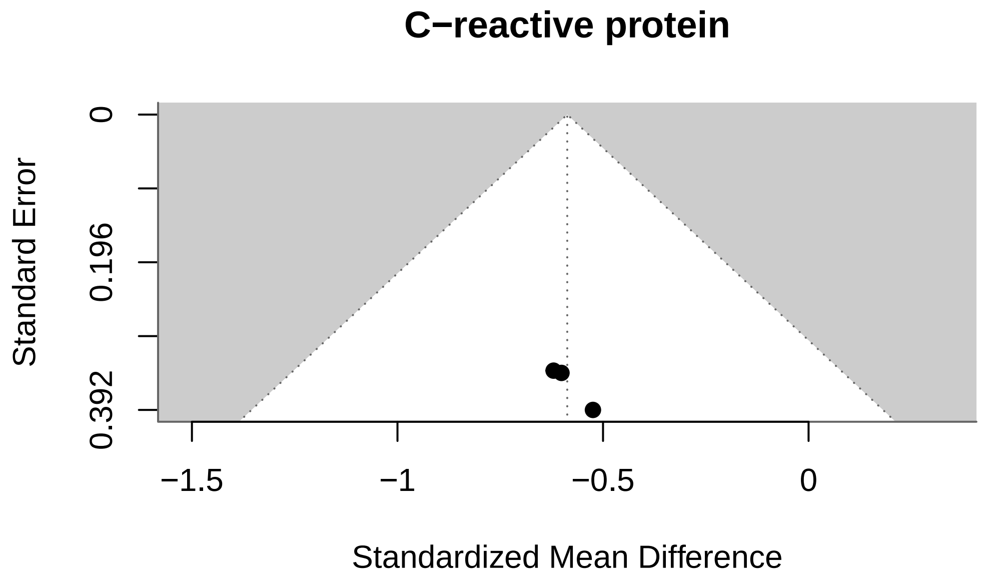
C−reactive protein
Standard Error
0
0.196
0.392
−1.5
−1
−0.5
0
Standardized Mean Difference

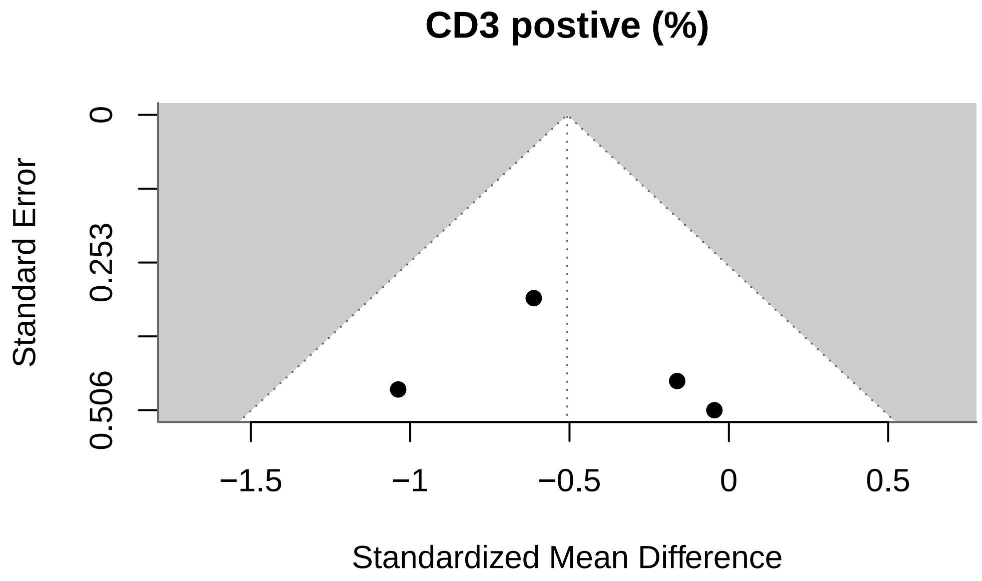
CD3 postive (%)
Standard Error
0
0.253
0.506
−1.5
−1
−0.5
0
0.5
Standardized Mean Difference

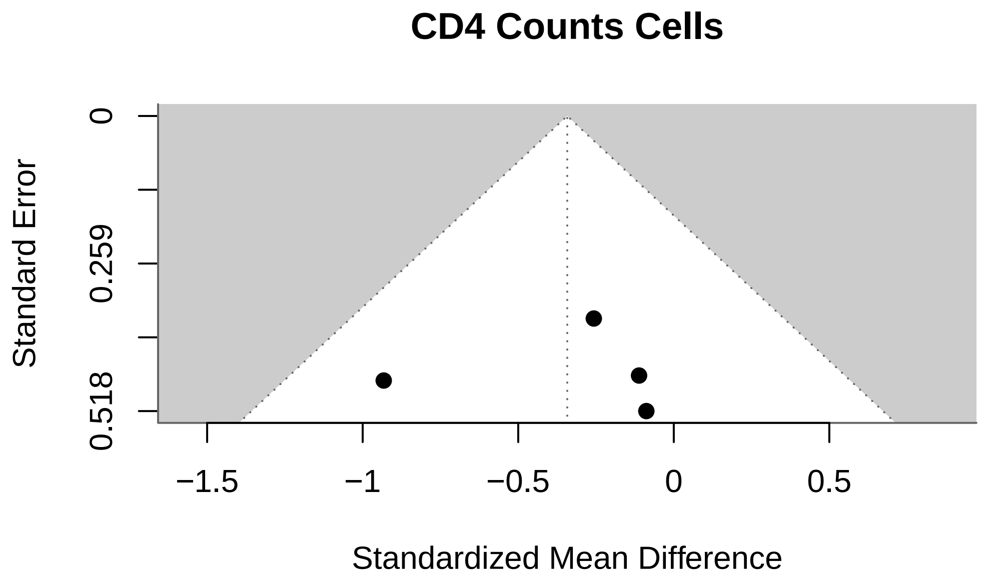
CD4 Counts Cells
Standard Error
0
0.259
0.518
−1.5
−1
−0.5
0
0.5
Standardized Mean Difference

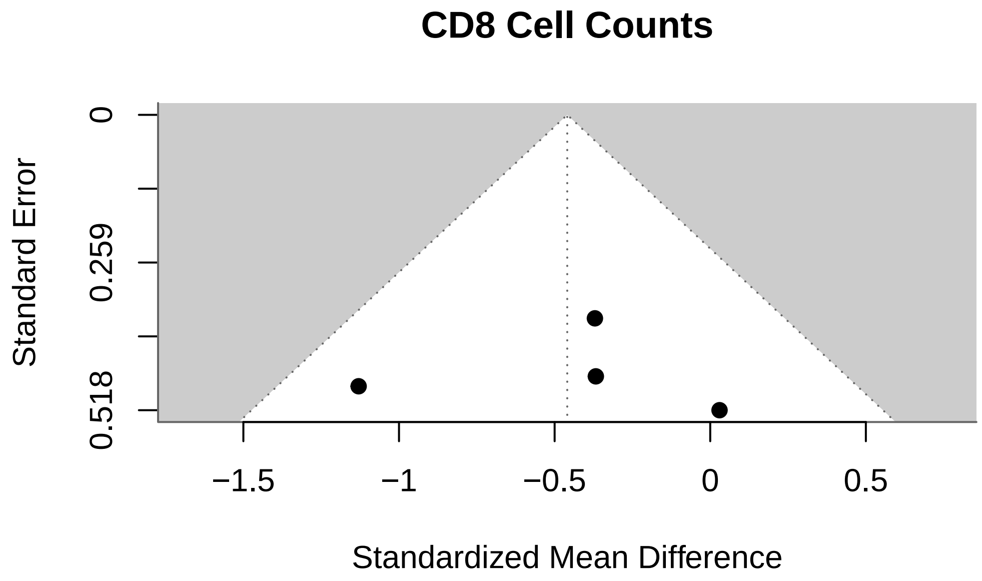
CD8 Cell Counts
Standard Error
0
0.259
0.518
−1.5
−1
−0.5
0
0.5
Standardized Mean Difference

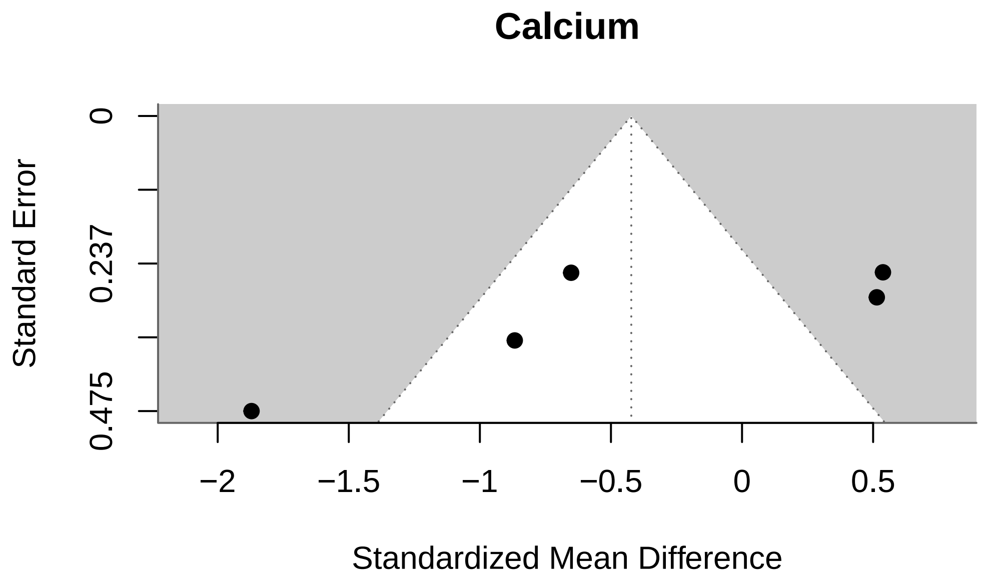
Calcium
Standard Error
0
0.237
0.475
−2
−1.5
−1
−0.5
0
0.5
Standardized Mean Difference

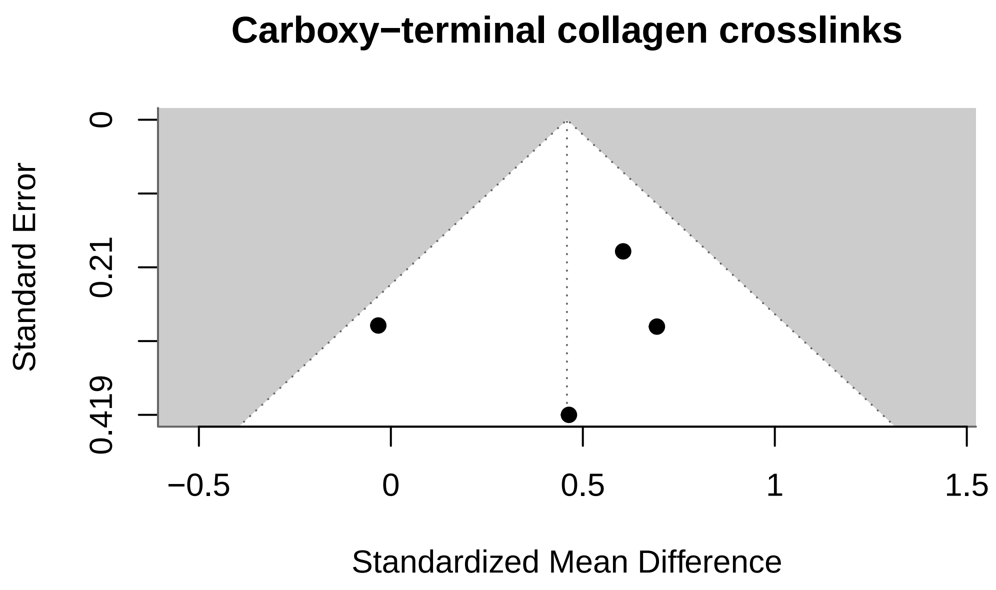

Carboxy−terminal collagen crosslinks
Standard Error
0
0.21
0.419
−0.5
0
0.5
1
1.5
Standardized Mean Difference

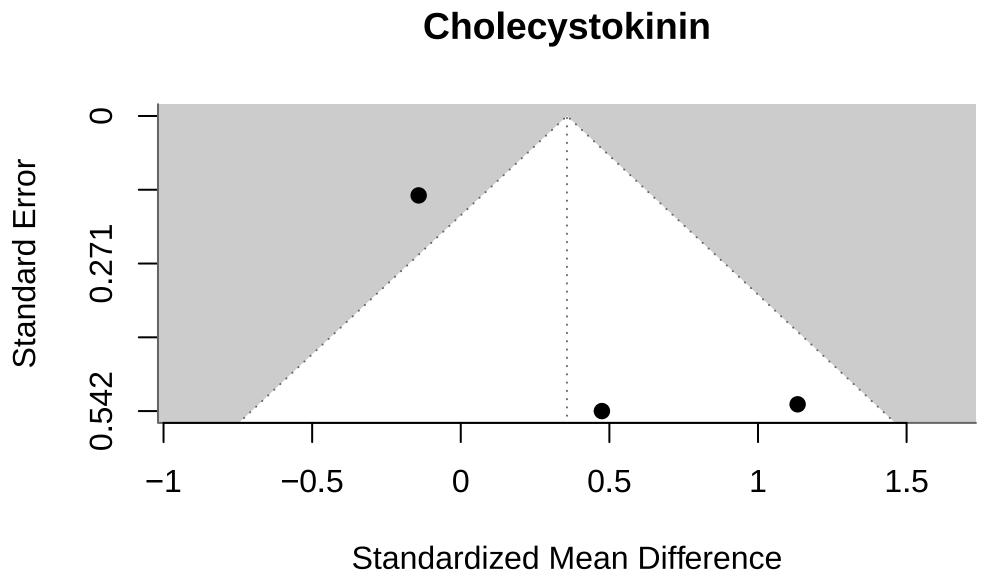

Cholecystokinin
Standard Error
0
0.271
0.542
−1
−0.5
0
0.5
1
1.5
Standardized Mean Difference

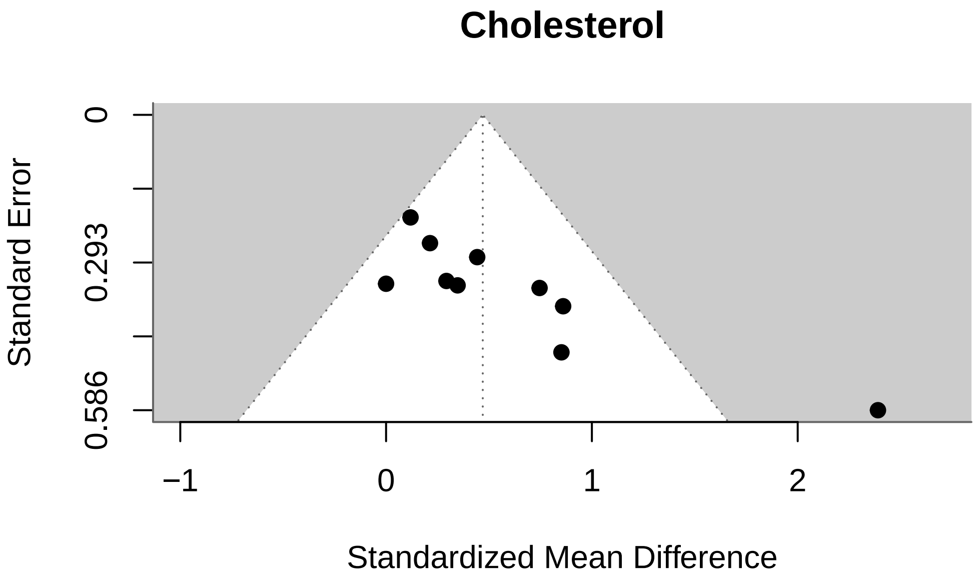
Cholesterol
Standard Error
0
0.293
0.586
−1
0
1
2
Standardized Mean Difference

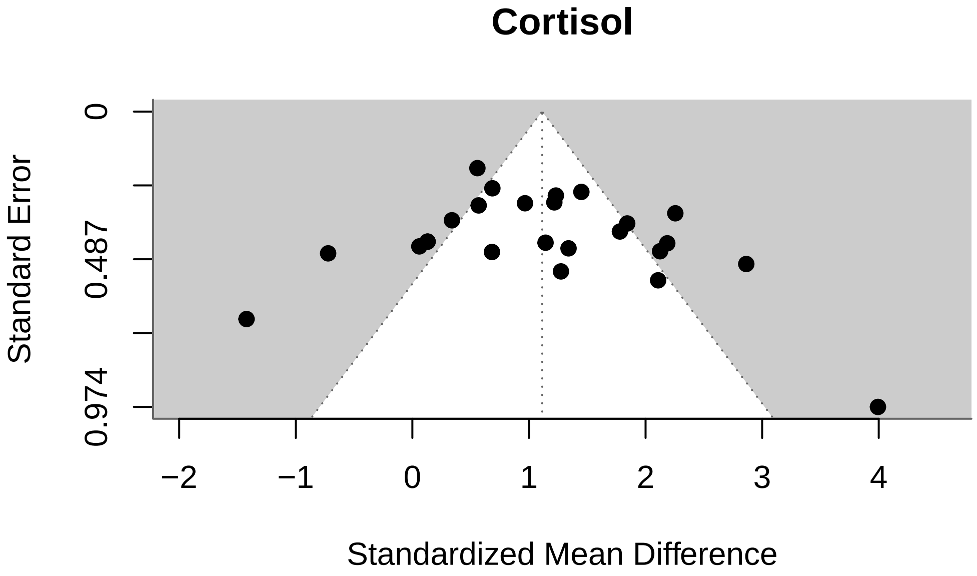
Cortisol
Standard Error
0
0.487
0.974
−2
−1
0
1
2
3
4
Standardized Mean Difference

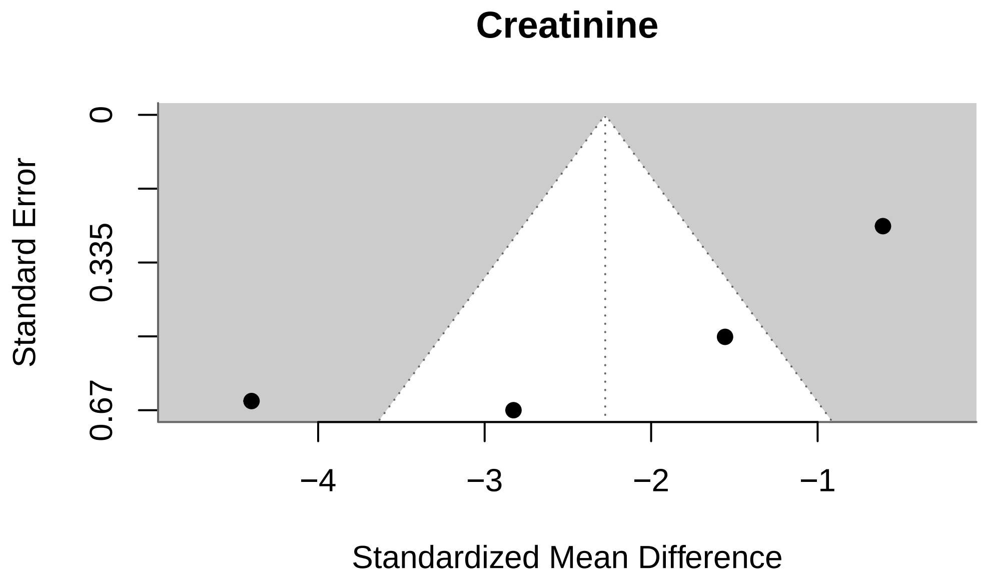
Creatinine
Standard Error
0
0.335
0.67
−4
−3
−2
−1
Standardized Mean Difference

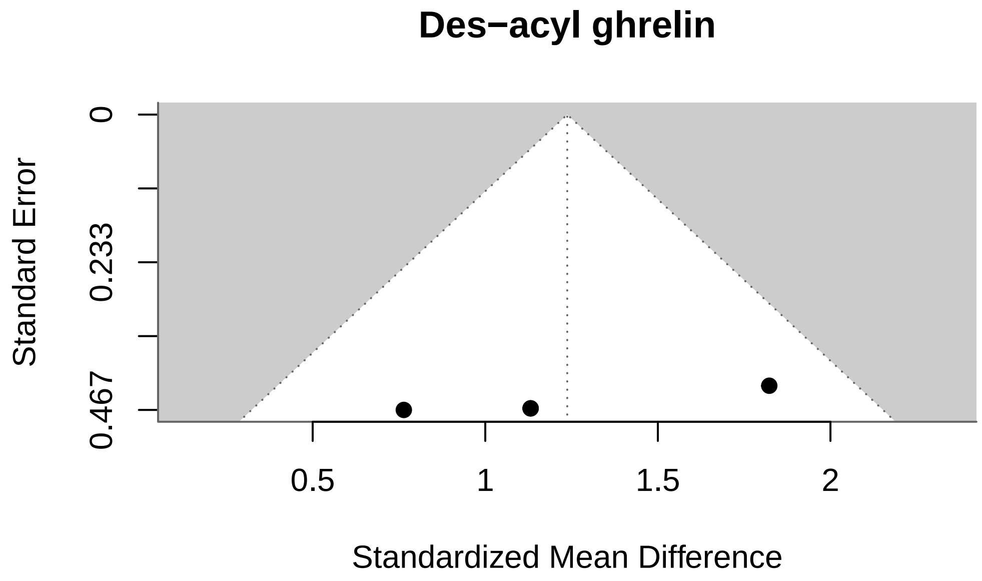
Des−acyl ghrelin
Standard Error
0
0.233
0.467
0.5
1
1.5
2
Standardized Mean Difference

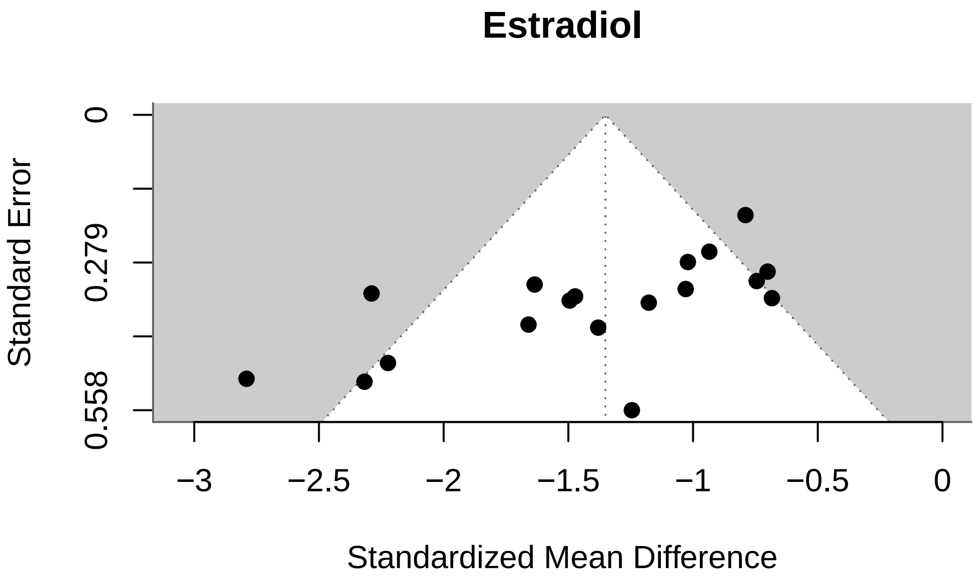
Estradiol
Standard Error
0
0.279
0.558
−3
−2.5
−2
−1.5
−1
−0.5
0
Standardized Mean Difference

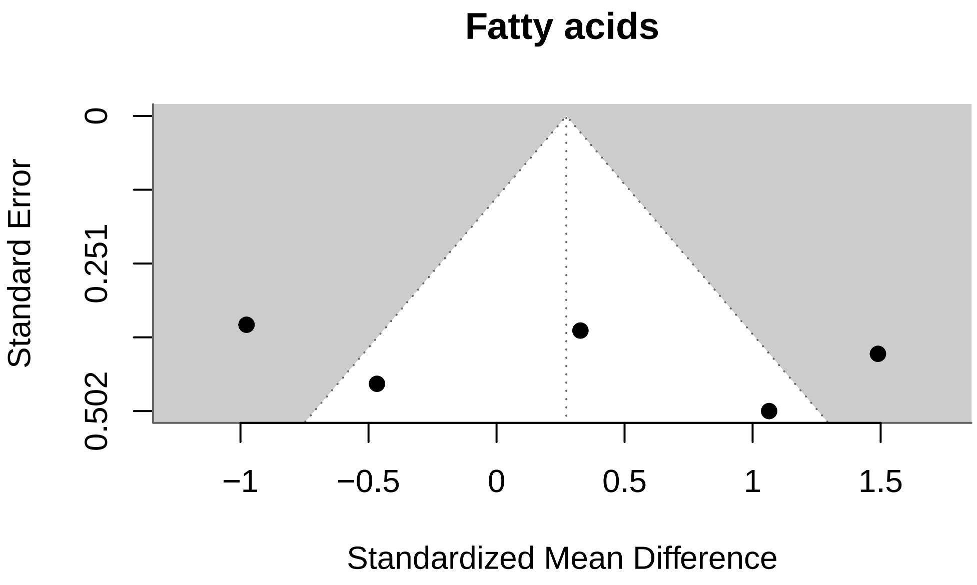
Fatty acids
Standard Error
0
0.251
0.502
−1
−0.5
0
0.5
1
1.5
Standardized Mean Difference

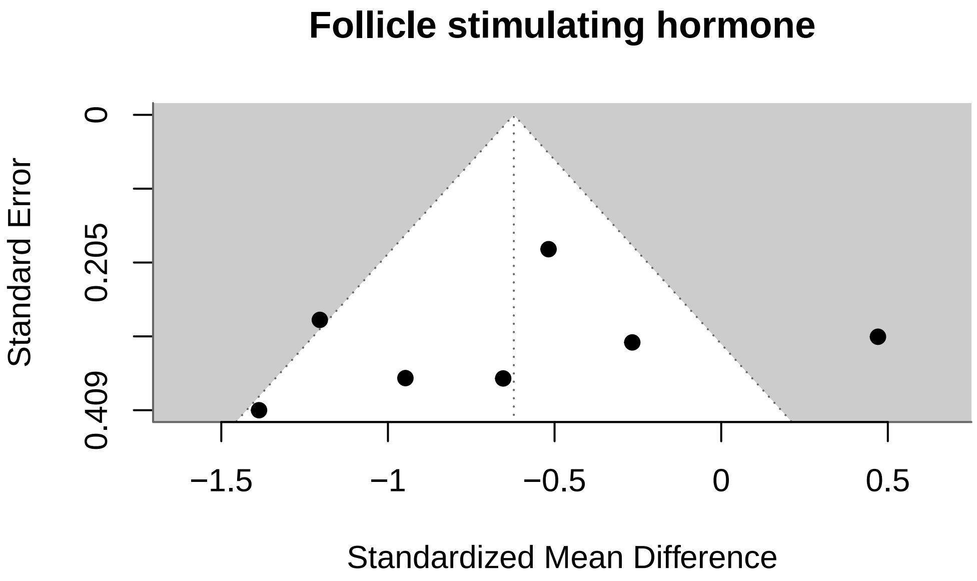
Follicle stimulating hormone
Standard Error
0
0.205
0.409
−1.5
−1
−0.5
0
0.5
Standardized Mean Difference

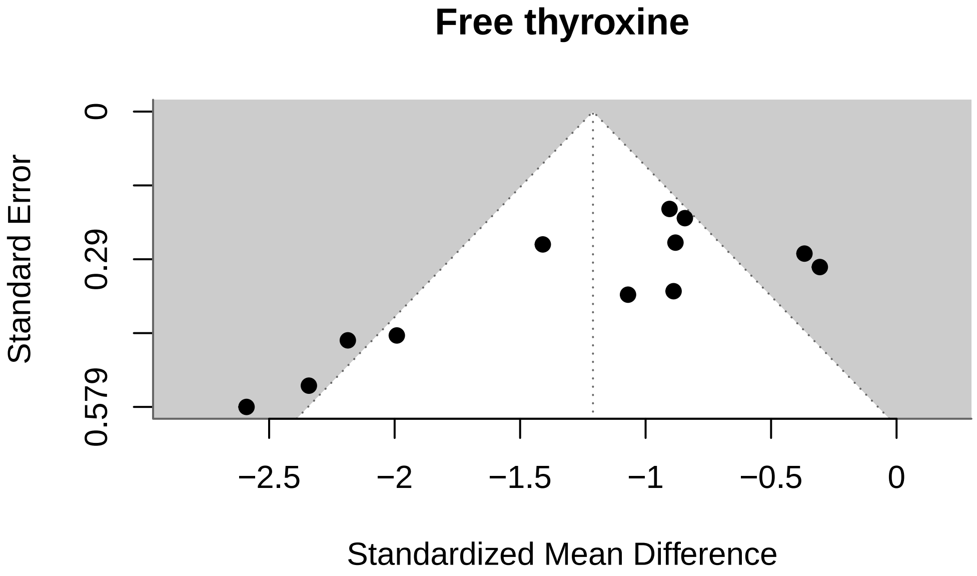
Free thyroxine
Standard Error
0
0.29
0.579
−2.5
−2
−1.5
−1
−0.5
0
Standardized Mean Difference

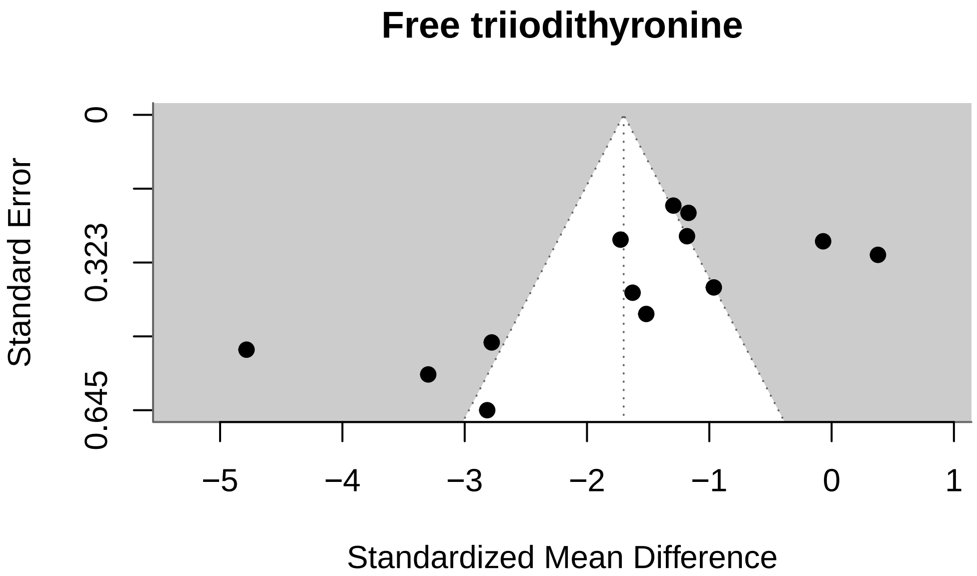
Free triiodithyronine
Standard Error
0
0.323
0.645
−5
−4
−3
−2
−1
0
1
Standardized Mean Difference

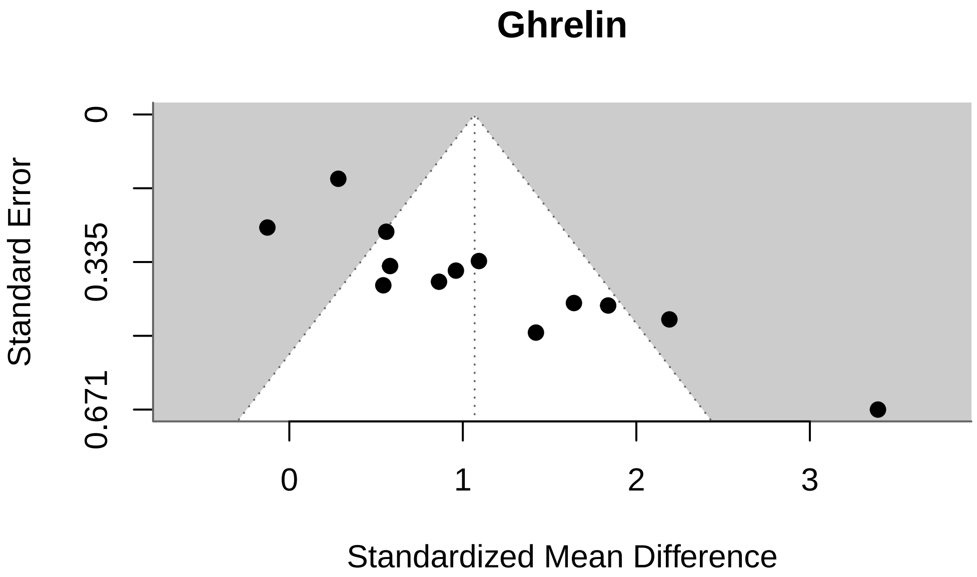
Ghrelin
Standard Error
0
0.335
0.671
0
1
2
3
Standardized Mean Difference

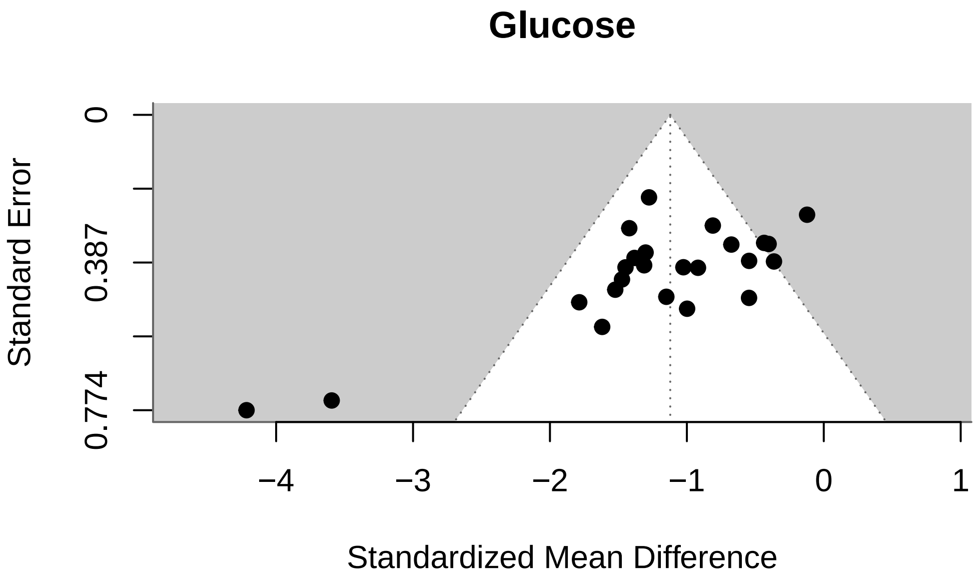
Glucose
Standard Error
0
0.387
0.774
−4
−3
−2
−1
0
1
Standardized Mean Difference

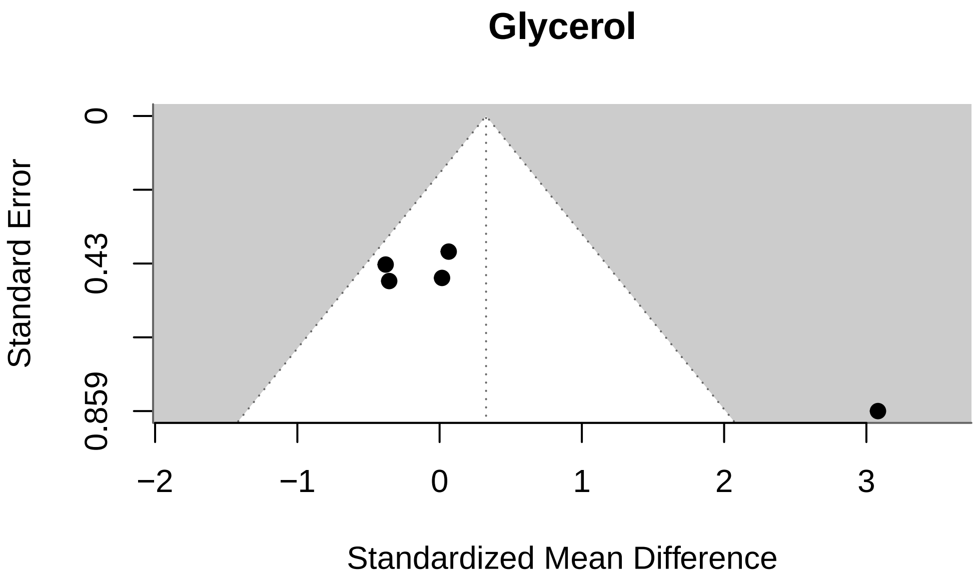
Glycerol
Standard Error
0
0.43
0.859
−2
−1
0
1
2
3
Standardized Mean Difference

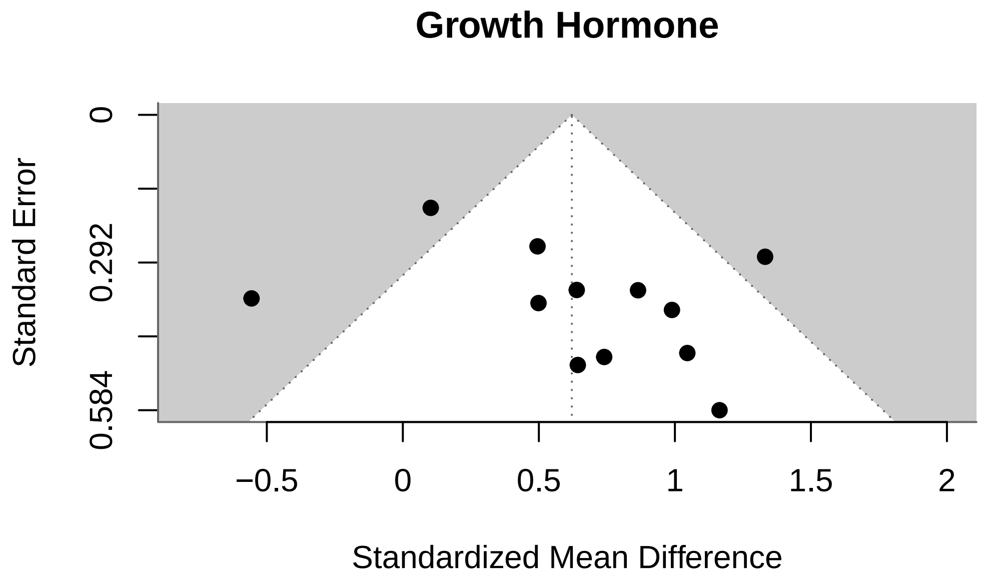
Growth Hormone
Standard Error
0
0.292
0.584
−0.5
0
0.5
1
1.5
2
Standardized Mean Difference

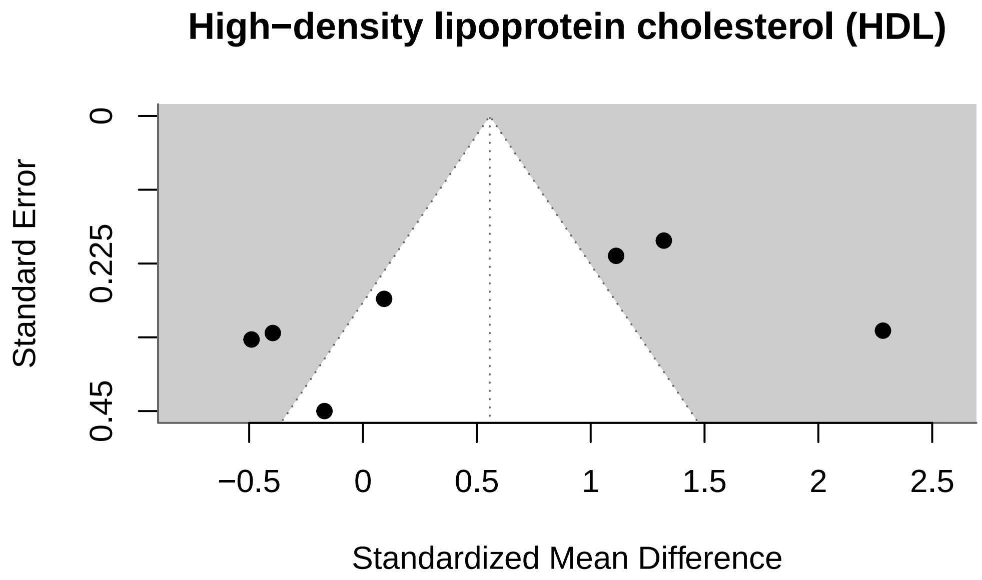
High−density lipoprotein cholesterol (HDL)
Standard Error
0
0.225
0.45
−0.5
0
0.5
1
1.5
2
2.5
Standardized Mean Difference

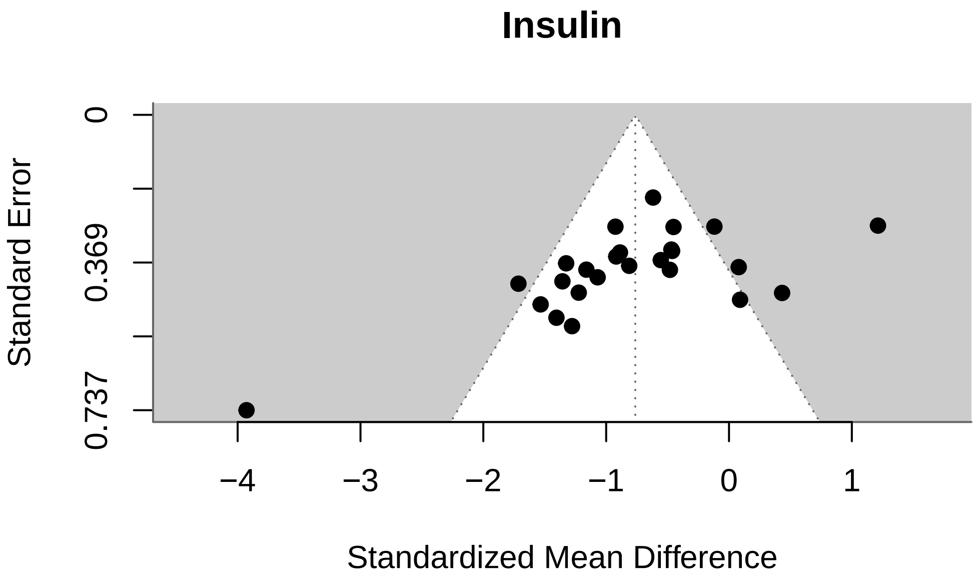
Insulin
Standard Error
0
0.369
0.737
−4
−3
−2
−1
0
1
Standardized Mean Difference

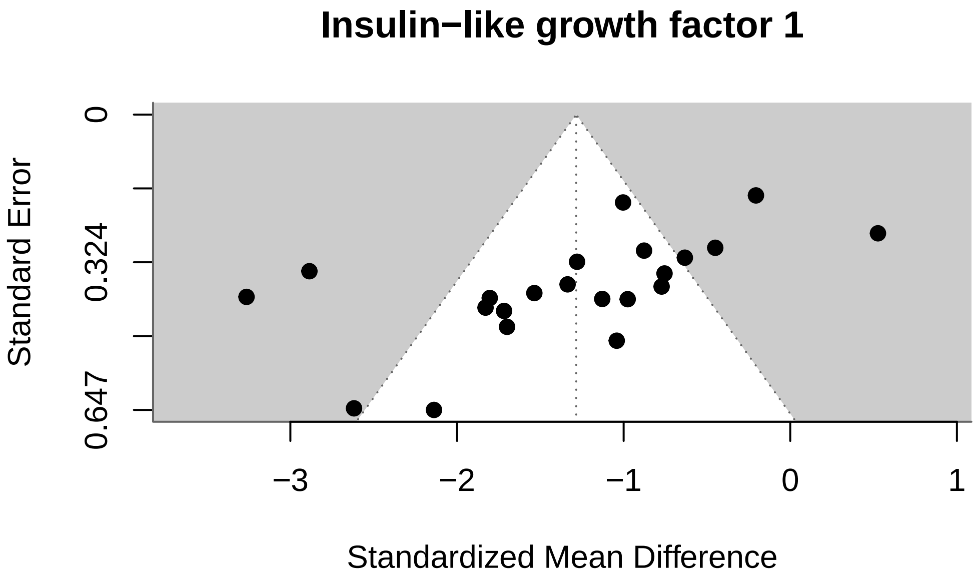
Insulin−like growth factor 1
Standard Error
0
0.324
0.647
−3
−2
−1
0
1
Standardized Mean Difference

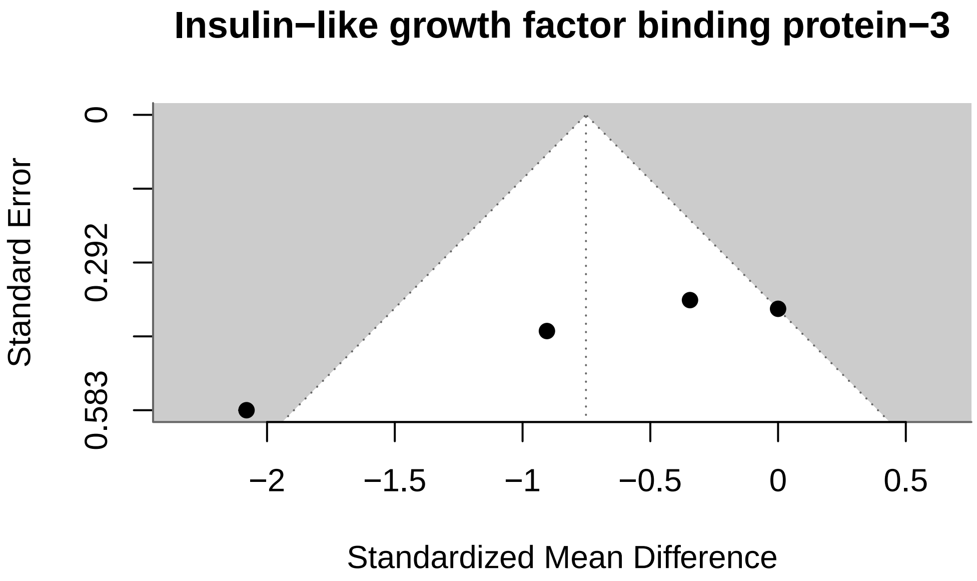
Insulin−like growth factor binding protein−3
Standard Error
0
0.292
0.583
−2
−1.5
−1
−0.5
0
0.5
Standardized Mean Difference

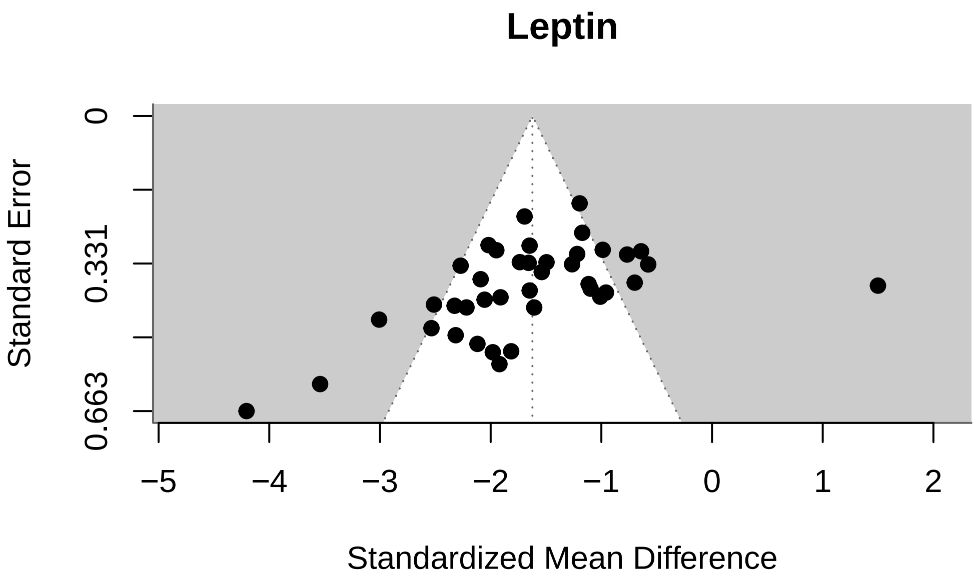
Leptin
Standard Error
0
0.331
0.663
−5
−4
−3
−2
−1
0
1
2
Standardized Mean Difference

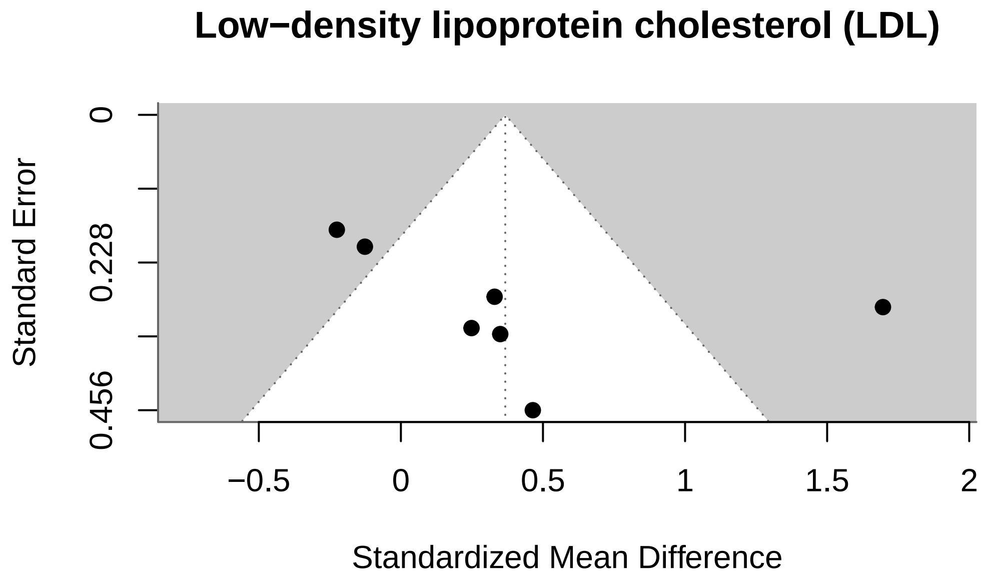
Low−density lipoprotein cholesterol (LDL)
Standard Error
0
0.228
0.456
−0.5
0
0.5
1
1.5
2
Standardized Mean Difference

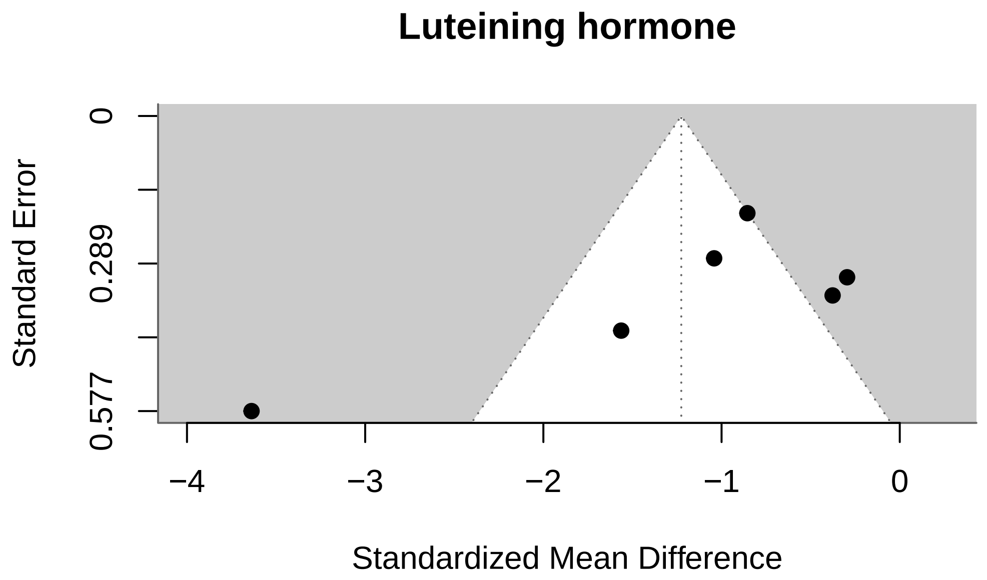
Luteining hormone
Standard Error
0
0.289
0.577
−4
−3
−2
−1
0
Standardized Mean Difference

Lymphocyte

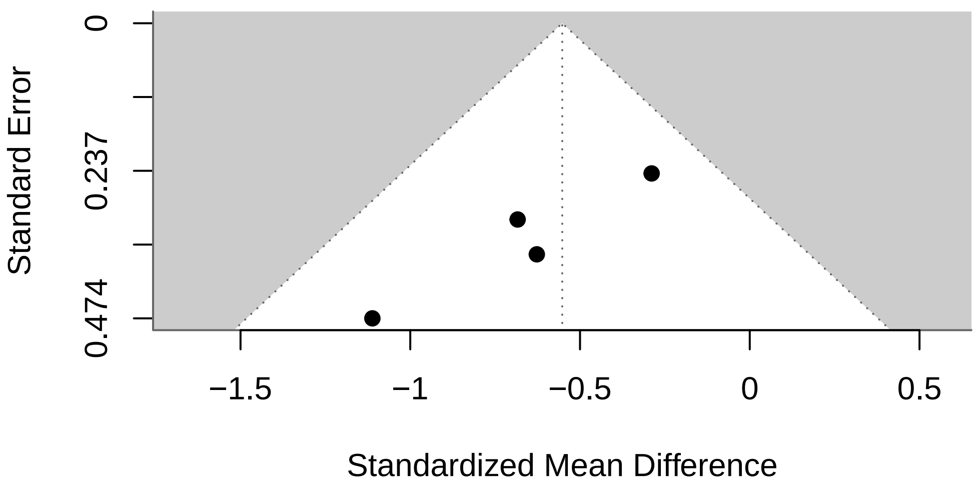

Melatonin (N−acetyl−5 methoxytryptamine)

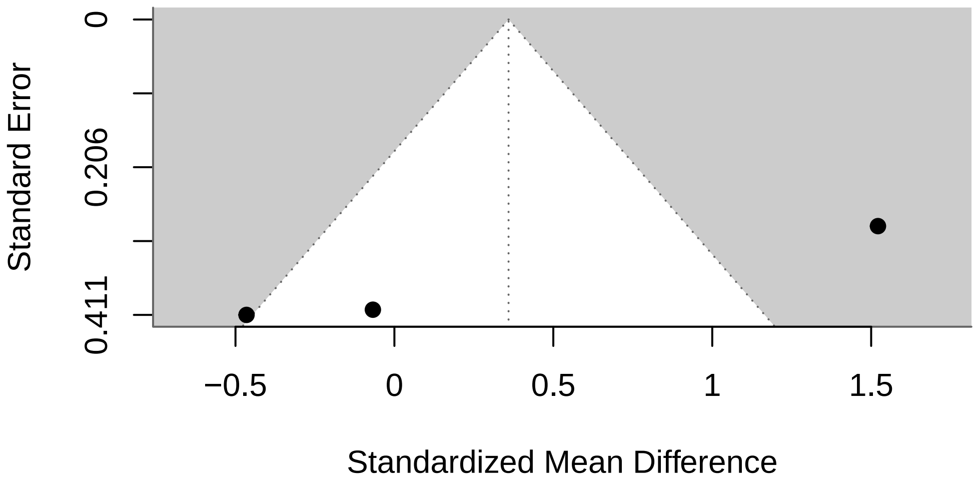

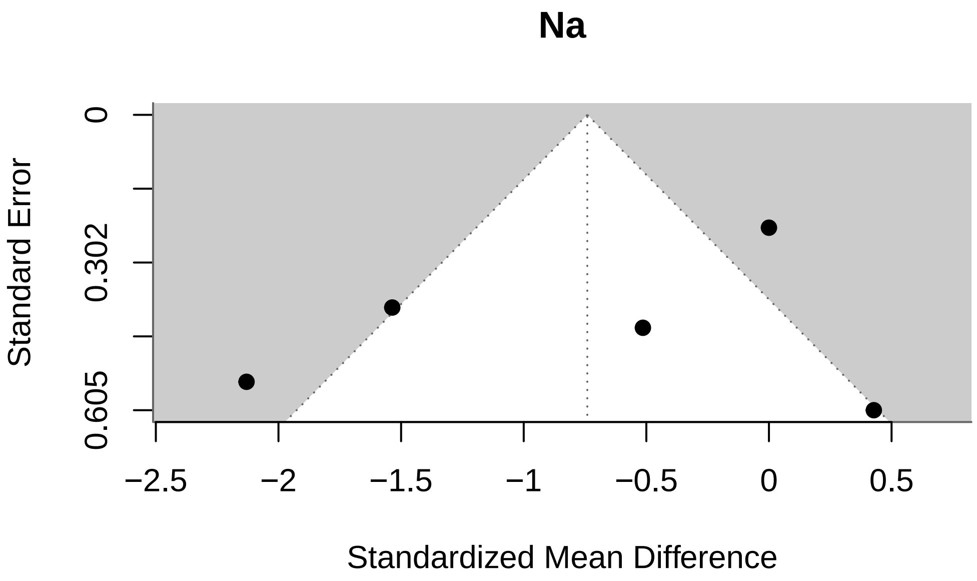
Na
Standard Error
0
0.302
0.605
−2.5
−2
−1.5
−1
−0.5
0
0.5
Standardized Mean Difference

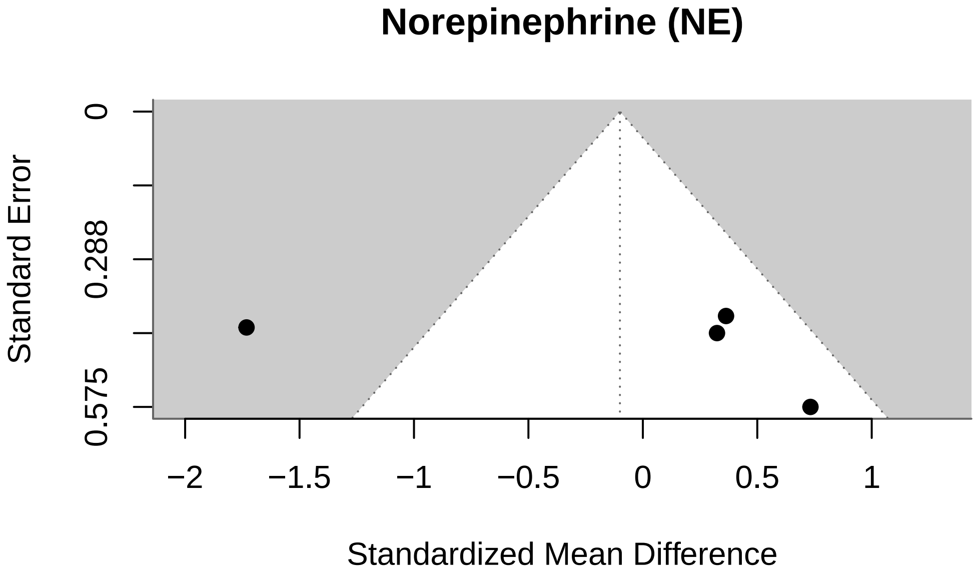
Norepinephrine (NE)
Standard Error
0
0.288
0.575
−2
−1.5
−1
−0.5
0
0.5
1
Standardized Mean Difference

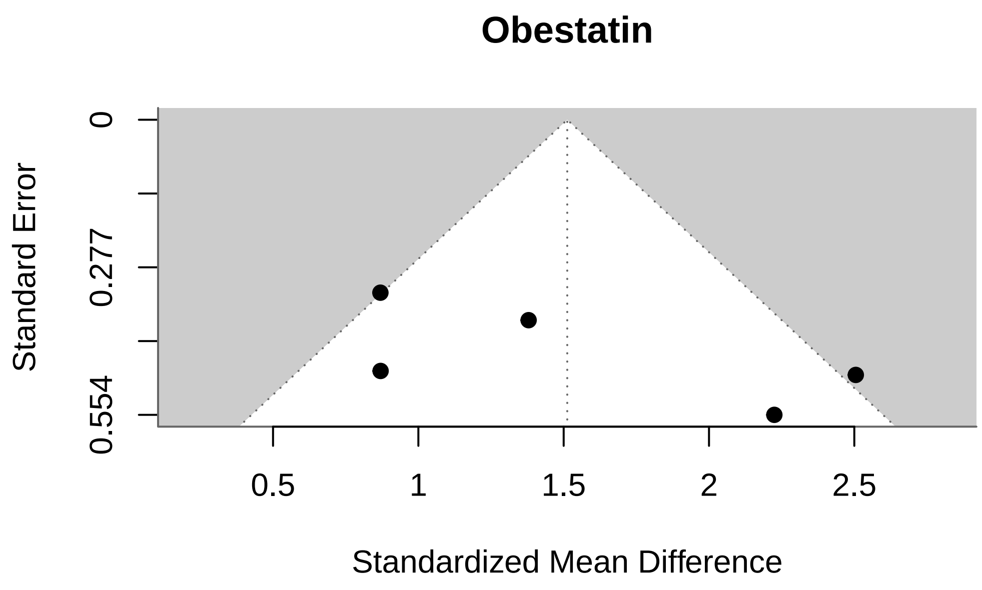
Obestatin
Standard Error
0
0.277
0.554
0.5
1
1.5
2
2.5
Standardized Mean Difference

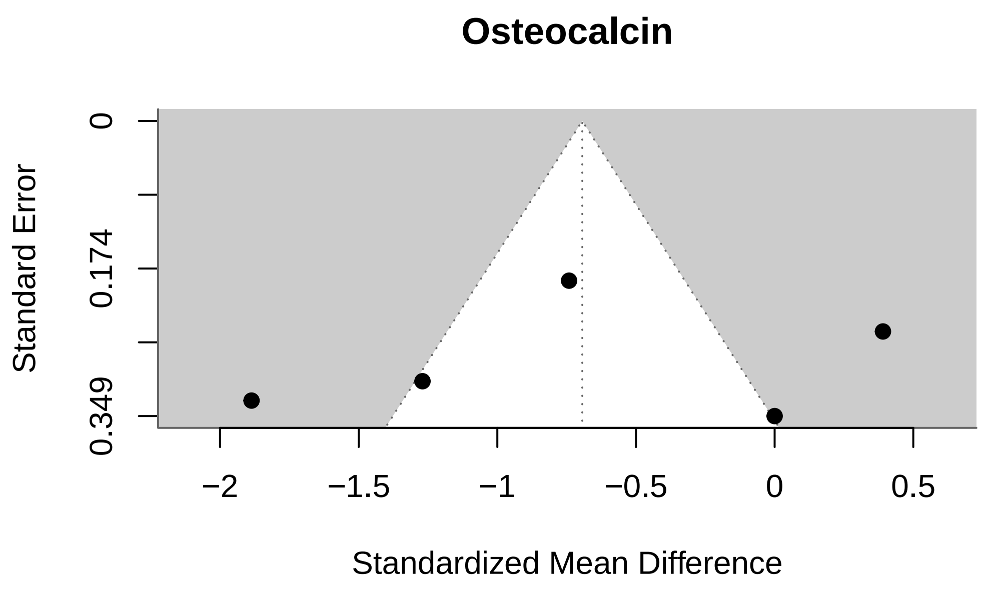
Osteocalcin
Standard Error
0
0.174
0.349
−2
−1.5
−1
−0.5
0
0.5
Standardized Mean Difference

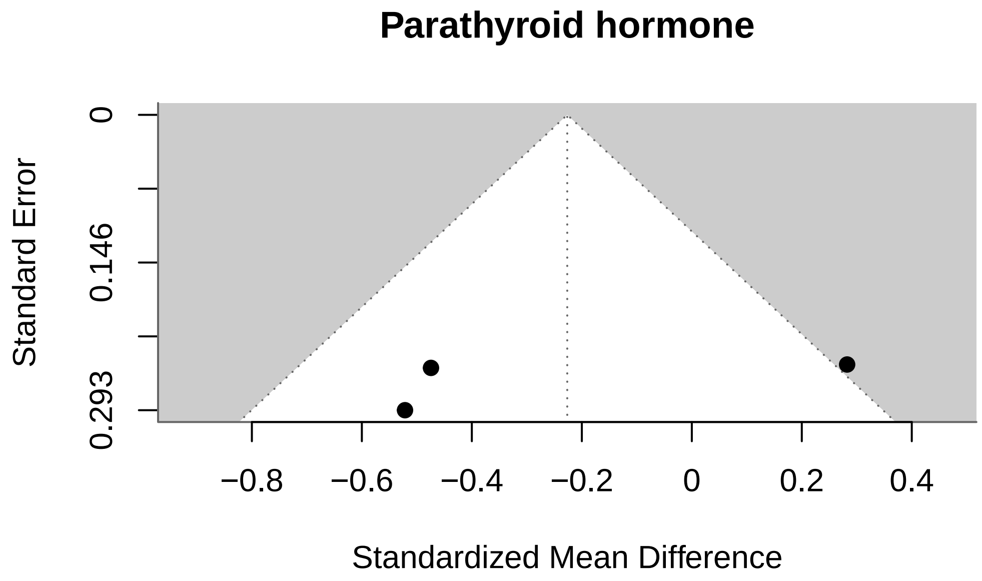
Parathyroid hormone
Standard Error
0
0.146
0.293
−0.8
−0.6
−0.4
−0.2
0
0.2
0.4
Standardized Mean Difference

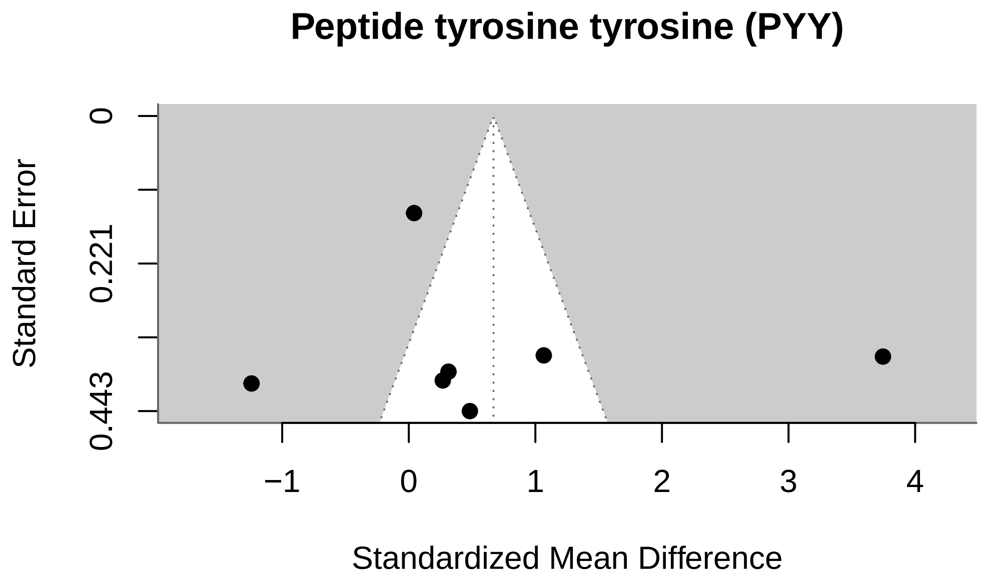
Peptide tyrosine tyrosine (PYY)
Standard Error
0
0.221
0.443
−1
0
1
2
3
4
Standardized Mean Difference

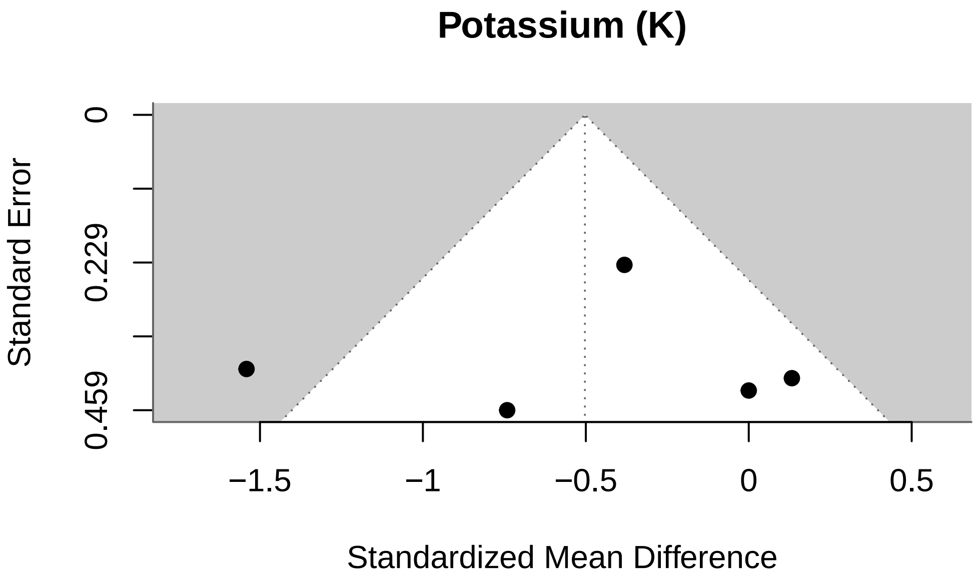

Potassium (K)
Standard Error
0
0.229
0.459
−1.5
−1
−0.5
0
0.5
Standardized Mean Difference

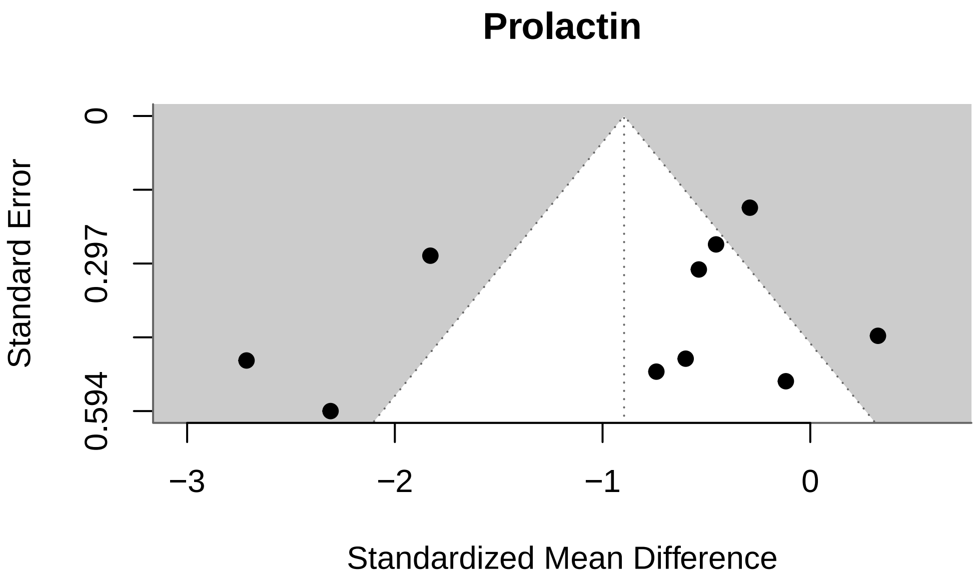

Prolactin
Standard Error
0
0.297
0.594
−3
−2
−1
0
Standardized Mean Difference

Resistin

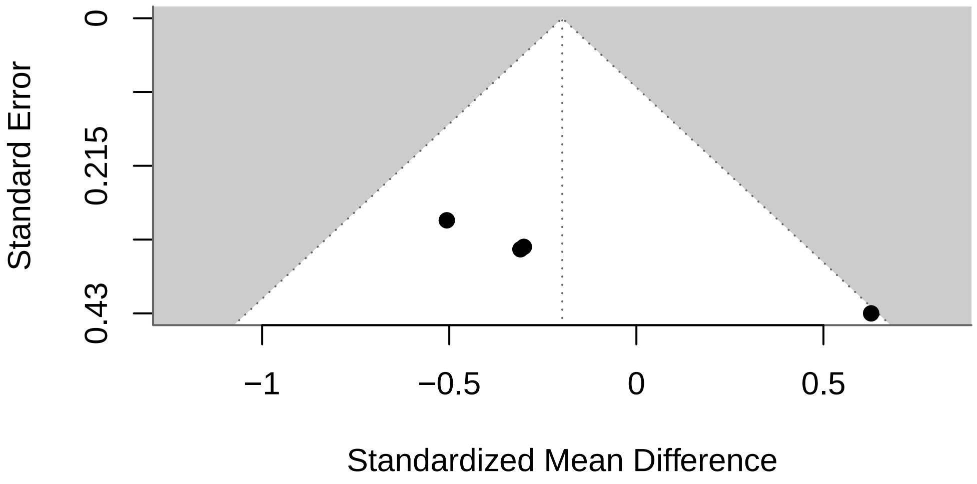

Sex hormone–binding globulin

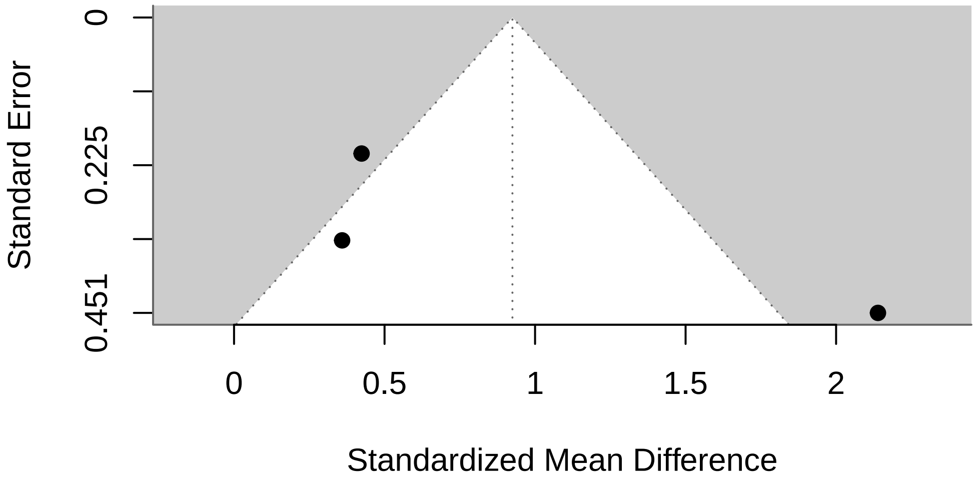

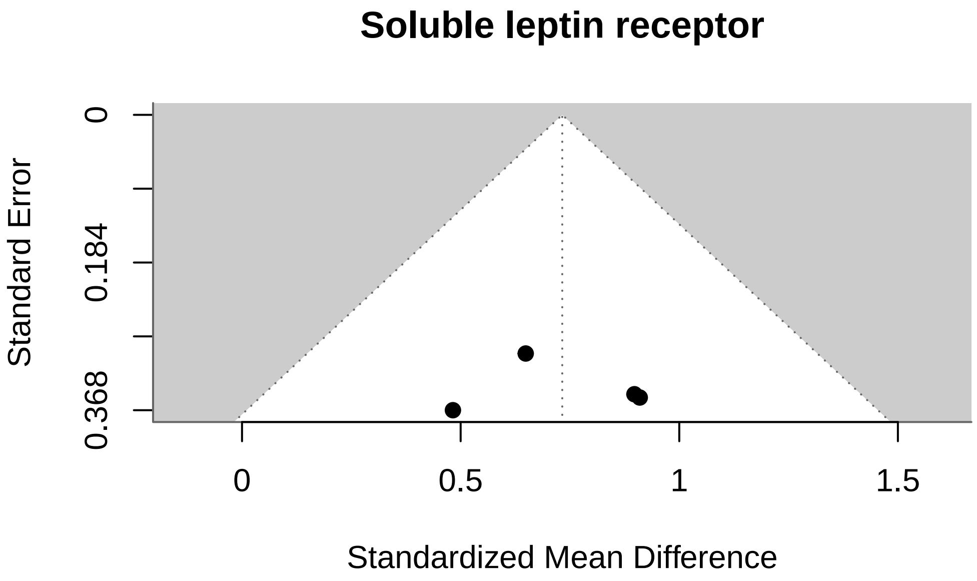
Soluble leptin receptor
Standard Error
0
0.184
0.368
0
0.5
1
1.5
Standardized Mean Difference

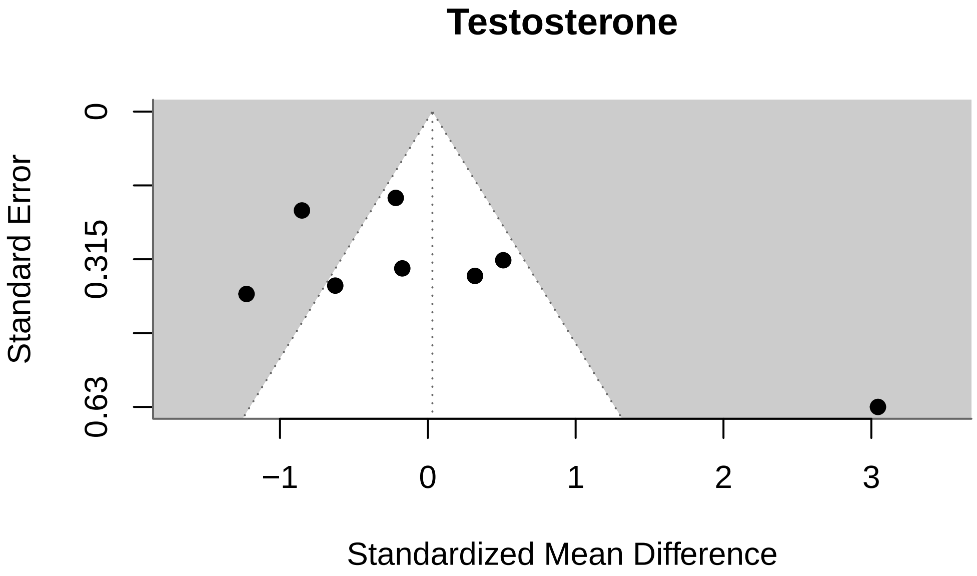
Testosterone
Standard Error
0
0.315
0.63
−1
0
1
2
3
Standardized Mean Difference

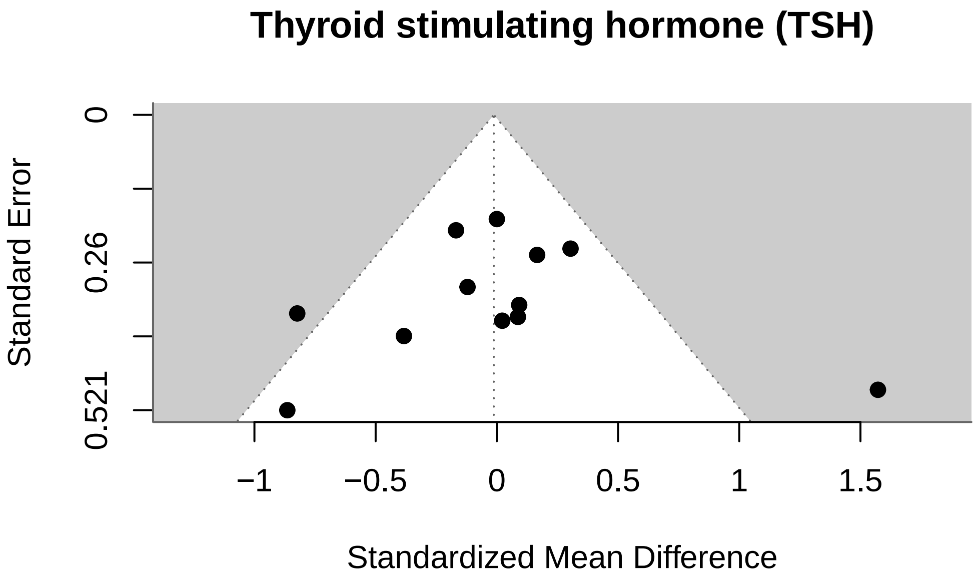

Thyroid stimulating hormone (TSH)
Standard Error
0
0.26
0.521
−1
−0.5
0
0.5
1
1.5
Standardized Mean Difference

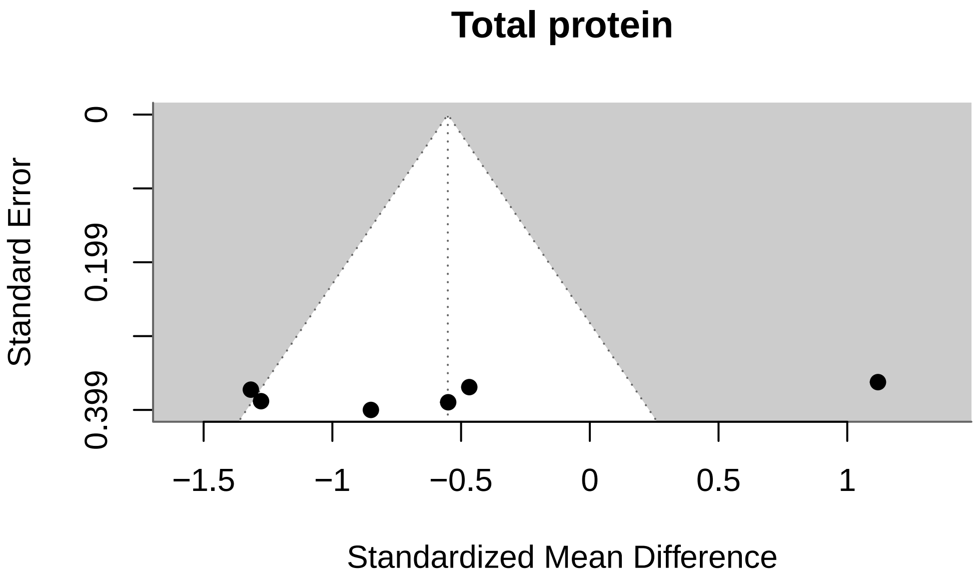

Total protein
Standard Error
0
0.199
0.399
−1.5
−1
−0.5
0
0.5
1
Standardized Mean Difference

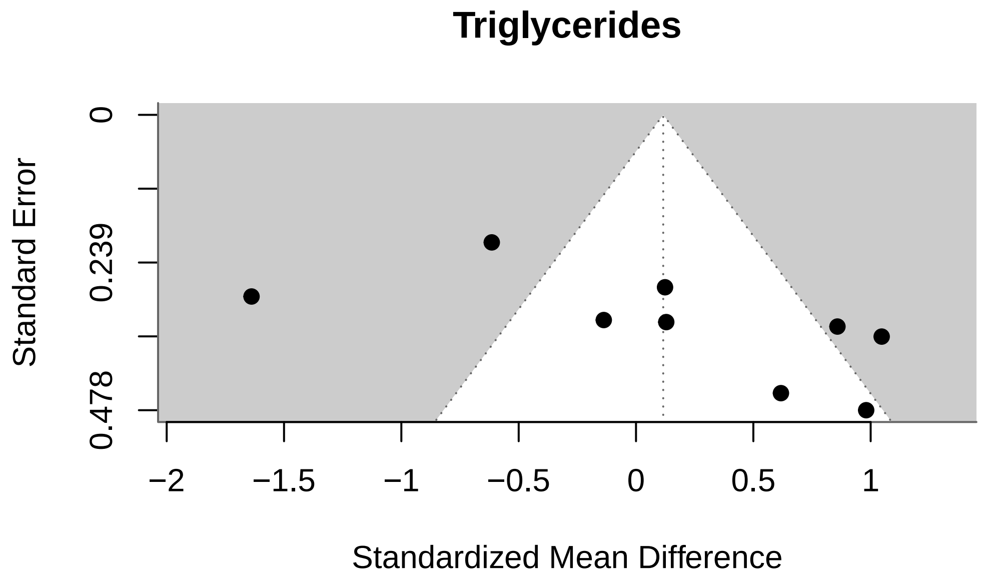
Triglycerides
Standard Error
0
0.239
0.478
−2
−1.5
−1
−0.5
0
0.5
1
Standardized Mean Difference
